# Impact of 3-deazapurine nucleobases on RNA properties

**DOI:** 10.1093/nar/gkab256

**Published:** 2021-04-15

**Authors:** Raphael Bereiter, Maximilian Himmelstoß, Eva Renard, Elisabeth Mairhofer, Michaela Egger, Kathrin Breuker, Christoph Kreutz, Eric Ennifar, Ronald Micura

**Affiliations:** Institute of Organic Chemistry, Center for Molecular Biosciences Innsbruck, University of Innsbruck, Innsbruck, Austria; Institute of Organic Chemistry, Center for Molecular Biosciences Innsbruck, University of Innsbruck, Innsbruck, Austria; Architecture et Réactivité de l’ARN - CNRS UPR 9002, Université de Strasbourg, Strasbourg, France; Institute of Organic Chemistry, Center for Molecular Biosciences Innsbruck, University of Innsbruck, Innsbruck, Austria; Institute of Organic Chemistry, Center for Molecular Biosciences Innsbruck, University of Innsbruck, Innsbruck, Austria; Institute of Organic Chemistry, Center for Molecular Biosciences Innsbruck, University of Innsbruck, Innsbruck, Austria; Institute of Organic Chemistry, Center for Molecular Biosciences Innsbruck, University of Innsbruck, Innsbruck, Austria; Architecture et Réactivité de l’ARN - CNRS UPR 9002, Université de Strasbourg, Strasbourg, France; Institute of Organic Chemistry, Center for Molecular Biosciences Innsbruck, University of Innsbruck, Innsbruck, Austria

## Abstract

Deazapurine nucleosides such as 3-deazaadenosine (c^3^A) are crucial for atomic mutagenesis studies of functional RNAs. They were the key for our current mechanistic understanding of ribosomal peptide bond formation and of phosphodiester cleavage in recently discovered small ribozymes, such as twister and pistol RNAs. Here, we present a comprehensive study on the impact of c^3^A and the thus far underinvestigated 3-deazaguanosine (c^3^G) on RNA properties. We found that these nucleosides can decrease thermodynamic stability of base pairing to a significant extent. The effects are much more pronounced for 3-deazapurine nucleosides compared to their constitutional isomers of 7-deazapurine nucleosides (c^7^G, c^7^A). We furthermore investigated base pair opening dynamics by solution NMR spectroscopy and revealed significantly enhanced imino proton exchange rates. Additionally, we solved the X-ray structure of a c^3^A-modified RNA and visualized the hydration pattern of the minor groove. Importantly, the characteristic water molecule that is hydrogen-bonded to the purine N3 atom and always observed in a natural double helix is lacking in the 3-deazapurine-modified counterpart. Both, the findings by NMR and X-ray crystallographic methods hence provide a rationale for the reduced pairing strength. Taken together, our comparative study is a first major step towards a comprehensive understanding of this important class of nucleoside modifications.

## INTRODUCTION

Deazapurine nucleosides have been intensively applied in atomic mutagenesis studies of biologically relevant RNAs (1–5). The replacement of a single nitrogen atom by a carbon atom can be critical because the acid-base properties of the nucleobase are dramatically changed and hydrogen acceptor/donor properties are erased at the specific position (6–8). This can be determining for RNA base pairing (8,9), for RNA recognition of other nucleic acids (e.g. DNA, 2′-OCH_3_ RNA) (6), proteins (3), small molecules (8), and ions (9), and this can also be crucial with respect to RNA-catalyzed reactions (4,10–13). Concerning the latter, atomic mutagenesis lead to our current in-depth understanding of the chemical mechanism of phosphodiester cleavage of the recently discovered twister and pistol ribozymes (14–16). Similarly, atomic mutagenesis of the peptidyl transferase center in the ribosome center allowed to critically evaluate the mechanistic proposals for peptide bond formation that arose from interpretations of static crystal structures (17–20). These functional assays contributed significantly to a profound comprehension of ribosomal protein synthesis.

Suitable deazanucleosides for informative RNA atomic mutagenesis experiments are 7-deazaadenosine (c^7^A) (4,11–13,15,16), 3-deazaadenosine (c^3^A) (14,20), 1-deazaadenosine (c^1^A) (11,12,14,20), 7-deazaguanosine (c^7^G) (13,15) and 3-deazacytidine (c^3^C) (13,21). Moreover, 3-deazaguanosine (c^3^G) and 1-deazaguanosine (c^1^G) would be highly useful for RNA atomic mutagenesis, however, such studies are very rare (22) because synthetic access to appropriate phosphoramidite building blocks and to the corresponding RNAs is challenging (22,23). In general, 3-deazapurine modified RNAs appear underinvestigated when compared to the 7-deazapurine modified counterparts (24–28). Besides, more is known about deaza-modified DNA compared to deaza-modified RNA (29–38).

Therefore, we set out to accomplish a thorough chemical and biophysical analysis of 3-deazapurine containing RNA, along with the improvement of the synthesis of c^3^G containing RNA beforehand. We here describe the impact of c^3^G and c^3^A on RNA properties. Based on UV-spectroscopic melting experiments, a detailed thermodynamic analysis of duplex and hairpin stabilities is provided and the effects on base pairing are discussed in the light of the sequence context. Furthermore, solution NMR spectroscopy sheds light on base pair opening dynamics. In addition, we have solved the X-ray structure of a c^3^A containing RNA at atomic resolution to disclose crucial structural features, such as ribose puckers, hydrogen-bonding networks, and hydration patterns of the deazanucleoside, and to correlate them to base pairing properties.

## MATERIAL AND METHODS

### Synthesis and characterization of organic compounds

Reagents were purchased in the highest available quality from commercial suppliers (Merck/Sigma-Aldrich, ABCR, VWR, ChemGenes, CarboSynth) and used without further purification. All reactions were carried out under argon atmosphere, unless otherwise noted. Analytical thin-layer chromatography (TLC) was performed on Macherey-Nagel Polygram^®^ SIL G/UV_254_ plates. Silica gel 60 (mesh size 0.04 – 0.063 mm) for column chromatography was purchased from Macherey-Nagel. The procedures for chemical synthesis of phosphoramidites **8** and **9**, and the 6-chloro-3-deazapurine **14** and their characterization data are available in the Supporting Information (phosphoramidite **8**: eight steps, eight chromatographic purifications, 13% overall yield; total amount synthesized: 0.90 g; phosphoramidite **9**: ten steps, 10 chromatographic purifications, 9% overall yield; total amount synthesized: 0.80 g; 6-amino-3-deazapurine **15**: 4 steps, four chromatographic purifications, 54% overall yield; total amount synthesized: 5 g). ^1^H, ^13^C, and ^31^P NMR spectra were recorded on a Bruker Ultrashield™ 400 Plus spectrometer. Chemical shifts (δ) are reported relative to tetramethylsilane (TMS), referenced to the residual solvent signal (DMSO-d_6_: 2.50 ppm for ^1^H and 39.52 ppm for ^13^C NMR spectra; CDCl_3_: 7.26 ppm for ^1^H and 77.16 ppm for ^13^C NMR spectra). The following abbreviations were used to denote multiplicities: s = singulet, d = doublet, t = triplet, q = quadruplet, m = multiplet, b = broad. Signal assignments are based on ^1^H–^1^H-COSY, ^1^H–^13^C-HSQC and ^1^H–^13^C-HMBC experiments. High resolution mass spectra were recorded in positive ion mode on a Thermo Scientific Q Exactive Orbitrap, ionized via electrospray at 3.7 kV spray voltage.

### RNA solid-phase synthesis

Standard phosphoramidite chemistry was applied for RNA strand elongation and incorporation of 3-deazaguanosine (*N^2^*-Tfa, 2′-*O*-Tbs and *N^2^*-Tfa, 2′-*O*-CEM; >98% coupling yield), 3-deazadenosine (*N^6^*-Bz, 2′-*O*-TIPS; >92% coupling yield), 7-deazaguanosine (*N^2^*-*i*Bu, 2′-*O*-Tbs; >98% coupling yield) and 7-deazaadenosine (*N^6^*-Bz, 2′-*O*-Tbs; >98% coupling yield). 2′-*O*-TOM and acetyl protected nucleoside phosphoramidite building blocks and 2′-*O*-Tbs 1000 Å CPG solid support (>15nt) were purchased from ChemGenes, Primer support^TM^ 5G (<15nt) was purchased from GE Healthcare. All oligonucleotides were synthesized on an ABI 391 or ABI 392 Nucleic Acid Synthesizer following standard methods: detritylation (90 sec) with dichloroacetic acid/1,2-dichloroethane (4/96); coupling (5.0 min) with phosphoramidites/acetonitrile (100 mM, 200 μl) and benzylthiotetrazole / acetonitrile (300 mM, 500 μl); capping (2 × 25 s) with Cap A/Cap B (1/1) for c^3^A, c^7^G & c^7^A modified RNA, Cap A: 4-(dimethylamino)pyridine/acetonitrile (500 mM), Cap B: acetic anhydride/*sym*-collidine/acetonitrile (2/3/5) and Cap A mild/Cap B mild (1/1) for c^3^G modified RNA, Cap A mild: phenoxyacetic anhydride/acetonitrile (100 mM), Cap B mild: *N*-methylimidazole/*sym*-collidine/tetrahydrofuran (0.160/0.265/10); oxidation (60 sec) with iodine (20 mM) in tetrahydrofuran/pyridine/H_2_O (35/10/5). Solutions of phosphoramidites, tetrazole and Cap were dried over activated molecular sieves (3 Å) overnight.

### Deprotection, purification and quantification of natural and modified RNA

For basic deprotection of natural, c^3^G, c^3^A, c^7^G and c^7^A modified RNA, the solid support was mixed with aqueous methylamine (40%, 0.65 ml) and aqueous ammonia (28%, 0.65 ml) for 15 min at 65°C or 3 h at 37°C. The supernatant was removed and the solid support was washed twice with 0.5 ml tetrahydrofuran/H_2_O (1/1). Combined supernatant and washings were evaporated to dryness and the residue was dissolved in a solution of tetrabutylammonium fluoride in tetrahydrofuran (1.0 M, 1.5 ml) and incubated for 14 h at 37°C for removal of 2′-*O*-silyl protecting groups. The reaction was quenched by addition of triethylammonium acetate/H_2_O (1.0 M, 1.5 ml, pH 7.4). Tetrahydrofuran was removed under reduced pressure and the sample was desalted with size-exclusion column chromatography (GE Healthcare, HiPrep™ 26/10 Desalting; Sephadex G25) eluting with H_2_O; collected fractions were evaporated and the RNA dissolved in H_2_O (1 ml). The crude RNA was purified by anion exchange chromatography on a semipreparative Dionex DNAPac® PA-100 column (9 mm × 250 mm) at 80°C with a flow rate of 1 ml/min (eluent A: 6 M urea, 25 mM Tris·HCl, pH 8.0; eluent B: 500 mM NaClO_4_, 6 M urea, 25 mM Tris·HCl, pH 8.0). Fractions containing RNA were diluted with 0.1 M triethylammonium bicarbonate solution, loaded on a C18 SepPak Plus^®^ cartridge (Waters/Millipore), washed with H_2_O and eluted with acetonitrile/H_2_O (1/1). Crude and purified RNA were analyzed by anion exchange chromatography on a GE Healthcare Äkta Explorer HPLC System containing a Dionex DNAPac^®^ PA-100 column (4 mm × 250 mm) at 80°C with a flow rate of 1 ml/min. For RNA shorter or equal to 15 nucleotides, a gradient of 0–40% B in 30 minutes and for RNA longer than 15 nucleotides a gradient of 0–60% B was used; Eluent A: 6 M urea, 25 mM Tris·HCl, pH 8.0; Eluent B: 500 mM NaClO_4_, 6 M urea, 25 mM Tris·HCl, pH 8.0. HPLC traces were recorded at UV absorption by 260 nm. RNA quantification was performed on an Implen P300 Nanophotometer.

### Mass spectrometry of oligoribonucleotides

RNA samples (3 μl) were diluted with 40 mM Na_2_H_2_(EDTA)/H_2_O (5/4) for a total volume of 30 μl, injected onto a C18 XBridge column (2.5 μm, 2.1 mm × 50 mm) at a flow rate of 0.1 ml/min and eluted with 0–100% B gradient at 30°C (eluent A: 8.6 mM triethylamine, 100 mM 1,1,1,3,3,3-hexafluoroisopropanol in H_2_O; eluent B: methanol). RNA HPLC runs were analyzed on a Finnigan LCQ Advantage Max electrospray ionization mass spectrometer with 4.0 kV spray voltage in negative mode. Characterization of the c^3^G modified 47mer was performed on a 7 T FT-ICR-MS (Bruker Daltonics – Apex Ultra 70). Sample preparation: 2 μM RNA in 1:1 H_2_O/CH_3_OH with 20 mM imidazole and 20 mM piperidine (pH ∼ 10). The sample was measured in negative ion mode at a flow rate of 90 μl/h and a skimmer potential of –50 V for ESI and –60 V for CAD (spectra averaged CAD: 400, spectra averaged ESI: 50, capillary voltage 3.1 kV, spray shield 2.7 kV, dry temperature 200°C. A collision energy of 12 V was applied in the CAD experiments.

### Melting curve measurements of oligoribonucleotides

RNA samples were lyophilized as triethylammonium salts, dissolved in 800 or 330 μl buffer (10 mM Na_2_HPO_4_ (pH 7.0) containing 150 mM NaCl) and transferred into UV permeable high precision cells made of quartz SUPRASIL^®^ with a light path of 10 mm or 1 mm. UV melting profiles were recorded at 250 and 260 nm on a Varian Cary-100 spectrophotometer equipped with a multiple cell holder and a peltier temperature control device. Each RNA was measured at five different concentrations ranging from 1 to 100 μM. *T*_m_ values were determined by calculating the first derivative. Thermodynamic parameters were determined according to (49,50). The error limits for Δ*G*, Δ*H* and Δ*S* reflect the standard deviation of three (duplex) or five (hairpin) independent measurements for a confidence interval of 95%.

### NMR experiments

RNA samples were lyophilized as triethylammonium salts, dissolved in 500 μl NMR buffer (15 mM Na[AsO_2_(CH_3_)_2_]·3H_2_O, 25 mM NaCl, 3 mM NaN_3_, in H_2_O/D_2_O 9:1, pH 6.5) and transferred into 5 mm NMR tubes. Sample concentrations varied between 0.05 and 0.3 mM and experiments were run at 298 K unless otherwise stated. All NMR experiments were conducted on a Bruker 600 MHz Avance II+ NMR or a 700 MHz Avance Neo NM both equipped with a Prodigy TCI probe.

#### Determination of imino proton water exchange rates by CLEANEX-PM experiment

The CLEANEX-PM NMR experiments were carried out in the following buffer: 15 mM Na[AsO_2_(CH_3_)_2_]·3H_2_O, 25 mM NaCl, 3 mM NaN_3_, in H_2_O/D_2_O 9:1, pH 6.5. The same buffer stock solution was used for all samples to rule out changes in imino proton—bulk water exchange rates due to differences in the buffer composition. The CLEANEX-PM pulse sequence is available from the Bruker standard experiment collection (*zgcxesgp*) with an excitation sculpting water suppression element. A standard excitation sculpting water suppression experiment (*zgesgp*) was used as the reference experiment. For the determination of the water *T*_1_ relaxation times under the experimental conditions a saturation recovery experiment was used and the longitudinal water relaxation time ranged between 3.03 s (c^3^G RNA) and 3.13 s (unmodified RNA). The following experimental parameters were used: spectral width 24 ppm, o1p: 4.7 ppm, number of scans 2048, dummy scans 32, interscan delay 1.5 s. Shaped pulse parameters were set via the *getprosol* command and the hard 90° ^1^H pulse. The mixing times were set to 5, 25, 50, 50, 100, 100, 150, 200, 300, 400, 400 and 500 ms for both duplexes. The NMR spectra were processed in TOPSPIN 4.0.9 using a line broadening factor of 10 Hz (lb value set to 10). The absolute peak intensities of the CLEANEX-PM experiments and the reference experiments were determined and used to obtain the relative intensities. The data was then exported to MATLAB and the build-up curves were fitted to the following equation:

$$\begin{eqnarray*} {I \mathord{\left/ {\vphantom {I {{I_0}}}} \right. } {{I_0}}} &=& \frac{k}{{\left( {{R_1}A + k - {R_{1,water}}} \right)}} \nonumber \\ &&\times\,\left\{ {{e^{ - {R_{(1,water)}}{\tau _{mix}})}} - {e^{\left( { - {R_{1,A}} + k} \right)}}{\tau _{mix}}} \right\} \end{eqnarray*}$$

with *I*/*I*_0_ relative peak intensity, *k* imino proton–bulk water exchange rate (s^−1^), *R*_1,water_ longitudinal water exchange rate, $\tau$_mix_ CLEANEX-PM mixing times (s) and *R*_1,A_ is a combination of the longitudinal and transverse relaxation rate of the imino proton resonance (floating parameter during fitting). Errors in exchange rates were estimated from replicate experiments and obtained from 1000 Monte-Carlo runs.

#### Determination of the pK_a_ value of 3-deazaguanosine by NMR spectroscopy

For the pH dependent ^1^H and ^13^C NMR experiments, 22 mg of 3-deazaguanosine were dissolved in 600 μl buffer (15 mM sodium phosphate, 25 mM NaCl, 0.1 mM EDTA, pH 6.9) and transferred into a 5 mm NMR tube. The chemical shifts of C2 and C6 were recorded on a Bruker 600 MHz Avance II+ equipped with a Prodigy TCI probe. The pH-value was determined using a Sigma-Aldrich micro pH combination glass electrode. At each pH-value, ^1^H NMR spectrum and ^13^C NMR spectra at natural abundance with power gated decoupling were measured.

The p*K*_a_ values were obtained by fitting the data to the following equation:

$$\begin{equation*}\;{\delta _{obs}} = \frac{{{\delta _{deprotonated}}\;x\;{{10}^{\left( {pH - p{k_a}} \right)}} + \;{\delta _{protonated}}}}{{1 + \;{{10}^{\left( {pH - p{k_a}} \right)}}}}\;\end{equation*}$$

With ${\delta _{obs}}$ observed ^13^C chemical shift at the respective pH value, ${\delta _{deprotonated}}$^13^C chemical shift of deprotonated species and ${\delta _{deprotonated}}$^13^C chemical shift of the protonated species.

### Crystallization and structure solution of a c^3^A modified RNA

Oligoribonucleotides of 27 nucleotide length corresponding to the sequence of *Escherichia coli* 23 S rRNA sarcin-ricin loop (SRL) and containing a 3-deazaadenosine at position 2670 were used for crystallization. The RNA was dissolved at a concentration of ∼350 μM in a buffer made of Tris–HCl (10 mM), Na_2_H_2_EDTA (1 mM), pH 8.0. The RNA sample was then heated to 55°C and cooled down to 10°C using a temperature-controlled device equipped with a Peltier element. Only one unique cubic-shaped crystal of c^3^A2670-modified RNA could be obtained. It grew after one month at 20°C using vapor diffusion method by mixing two volumes of RNA sample with one volume of a crystallization buffer made of ammonium sulfate (2.5 M), magnesium acetate (10 mM), and 2-(*N*-morpholino)ethanesulfonic acid (MES) (50 mM), pH 5.6 (the other drops made in identical conditions led to spherullites). Prior data collection, the crystal was cryoprotected for about 5 min in a reservoir solution containing 15% of glycerol and 3.0 M of ammonium sulfate, flash-frozen in liquid ethane and then transferred into liquid nitrogen. The collection of X-ray diffraction data has been done on the X06DA beamline at the SLS synchrotron, Villigen, Switzerland. Processing of the data was done with the XDS Package (71) and the structure was solved by molecular replacement with MOLREP (72) using the related PDB ID 3DVZ unmodified SRL RNA model. The structure was refined with the PHENIX package (73). Models were built using Coot (74). Alternative conformations were visible for residues 2647–2650. Coordinates have been deposited with the PDB database (PDB ID 7L3R for c^3^A2670-modified SRL).

## RESULTS AND DISCUSSION

### Improved c^3^G building block with *N*^2^-Tfa and 2′-OTbs protection

For the solid-phase synthesis of c^3^A and c^3^G modified RNA, appropriate phosphoramidite building blocks are needed. Their syntheses have been a bottleneck for a long time (18,22), with new and more powerful routes coming up only recently (23,28).

A practical synthesis for c^3^G phosphoramidites from inexpensive starting materials has been introduced by our research group (23). Therein, the phenoxyacetyl (Pac) and 2-cyanoethoxymethyl (Cem) groups block the *N*^2^- and 2′-hydroxyl functionalities, while the *O*^6^ atom remains unprotected. This building block was successfully incorporated into a short 5 nt RNA strand. Deprotection of the *N*^2^-phenoxyacetyl group, however, requires rather long reaction times and high pH values which causes degradation when longer oligoribonucleotides are envisaged. To solve this problem, we here demonstrate the installation of a more base-labile protection group for the exocyclic amine of c^3^G, resulting in *N*^2^-trifluoroacetyl protected 3-deazaguanosine phosphoramidite **8**. Distinct to our previously introduced c^3^G building block, we used 2′-*O*-*tert*.-butyldimethylsilyl (Tbs) protection (instead of the 2′-*O*-Cem group) to guarantee widest possible compatibility with standard RNA solid-phase synthesis.

**Scheme 1.**
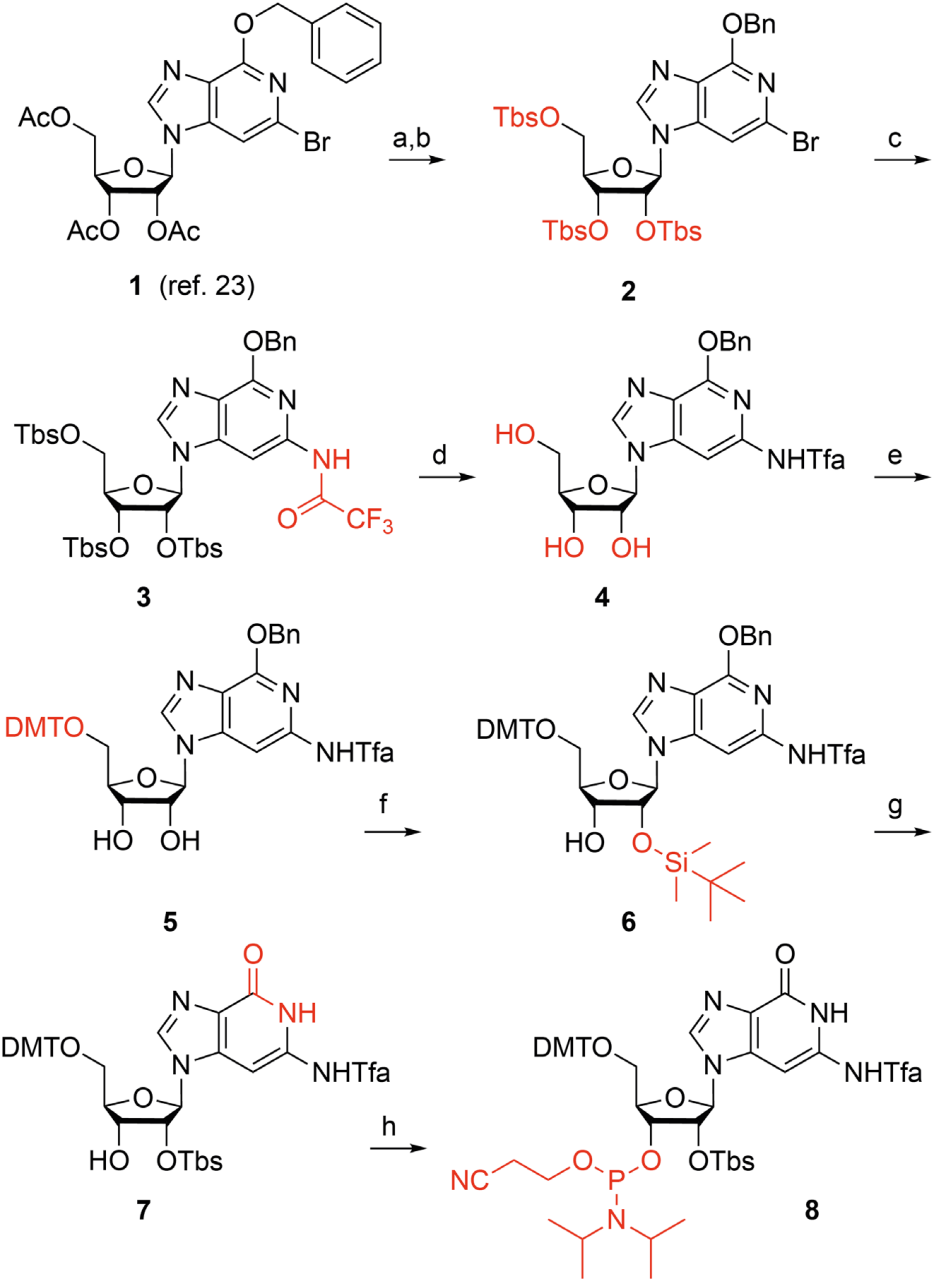
Synthesis of the novel c^3^G phosphoramidite building block **8**. Reagents and conditions: (a) MeNH_2_ in ethanol, room temperature, 1 h, 99%; (b) 6 equiv *tert*-butyldimethylsilyl chloride (TbsCl), 7 equiv imidazole in DMF, room temperature, 72 h, 90%; (c) 1.2 equiv trifluoroacetamide, 1.4 equiv Cs_2_CO_3_, 0.05 equiv Pd_2_(dba)_3_, 0.15 equiv Xantphos in 1,4-dioxane, 110°C, 5 h, 90%; (d) 4.1 equiv tetra-*n*-butylammonium fluoride trihydrate in tetrahydrofuran, room temperature, 2 h, 87%; (e) 1.15 equiv 4,4′-dimethoxytriphenylmethyl chloride, 0.05 equiv 4-(*N*,*N*-dimethylamino)pyridine in pyridine, room temperature, 16 h, 74%; (f) 1.9 equiv TbsCl, 1.9 equiv AgNO_3_, in tetrahydrofuran, room temperature, 16 h, 39% (after two rounds of 2′,3′ equilibration); (g) 0.33 equiv Pd/C 10%, H_2_ in tetrahydrofuran/ethanol 4:1, room temperature, 4 h, 79%; (h) 3.0 equiv 2-cyanoethyl-*N*,*N*,*N*′,*N*′-tetraisopropylphosphorodiamidite, 0.5 equiv 5-(benzylthio)-1*H*-tetrazole in dichloromethane, room temperature, 18 h, 79%.

The synthesis started from the previously reported 3-deazaguanosine key intermediate **1** (23) (Scheme 1). The application of the temporary *O*-*tert*.-butyldimethylsilyl (Tbs) instead *O*-acetyl protection (compound **2**) was necessary because of otherwise partial acetyl transfer to the *N*^2^ amino group under the conditions required for the next step, namely the installation of the *N*^2^-trifluoroacetylamino (Tfa) functionality: Pd catalyzed cross coupling between trifluoroacetamide and bromo compound **2** by using Buchwald-Hartwig conditions gave derivative **3** in excellent yields. Deprotection of the Tbs groups proceeded smoothly with tetra-*n*-butylammonium fluoride (TBAF) in tetrahydrofuran to furnish compound **4**. Then, the functionalization of **4** into the desired c^3^G phosphoramidite **8** required four more transformations. First, nucleoside **4** was converted into the dimethoxytritylated compound **5**, using 4,4′-dimethoxytriphenylmethyl chloride in pyridine. Then, Tbs-protection according to Ogilvie (39) with silver nitrate proceeded with rather low stereoselectivity (about 3:2), but in combination with base-induced equilibration the overall yield was increased to 39% for compound **6**. At this point, we note that we did not follow up our initial attempts to take advantage of the Beigelman 5′,3′-silyl clamp and 2′-*O*-Tbs protection concept (40) for compound **1** because of low yields. The *O*^6^-benzyl group of nucleoside **6** was cleaved by Pd/C-catalyzed hydrogenation to provide the free lactam moiety in derivative **7**. Finally, phosphitylation was executed with 2-cyanoethyl-*N*,*N*,*N*′,*N*′-tetraisopropylphosphorodiamidite in the presence of 5-(benzylthio)-1*H*-tetrazole. Starting from nucleoside **1**, our route provides phosphoramidite **8** with 13% overall yield in eight steps involving eight chromatographic purifications; in total, 0.90 g of compound **8** was obtained during the course of this study.

**Scheme 2.**
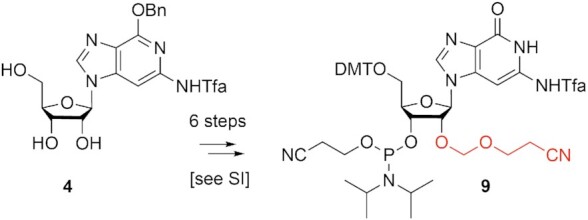
Synthesis of the novel c^3^G phosphoramidite building block **9**. For individual reaction steps, reagents and conditions see the Supporting Information.

Additionally, we synthesized the *N*^2^-Tfa protected building block **9** to take advantage of the less bulky 2′-*O-*(2-cyanoethoxy)methyl protecting group if short coupling times during solid-phase synthesis would be needed (see the Supporting Information and Scheme 2).

### Synthesis of c^3^G containing RNA

**Figure 1.**
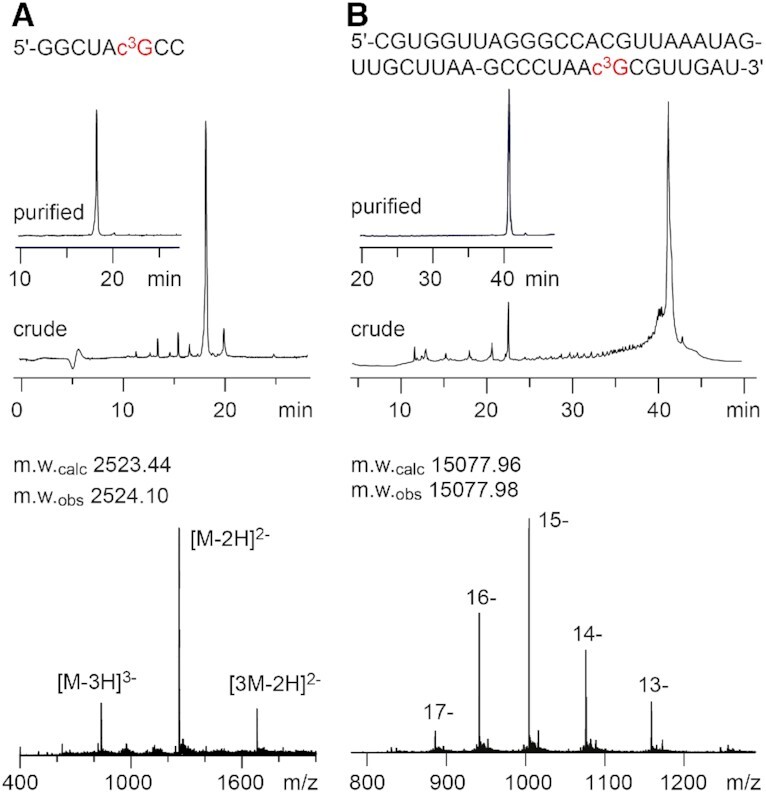
Characterization of c^3^G-modified RNA. (**A**) Anion-exchange HPLC traces of 8 nt RNA (top) and LC–ESI iontrap mass spectrum (bottom); (**B**) Anion-exchange HPLC traces of 47 nt RNA (top) and ESI FT-ICR mass spectrum (bottom). HPLC conditions: Dionex DNAPac column (4 × 250 mm), 80°C, 1 ml min^–1^, 0–40% buffer B (for 8 nt RNA) in buffer A within 30 min; 0–60% buffer B (for 47 nt RNA) in buffer A within 30 min; buffer A: Tris–HCl (25 mM), urea (6 M), pH 8.0; buffer B: Tris-HCl (25 mM), urea (6 M), NaClO_4_ (0.5 M), pH 8.0. See the experimental for LC–ESI MS conditions.

The incorporation of building blocks **8** and **9** into oligoribonucleotides proceeds with high coupling rates and excellent yields using standard RNA solid-phase synthesis protocols and, as intended, significantly milder conditions can be used to reach complete deprotection of the c^3^G containing oligonucleotides. Figure 1 exemplarily illustrates the synthesis of 8 and 47 nt long RNA oligonucleotides with a single c^3^G modification (for a complete list of synthesized oligos see Supplementary Table S1). In short, the c^3^G phosphoramidite building blocks **8** or **9** were applied in combination with standard *N*-acetylated 2′-*O*-[(triisopropylsilyl)oxy]methyl (TOM) phosphoramidites and the oligomers were assembled on controlled pore glass (CPG) supports (41). Cleavage from the solid support and deprotecting of the base labile groups were accomplished by treatment with methylamine/ammonia in water (1:1 mixture of 40% aqueous methylamine and 30% aqueous ammonia (AMA) for 3 h at 37°C room temperature or 15 min at 65°C). Subsequently, deprotection of the 2′-*O*-silyl groups (and the 2′-*O*-Cem group in case of **9**) was carried out with tetra-*n*-butylammonium fluoride trihydrate in tetrahydrofuran for 14 hours at 37°C. The reaction was quenched by the addition of triethylammonium acetate buffer at pH 7.4. Salts were removed by size-exclusion chromatography. Analysis by anion exchange chromatography under strong denaturing conditions usually gave a major peak for the desired RNA which was further purified by anion exchange chromatography on a semipreparative column. The molecular weights of the purified RNAs were confirmed by LC–ESI-MS (Supplementary Table S1) and the sequences were confirmed by top-down mass spectrometry (MS) using a Fourier-transform ion cyclotron resonance (FT-ICR) mass spectrometer (42) (Supplementary Table S2).

### Synthesis of c^3^A containing RNA

**Scheme 3.**
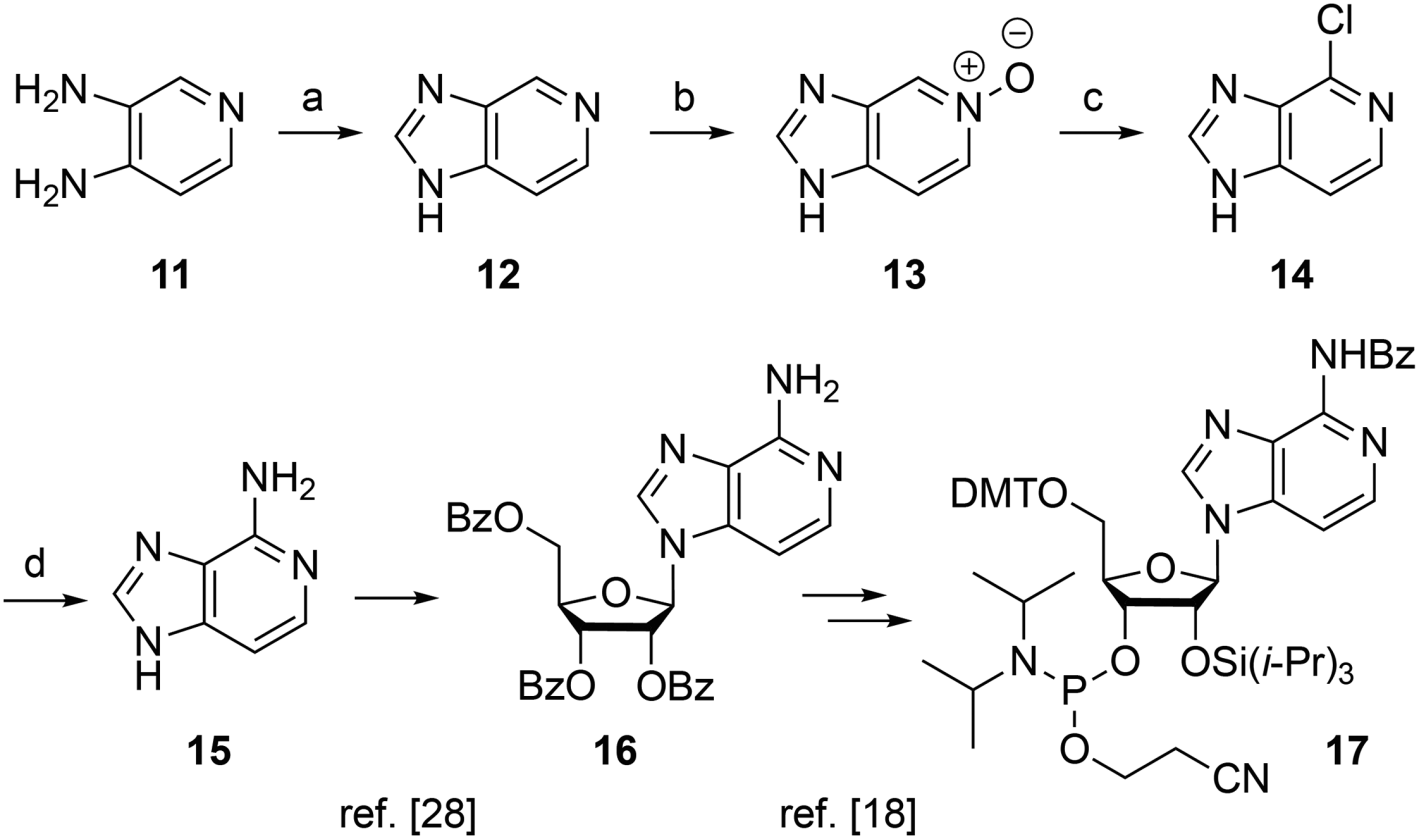
Synthesis of the novel c^3^A phosphoramidite building block **16**. Reagents and conditions: (a) in formic acid, 140°C, 48 h, 97%; (b) 2.0 equiv 3-chloroperbenzoic acid in 2:1 (v/v) CH_2_Cl_2_/CH_3_OH, room temperature, 16 h, 96%; (c) in POCl_3_, 110°C, 4 h, 67%; (d) in 1:1 (v/v) 30% NH_3_ in H_2_O/1,4-dioxane, 190°C, autoclave, 120 h, 87%.

The synthetic path of the c^3^A nucleoside building block has been merged from two routes that we published earlier (Scheme 3) (18,28). The key reaction is a silyl-Hilbert–Johnson nucleosidation using unprotected 6-amino-3-deazapurine **15** and benzoyl-protected 1-*O*-acetylribose (28). We then proceeded with *N*^6^-benzoyl protection (18) instead of *N*^6^-(di-*n*-butyl)amidine protection (28) for the following reason: The introduction of the 2′-*O*-triisopropylsilyl group is not regioselective and results in a mixture of 2′- and 3′-*O*-regioisomers; the required chromatographic purification of this mixture is more straightforward for the *N*^6^-benzoyl protected regioisomers (compared to the *N*^6^-(di-*n*-butyl)amidine counterparts) because of the significantly larger gap in retention times. Furthermore, in this work we also optimized the access to 6-amino-3-deazapurine **15** and its precursor 6-chloro-3-deazapurine **14** on large scale and starting from cheap materials (Scheme 3) (43,44). Solid-phase synthesis of c^3^A-modified building block **17** into RNA was performed using standard protocols as described earlier (15).

### Base pairing properties of c^3^G and c^3^A containing RNA

**Figure 2.**
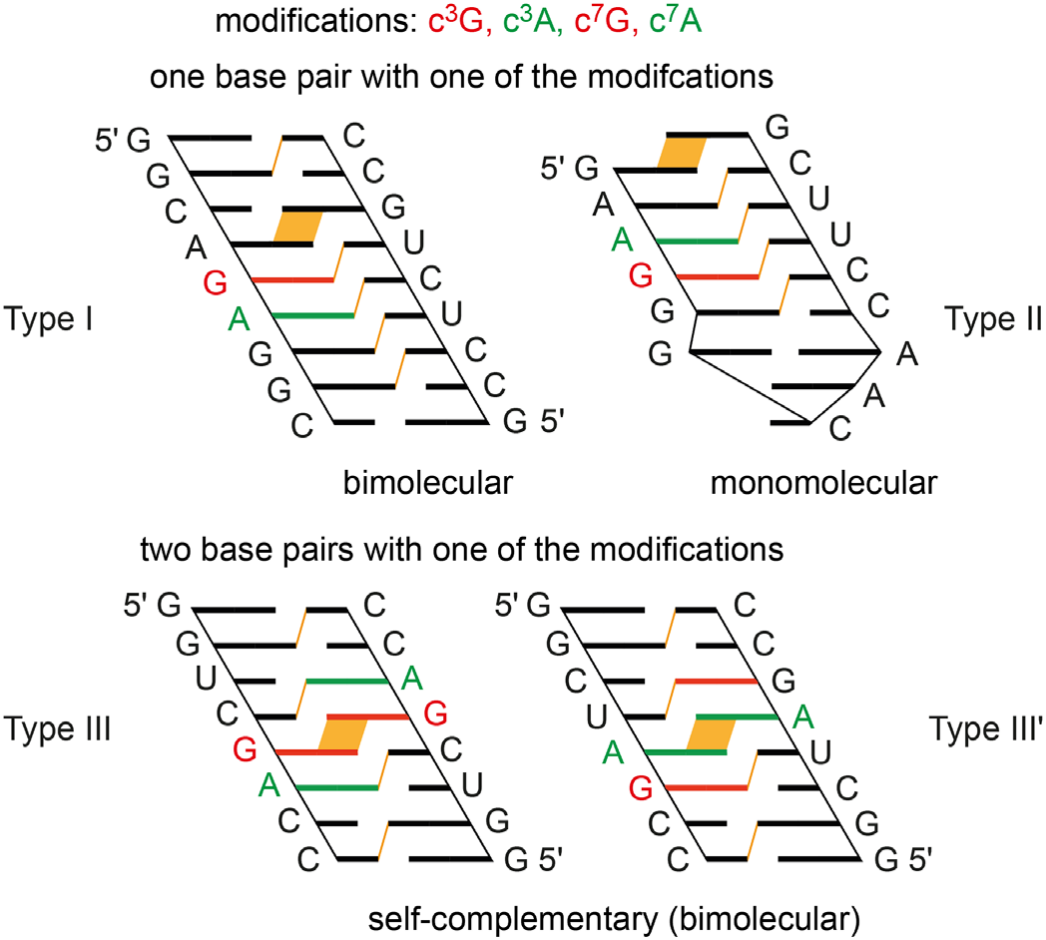
Sequence design for thermodynamic analysis of base pairing of deazapurine modified RNAs. Cartoon presentation to highlight inter-strand stacking interactions (in orange).

To the best of our knowledge, hardly anything is known about the impact of 3-deazapurine nucleosides on base pairing in regular RNA. We therefore set out to analyze the influence of c^3^G and c^3^A on the stability of RNA double helices, and additionally, to compare them to the c^7^G and c^7^A modified counterparts. Figure 2 illustrates the sequence design of the double helices investigated. The first motif constitutes an asymmetric bimolecular duplex of nine base pairs with a single deazapurine modification in the center (Type I). The second motif forms a hairpin with an extra-stable GNRA loop (GCAA), a five base pair stem with the modification again in the center, and additionally a 3′-dangling guanosine to reduce fraying of the terminal base pair (Type II). The third RNA motif covers two palindromic RNAs of eight base pairs and identical pyrimidine–purine stacking patterns (Type III and III′) with the purine modification either side-by-side in the center (thereby enabling direct inter-strand stacking), or separated by two base pairs. The latter sequence design is highly sensitive for the thermodynamic impact arising from a modification because only two and three regular Watson–Crick base pairs can form next to them: we remind that nucleation of a bimolecular double helix of oligonucleotides becomes thermodynamically favorable only when at least (three to) four continuous Watson–Crick base pairs can form (45,46). Therefore, these palindromic RNAs are expected to markedly respond to a nucleobase modification expressed in significant alterations of their thermal stabilities (*T*_m_) and thermodynamic parameters (Δ*G*, Δ*H*, Δ*S*).

Table 1 summarizes the thermal and thermodynamic data we obtained for the four RNA systems by UV-spectroscopic melting profile measurements (for melting profiles see the Supplementary Figures S1 to S16). The native type I RNA melts at 66.7°C. Both 3-deazapurines exhibit a destabilization, by 3.9 and 2.9°C, for c^3^G (in **Ia**) and c^3^A (in **Ib**) respectively. Interestingly, in this sequence context, the corresponding 7-deazapurines (c^7^G in **Ic** and c^7^A **Id**) have negligible effect on the *T*_m_ and thermodynamic parameters. For the hairpin RNA (type II), the destabilization of both 3-deazapurines is significant, reflected in a decrease of the *T*_m_-value by 9.1 and 7.9°C, for c^3^G (in **IIa**) and c^3^A (in **IIb**) respectively, when compared to the native hairpin **II**. In contrast to type I RNA, the monomolecular system (type II) reveals a slight destabilizing effect of 7-deazapurines as well (c^7^G in **IIc** and c^7^A **IId**).

**Table 1.** Thermodynamic data of deazapurine modified RNA^a^

No.	RNA sequences 5′ to 3′	*T*_m_ (°C) ^b^	Δ*T*_m_ (°C)	Δ*G*°298 (kcal mol^−1^) ^c^	Δ*H*°298 (kcal mol^−1^) ^c^	Δ*S*° (cal mol^−1^ K^−1^) ^c^
**I**	GGCAGAGGC / GCCUCUGCC	**66.7**	-	–16.5 ± 0.4	–79.7 ± 4.6	–212 ± 14
**Ia**	GGCAc^3^GAGGC / GCCUCUGCC	**62.8**	–3.9	–15.2 ± 0.5	–74.8 ± 4.7	–199 ± 14
**Ib**	GGCAGc^3^AGGC / GCCUCUGCC	**63.8**	–2.9	–16.0 ± 0.3	–80.0 ± 1.8	–215 ± 5
**Ic**	GGCAc^7^GAGGC / GCCUCUGCC	**67.2**	+0.5	–16.7 ± 0.6	–80.1 ± 5.4	–213 ± 16
**Id**	GGCAGc^7^AGGC / GCCUCUGCC	**66.5**	–0.2	–16.1 ± 0.6	–76.6 ± 4.2	–203 ± 12
**II**	GAAGGGCAACCUUCG	**73.3**	-	–7.0 ± 0.2	–52.8 ± 1.3	–153 ± 4
**IIa**	GAAc^3^GGGCAACCUUCG	**64.2**	–9.1	–6.2 ± 0.2	–53.5 ± 1.6	–159 ± 5
**IIb**	GAc^3^AGGGCAACCUUCG	**65.4**	–7.9	–6.4 ± 0.3	–53.8 ± 2.0	–159 ± 6
**IIc**	GAAc^7^GGGCAACCUUCG	**68.5**	–4.8	–6.5 ± 0.3	–51.5 ± 1.9	–151 ± 5
**IId**	GAc^7^AGGGCAACCUUCG	**69.8**	–3.5	–6.6 ± 0.4	–52.3 ± 3.3	–153 ± 10
**III**	GGUCGACC	**58.3**	-	–13.2 ± 0.9	–64.6 ± 8.6	–172 ± 26
**IIIa**	GGUCc^3^GACC	**44.8**	–13.5	–11.4 ± 0.5	–74.7 ± 6.4	–213 ± 20
**IIIb**	GGUCGc^3^ACC	**48.5**	–9.8	–11.7 ± 0.9	–68.1 ± 8.8	–189 ± 26
**IIIc**	GGUCc^7^GACC	**55.9**	–2.4	–13.4 ± 1.0	–70.6 ± 8.5	–192 ± 25
**IIId**	GGUCGc^7^ACC	**55.6**	–2.7	–13.0 ± 0.6	–67.0 ± 4.6	–181 ± 14
**III'**	GGCUAGCC	**60.7**	-	–14.5 ± 1.1	–72.3 ± 9.5	–194 ± 28
**III’a**	GGCUAc^3^GCC	**47.7**	–13.0	–11.8 ± 0.8	–70.9 ± 8.3	–199 ± 25
**III’b**	GGCUc^3^AGCC	**47.7**	–13.0	–10.7 ± 0.6	–56.5 ± 6.2	–154 ± 19
**III’c**	GGCUAc^7^GCC	**58.6**	–2.1	–13.7 ± 0.7	–68.8 ± 7.3	–185 ± 22
**III’d**	GGCUc^7^AGCC	**59.7**	–1.0	–14.2 ± 0.6	–71.8 ± 6.9	–193 ± 21

^a^Buffer: 10 mM Na_2_HPO_4_, 150 mM NaCl, pH 7.0. Δ*H* and Δ*S* values were obtained by van’t Hoff analysis or based on RNA concentration dependent measurements according to references 49 and 50.

^b^The estimated errors of UV-spectroscopically determined *T*_m_ values are ±0.3°C.

^c^Errors for Δ*H* and Δ*S* were determined from three (RNAs I, III, and III') and five (RNAs II) independent measurements; in general, errors arising from noninfinite cooperativity of two-state transitions and from the assumption of a temperature-independent enthalpy, are typically 10−15%. Additional error is introduced when free energies are extrapolated far from melting transitions; errors for ΔG are typically 3−5%.

To further elucidate the impact of 3- and 7-deazapurines on base pairing, we investigated the thermodynamic base pairing properties of 3- and 7-deazapurines in short palindromic RNAs. As mentioned above, the effects become intensified because the self-pairing results in two modified base pairs with regularly Watson-Crick–paired regions next to them that are below the required number of base pairs needed for successful double helix nucleation (45,46). Indeed, for c^3^G the reduction in both palindromic RNAs was dramatic, reflected in –13.5/–13.0°C reduced *T*_m_ values (**IIIa** and **III’a**), accounting for –6.8/–6.5°C destabilization per single modification which is more than twice of the destabilization that we observed for a single c^3^G-C base pair in the bimolecular 9 bp duplex **Ia**. The destabilizing nature was also very pronounced for c^3^A in both palindromic RNAs (–9.8°C in **IIIb** and –13.0°C in **III’b**). In contrast, only little destabilization was found for both 7-deazapurines in both palindromic RNAs (c^7^G: –2.4°C in **IIIc** and –2.1°C in **III’c**; c^7^A: –2.7°C in **IIId** and –1.0°C in **III’d**).

To the best of our knowledge, our data provides the first insights into thermal and thermodynamic stabilities of 3-deazaadenine and 3-deazaguanine containing RNA. Thus far, c^3^G was investigated only within the context of 2′-OCH_3_–RNA; c^3^G caused destabilization when paired to C (–6°C with complementary RNA; –9°C with complementary DNA in a Type I like sequence design) (47). Interestingly, when an acetyl group resided at the exocyclic amino group (*N*^2^-acetyl-3-deazaguanine) the native pairing strength was restored (48).

Furthermore, a single paper points at the strong destabilizing effect of 3-deazaguanine on DNA base pairing (36). In contrast, in several early studies, the slight destabilizing effect of 3-deazaadenine within DNA double helices has been recognized (31,32,35,36). 7-Deazaguanine and 7-deazaadenine modified DNAs have been investigated most intensively with respect to base pairing stabilities; generally, they act slightly destabilizing compared to their native counterparts (29,30,34–38).

### NMR spectroscopy of 3-deazapurine modified RNA

**Figure 3.**
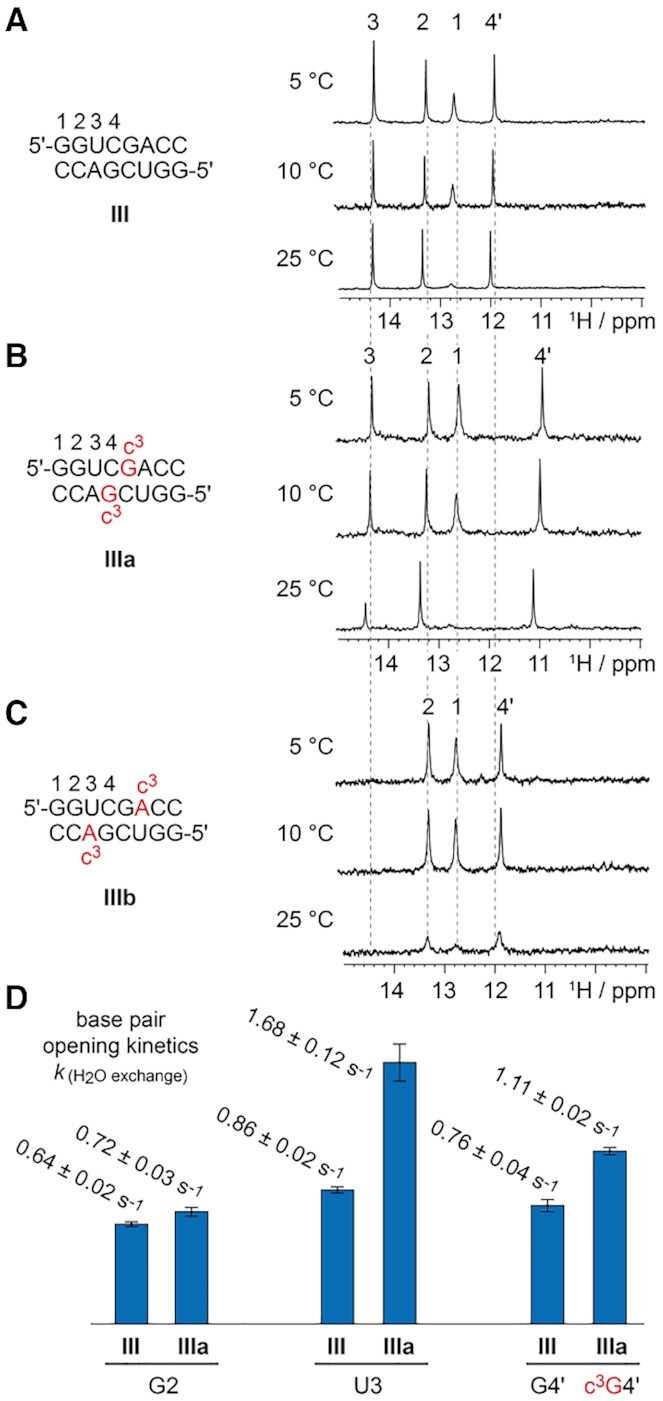
Comparative ^1^H NMR imino proton spectra of (**A**) unmodified, (**B**) c^3^G-, and (**C**) c^3^A-modified oligoribonucleotides; conditions: c_RNA_ = 0.1 mM, 15 mM Na[AsO_2_(CH_3_)_2_]•3H_2_O, 25 mM NaCl, 3 mM NaN_3_, in H_2_O/D_2_O 9/1, pH 6.5; (**D**) the graph illustrates the imino proton exchange rates determined by 1D CLEANEX-PM experiments (see Supporting Information for details).

The NMR resonances for the hydrogen-bonded protons of Watson–Crick base-pairs (‘imino protons’) directly reflect the double helical segments within folded RNA. The chemical shifts of these signals are characteristic for A-U (>14 ppm) and C-G base pairs (∼12–13 ppm), and the linewidths reflect proton exchange with the solvent. Clearly, they are very sensitive to modifications, in particular, if they concern the nucleobases. Figure 3A depicts ^1^H NMR spectra of the palindromic duplex 5′-GGUCGACC **III** that were collected as a function of temperature, over the range 5–25°C. The signal assignment was based on 2D ^1^H/^1^H-NOESY spectroscopy (Supplementary Figure S17). With increasing temperature, the signal of the base pair G1-C becomes broader and loses intensity, consistent with base pair fraying of the duplex termini; the internal base pairs remain and reflect stable duplex formation at ambient temperatures.

In comparison, the c^3^G imino proton resonance of the modified duplex **IIIa** was shifted highfield (Figure 3B), consistent with the increased p*K*_a_ of c^3^G (compared to G) of almost 3 p*K*_a_ units (Table 2). Interestingly, the neighboring U3-A base pair senses the perturbation caused by c^3^G, reflected in a decrease in intensity of the U3 imino proton signal at higher temperature. The enhanced exchange with the solvent indicates a dynamic hot spot of base pair opening at this particular position.

**Table 2.** p*K*_a_ values of nucleobases and deaza derivatives^a^

	A•H^+^ (N1)	c^3^A•H^+^ (N1)	c^7^A•H^+^ (N1)	U (N3)
p*K*_a_	3.7	6.8	5.3	9.2
	G (N1)	c^3^G (N1)^b^	c^7^G (N1)	C•H^+^ (N3)
p*K*_a_	9.5	12.3	10.3	4.1

^a^Values are summarized from references 7 and 51. Atom position of protonation/deprotonation are provided in brackets.

^b^For an independent NMR-based p*K*_a_ determination of c^3^G see Supplementary Figure S22.

Concerning the c^3^A-modified duplex **IIIb**, the thermal destabilization is reflected in the imino proton ^1^H NMR spectra as well (Figure 3C), with a tiny signal barrow at 5°C in the typical ppm region of A–U base pairs; no signal was observed at higher temperature. This stands for intensive proton exchange with the solvent of the uridine N3-H in the c^3^A–U base pair and is reminiscent to ^1^H NMR spectroscopy of 5F–U–A base pairs (52).

To investigate these effects in more detail, we quantified the water imino proton exchange kinetics of a c^3^G–C base pair containing duplex (**IIIa**) by the application of 1D Phase-Modulated CLEAN Chemical EXchange Spectroscopy (CLEANEX-PM) NMR experiments (Figure 3D, and Supplementary Figure S18) (53–55). Interestingly, the largest exchange rate *k*_(H2O ex.)_ of 1.68 s^−1^ was observed for the A-U base pair next to the c^3^G–C modification. Also for the c^3^G–C base pair itself the rate *k*_(H2O ex.)_ was increased compared to the unmodified duplex (1.11 s^−1^ versus 0.76 s^−1^), while the G–C base pair two positions away almost the same exchange rates *k*_(H2O ex.)_ for modified and unmodified duplexes were observed (0.72 s^−1^ versus 0.62 s^−1^). This is consistent with a local structural perturbation induced by the 3-deazapurine hotspot.

### Crystal structures of a c^3^A containing RNA

**Figure 4.**
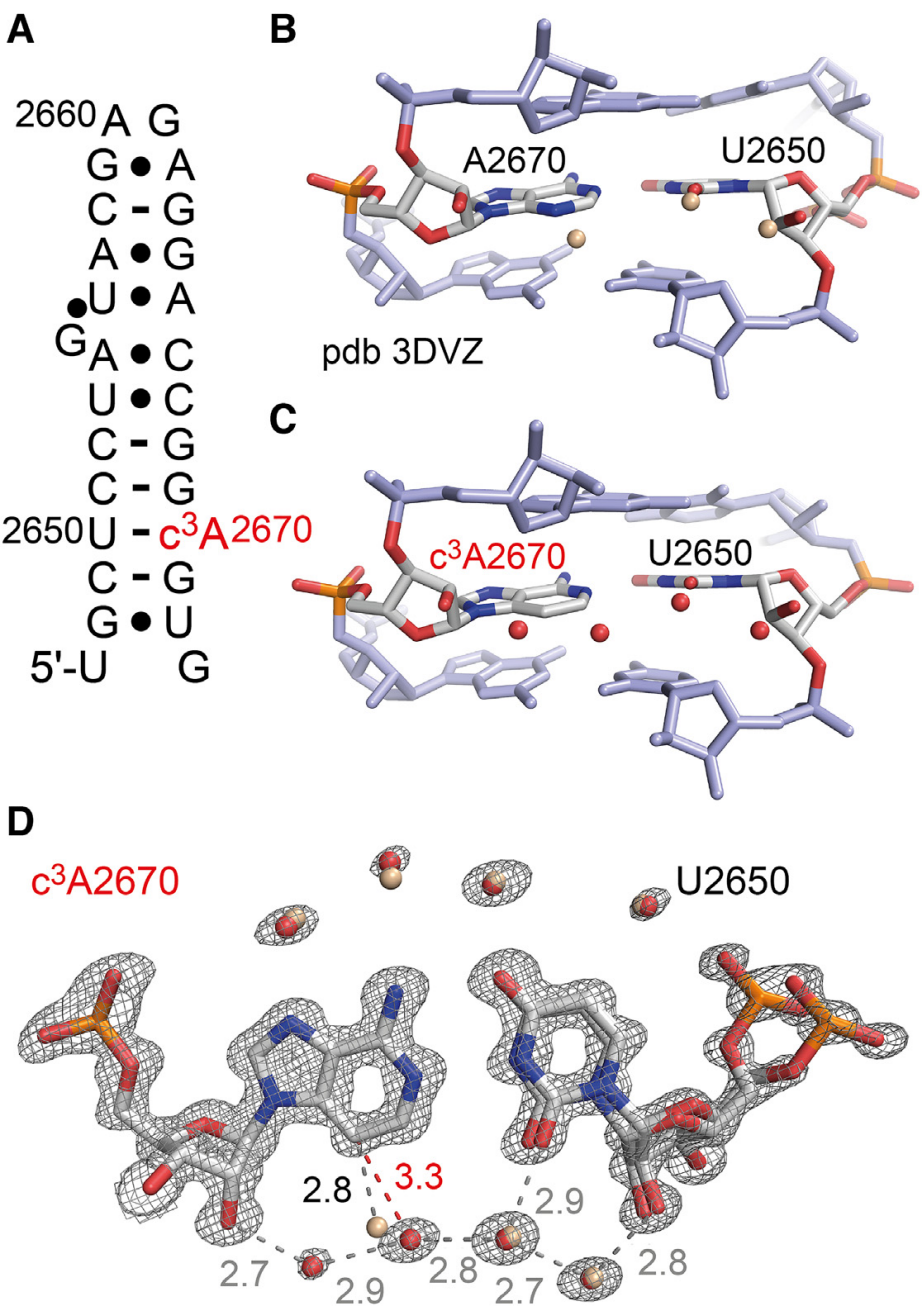
X-ray structure of 3-deazaadenosine modified RNA at atomic resolution. (**A**) Secondary structure of the *E. coli* Sarcin-ricin stem-loop (SRL) RNA used for crystallization. The c^3^A nucleotide is labeled in red. (**B**) Side view on the U2650/A2670 base pair in the unmodified duplex with ordered water molecules in the minor groove highlighted in beige color (PDB ID 3DVZ). (**C**) Side view on the U2650/c^3^A2670 base pair with ordered water molecules in the minor groove highlighted in red color (PDB ID 7L3R). (**D**) 2*F*_obs_ – *F*_calc_ electron density map contoured at 1.5 σ level showing the U2650/c^3^A2670 base pair. Water molecules are shown as red spheres (PDB ID 7L3R) and are superposed to the water molecules observed in the unmodified RNA (PDB ID 3DVZ). Numbers are distances in Angström (Å). We note that the alternative conformations of U2650 (and C2649, G2648) are also observed in the unmodified SRL RNA scaffold (PDB ID 3DVZ).

To further shed light on 3-deazapurine containing base pairs in RNA we put efforts into a high resolution X-ray crystallographic analysis. We utilized the 27 nt fragment of the *E. coli* 23S rRNA sarcin−ricin loop (SRL) which is a robust and well-behaved crystallization scaffold (56,57). For the incorporation of c^3^A, we deemed nucleotide A2670 appropriate, which forms a Watson−Crick base pair with C2650 in the regular A-form double helical region. Crystallization trials with c^3^A-modified SRL RNA indeed provided crystals that diffracted to subatomic resolution (Supplementary Table S3). X-ray structure determination showed that the c^3^A nucleobase is well-defined in the electron density maps for the c^3^A-modified RNA (Figure 4). Superimpositions of the c^3^A-modified RNA structure with the unmodified RNA showed a root-mean-square deviation (rmsd) of 0.12 Å (within the errors on coordinates of 0.13 Å). Overall, the structure revealed that the 3-deazanucleobase does not significantly affect the base pair geometry and the SRL RNA fold. However, what is different is the local hydration network in the minor groove of the c^3^A–U base pair (Figure 4A). While three ordered water molecules are found in the minor groove for the unmodified base pair, four are observed for the modified base pair. An obvious distinction is that the N3 atom of A2670 is hydrogen-bonded to one of the water molecules (2.8 Å distance) while for the c^3^A2670/U2650 base pair, a continuous hydrogen-bonded water chain spans from the 2′-OH of c^3^A2670 to the 2′-OH of U2650 without involving the c^3^A nucleobase; the closest water is in 3.3 Å-distance from its C3 atom (Figure 4D, Supplementary Figure S19). Although these structural differences in the hydration pattern appear minor in the static X-ray structure, they may contribute to increased base pair opening dynamics as reflected in the imino proton NMR spectrum of the c^3^A modified SRL RNA by significant signal broadening (Supplementary Figure S20). The destabilizing effect of the c^3^A modification in the SRL RNA hairpin is further reflected in the UV-melting profiles measured under diverse conditions (Supplementary Figure S21).

### Towards a rationale for the destabilization by c^3^G and c^3^A

For all four modifications—c^3^G, c^7^G and c^3^A, c^7^A—the base pair geometry and hydrogen bonding pattern to C and A respectively, are the same as encountered for the native Watson–Crick G–C and A–U base pairs. Therefore, other factors such as altered nucleobase dipole moments that can affect nucleobase stacking (58), or altered p*K*_a_ values of the modified nucleobases (7,59–61), as well as altered hydration of minor groove (3-deazapurines) and major groove (7-deazapurine) are of potential origin for these differences in thermal stabilities and thermodynamic parameters (30).

It has been proposed that the p*K*_a_ of a nucleobase is one significant factor that contributes to base-pairing strength (7,58–60). Table 2 summarizes the p*K*_a_ values of the nucleobases investigated in this study, and the following trend becomes obvious: The p*K*_a_ decreases in the order of A•H^+^ < c^7^A•H^+^ < c^3^A•H^+^, and the same is true for G < c^7^G < c^3^G. This trend is consistent with the notion that 7-deazapurines are less destabilizing than 3-deazapurines compared to A and G in standard Watson-Crick pairs in RNA double helices (Table 1). We furthermore note that c^3^A with a p*K*_a_ of 6.8 is partially protonated at physiological pH values around 7.0 which may affect base pairing in addition.

Another aspect that was documented earlier is that natural Watson–Crick base pairs A–U and C–G are characterized by a p*K*_a_ gap between the H-donor (U, G; N3-H, N1-H) and the H-acceptor (A, C; N1, N3) of Δp*K*_a_ 5.5 and 5.4, respectively, which appears optimal for efficient base pairing (7,58). This gap becomes significantly altered for isosteric c^7^A–U (Δp*K*_a_ 3.9), c^3^A–U (Δp*K*_a_ 2.4), c^7^G–C (Δp*K*_a_ 6.2) and c^3^G–C (Δp*K*_a_ 8.2) deazapurine base pairs.

Clearly, caution has to be taken because many more factors in addition to the p*K*_a_ of the heterocycles (as listed above) contribute to macroscopically observed double helix stabilities. This might be the reason why concepts for predicting hydrogen-bond strengths from acid−base molecular properties (e.g. proton affinity/p*K*_a_ equalization, p*K*_a_ slide rule, etc.) cannot be directly applied to nucleic acid base pairing strengths, although being promising for an integrated theoretical and experimental approach to solve this long-standing problem in the future (62).

Finally, we point at the hydration of RNA grooves which is distinct between 3-deazapurines (c^3^G and c^3^A) affecting the minor groove, and 7-deazapurines (c^7^G and c^7^A) affecting the major groove. In particular, the first solved crystal structure of a c^3^A modified RNA and the comparison to the unmodified counterpart reveal that the characteristic water molecule that always docks on the purine N3 atom and often spans further to the ribose 2′-OH in native RNA double helices (63,64), is lacking in the 3-deaza-modified counterparts. The altered hydration pattern is a clear structural distinction that should be taken into account when searching for the concurrent causes of the decrease in stability.

### Role of deazapurines in RNA atomic mutagenesis studies

Deazapurine modified RNAs have been frequently applied in biochemical mutagenesis studies of ribozymes to verify or falsify mechanistic proposals for the chemical reactions they catalyze (65,66). Several examples are found in the literature where atomic mutagenesis experiments led to an in-depth understanding of the chemical mechanism, revealing the functionally crucial imino groups of purines that participate in general acid-base catalysis of small nucleolytic ribozymes for phosphodiester cleavage (14–16). For instance, this concerns the twister ribozyme where proton transfer from the (protonated) N3 of the conserved adenine (A6) at the cleavage site to the 5′-O leaving group had been postulated to contribute to reaction catalysis (i.e. δ-catalysis; for explanation of α, ß, γ, δ-catalysis see reference 67). Indeed, replacement of this adenine by c^3^A or c^1^c^3^A rendered the twister ribozyme inactive (14,67). A follow-up NMR spectroscopic analysis revealed a p*K*_a_ shift of the very adenine N3 from 3.7 to 5.1 (14). As a second example, the pistol ribozyme is pointed out. At first, distance analysis and interpretation of the three-dimensional structure of this ribozyme implicated the N3 of a conserved purine (A32 or G32, respectively) as a possible candidate for general acid–base catalysis, being involved in leaving group stabilization (δ-catalysis). However, upon replacement of this purine by c^3^A, the ribozyme retained full activity. Later, it was found that the 2′-OH of this purine nucleoside plays a crucial role, and not the N3 (15).

Additionally, deaza-nucleobase substitutions have identified crucial metal binding sites that are responsible for structuring of riboswitch aptamers and ribozyme active sites (13,67), as well as have disclosed key coordination sites of hydrated Mg^2+^ ions that critically participate in proton transfer to release the 5′-*O* leaving group in the course of phosphodiester cleavage (15,16,68,69). Recent prominent examples refer to the NAD^+^ class-I riboswitch (68) and the pistol ribozyme (15,16). In pistol RNA, a highly conserved guanine (G33) interacts with a Mg^2+^ cation through innersphere coordination with its N7 atom. Replacement of this guanine by c^7^G renders the ribozyme inactive (15). Together with distance analysis of pistol ribozyme crystal structures, this suggests that a water molecule of the hydrated Mg^2+^ ion assists in stabilizing the 5′-O leaving group during the course of the reaction (15,16). In the NAD^+^ class-I riboswitch, a highly conserved adenine (A10) exhibits N7-innersphere coordination to a Mg^2+^ cation that additionally coordinates to the oxygen atom of a sequence-distant phosphate backbone unit and thus molds a critical part of the binding pocket. Replacement of this adenine by c^7^A abolishes binding to NAD^+^ (68).

With respect to the ribosome, atomic mutagenesis involving 3-deazaadenine in the ribosomal peptidyl transferase center (PTC) demonstrated that an initial suspect (N3 of A2451) did not actively participate in proton transfer to catalyze peptide bond formation, but instead, the 2′-OH of the very adenosine assists in positioning of the substrate (19; see also reference 17,18,20,70).

For atomic mutagenesis experiments using deazanucleobases, a profound knowledge of the intrinsic properties of these heterocycles in an RNA context is imperative to avoid misinterpretations. Functional assays to evaluate ribozyme or riboswitch activity in response to a specific purine-deazapurine replacement are usually designed to impair a potential H-bond or innersphere coordination to a divalent metal ion, as suggested by the observed distances in the three-dimensional structure. Provided such an interaction is crucial for activity, it can thus be easily identified and the mechanistic interpretation becomes straightforward. Importantly, in all of the examples mentioned above, the purine/deazapurine finds itself in an exposed and unpaired position. Clearly, in the light of our present study, functional assays that would involve atomic mutagenesis with 3-deazanucleobases in double helical regions would encounter severe limitations because of thermodynamic destabilization of the stem containing the modification. This aspect is, however, less critical for atomic mutagenesis using 7-deazanucleobases which do not or only minimally destabilize double helices.

## CONCLUDING REMARKS

In this study, we synthesized and comprehensively analyzed the properties of 3-deazaguanine and 3-deazaadenine modified RNA. When these nucleobases are embedded in a double helical environment we found that they can decrease the stability of base pairing. Interestingly, the effects were much more pronounced for 3-deazapurines compared to the isomeric 7-deazapurines. Moreover, destabilization is more severe in short stems interfering with double helix nucleation, while a more modest decrease is observed if located in the center of an extended double helix. We furthermore found that the imino proton exchange for deazapurine-modified Watson Crick base pairs is significantly higher compared to their native counterparts and simultaneously induces enhanced opening of the very neighbor base pair. These perturbations in local structural dynamics provide a rationale for the observed reduced base pairing strength. Additionally, we solved the X-ray structure of a c^3^A modified RNA to visualize the hydration pattern of the minor groove. The structure points out that the hydrophobic minor grove face of c^3^A impairs hydration because the water molecule that is usually hydrogen bonded to the N3 atom becomes extruded from the 3-deaza-modified base pair. Taken together, our comparative study sheds light on Watson–Crick base pairing of 3- and 7-deazapurines and thus represents a first important step towards a comprehensive understanding of the intrinsic properties of these RNA modifications that are critical for their proper application in atomic mutagenesis experiments of biologically relevant RNA.

## DATA AVAILABILITY

Atomic coordinates and structure factors for the reported crystal structures have been deposited with the Protein Data bank under accession numbers 7L3R.

## Supplementary Material

gkab256_Supplemental_File
