# Supplementary material for: Impact of 3-deazapurine nucleobases on RNA properties
Source: Nucleic Acids Res. 2021 Apr 15;49(8):4281–93. doi: 10.1093/nar/gkab256 (PMC8096147; doi:10.1093/nar/gkab256)
Supplement: gkab256_Supplemental_File [file gkab256_supplemental_file.pdf]

Supporting Information

Impact of 3-deazapurine nucleobases on RNA properties

R. Bereiter, M. Himmelstoß, E. Renard, E. Mairhofer, M. Egger, K. Breuker, C. Kreutz, E. Ennifar, R. Micura

Contents

Supporting procedures and NMR spectra of organic compounds

N[2]-Tfa 2'-*O*-Tbs 3-deazaguanosine phosphoramidite **8**	2
N[2]-Tfa 2'-*O*-Cem 3-deazaguanosine phosphoramidite **9**	27
3-Deazaadenosine phosphoramidite **17**	45

Supporting Tables

Supporting Table 1	57
Supporting Table 2	58
Supporting Table 3	59

Supporting Figures

Supporting Figure 1	60
Supporting Figure 2	61
Supporting Figure 3	62
Supporting Figure 4	63
Supporting Figure 5	64
Supporting Figure 6	65
Supporting Figure 7	66
Supporting Figure 8	67
Supporting Figure 9	68
Supporting Figure 10	69
Supporting Figure 11	70
Supporting Figure 12	71
Supporting Figure 13	72
Supporting Figure 14	73
Supporting Figure 15	74
Supporting Figure 16	75
Supporting Figure 17	76
Supporting Figure 18	77
Supporting Figure 19	78
Supporting Figure 20	79
Supporting Figure 21	80
Supporting Figure 22	81

References 82

N^2-Tfa 2'-*O*-Tbs 3-deazaguanosine phosphoramidite 8

6-Benzoxy-2-bromo-9-(*β*-D-ribofuranosyl)-3-deazapurine (1a)

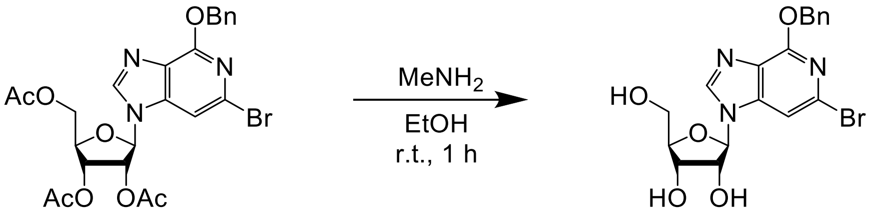

1 **1a**

Compound **1** [1] (8.60 g, 15.30 mmol) was dissolved in a solution of 33 wt% methylamine in ethanol (37.80 g, 50 ml, 401.64 mmol) and stirred at room temperature for one hour. After complete consumption of the starting material, the mixture was diluted with CH_2Cl_2 (100 ml) and the solvents were evaporated. The crude product was purified by column chromatography on SiO_2 (0 % to 15 % MeOH in CH_2Cl_2). Yield: 6.60 g of compound **1a** as a white solid (99 %). TLC: (CH_2Cl_2 / MeOH, 9:1): R_f = 0.44. ESI-MS (m/z): $[M+H]^+$ calcd.: 436.0458; found: 436.0489. ^{1}H-NMR: (400 MHz, DMSO-d_6, 25 °C): δ = 3.60-3.70 (m, 2H, ***H(a)***-C(5') & ***H(b)***-C(5')); 3.98-4.00 (q, 1H, ***H***-C(4')); 4.08-4.12 (m, 1H, ***H***-C(3')); 4.24-4.29 (q, 1H, ***H***-C(2')); 5.19 (t, 1H, ***H***O-C(5')); 5.22 (d, 1H, J=4.75 Hz, ***H***O-C(3')); 5.50 (m, 3H, C$\boldsymbol{H_2}$-(benzyl) & ***H***O-C(2')); 5.85 (d, 1H, J=6.00 Hz), ***H***-C(1')); 7.33-7.42 (m, 3H, ***H***-C(arom.)); 7.52 (m, 2H, ***H***-C(arom.); 7.80 (s, 1H, ***H***-C(3)); 8.47 (s, 1H, ***H***-C(8)) ppm. ^{13}C-NMR: (100 MHz, DMSO-d_6, 25 °C): δ = 61.01 (***C***(5')); 68.81 (-***C***H_2-(benzyl)); 70.01 (***C***(3')); 74.26 (***C***(2')); 85.98 (***C***(4')); 89.18 (***C***(1')); 105.79 (***C***(3)); 128.00-128.43 (m, ***C***-(arom.(benzyl)); 136.58; 141.48; 142.94 (***C***(8)); 153.76 ppm.

^{1}H-NMR (400 MHz, DMSO-d_6, 25 °C) of compound **1a**

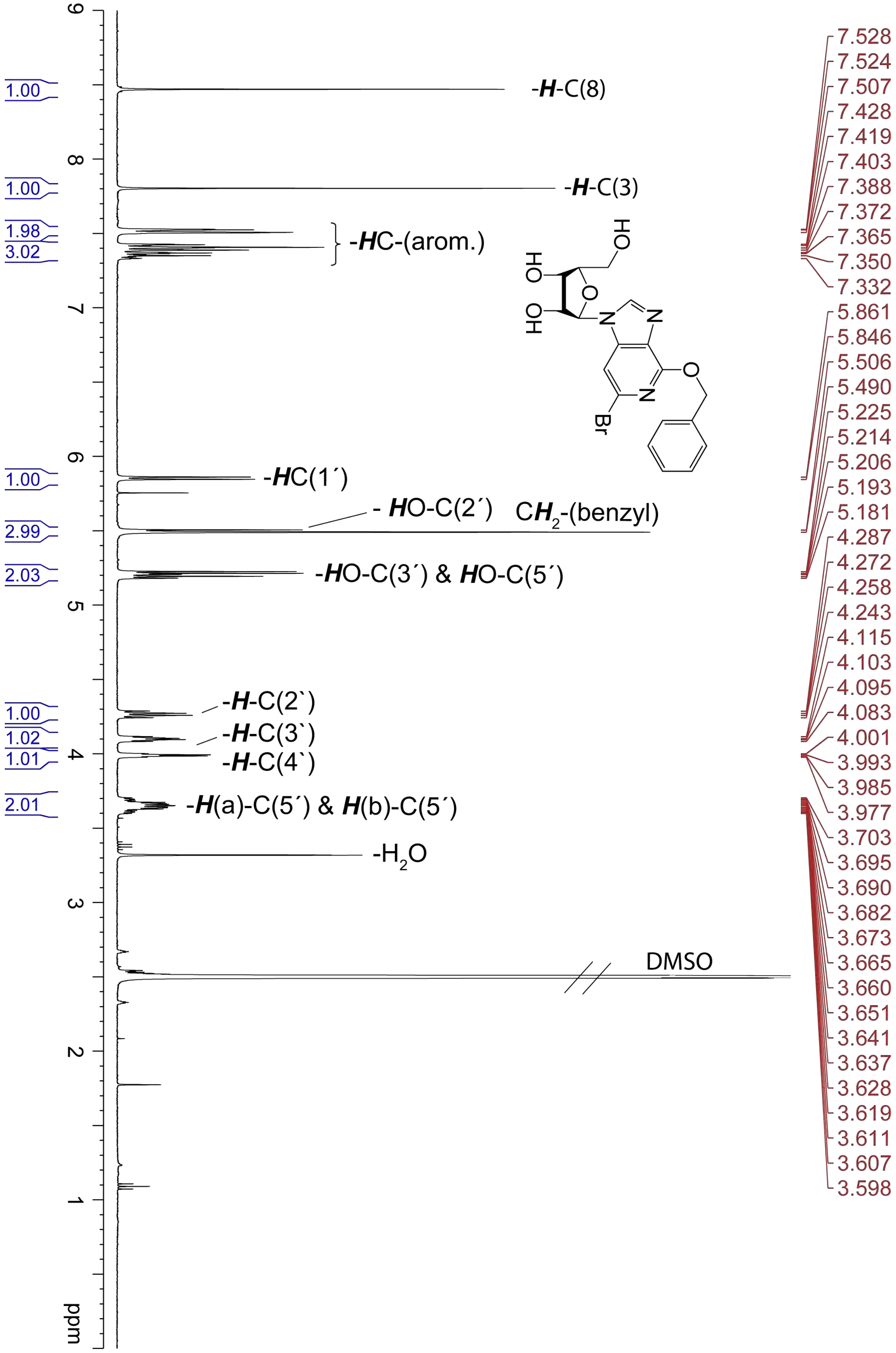

^{13}C-NMR (100 MHz, DMSO-d_6, 25 °C) of compound **1a**

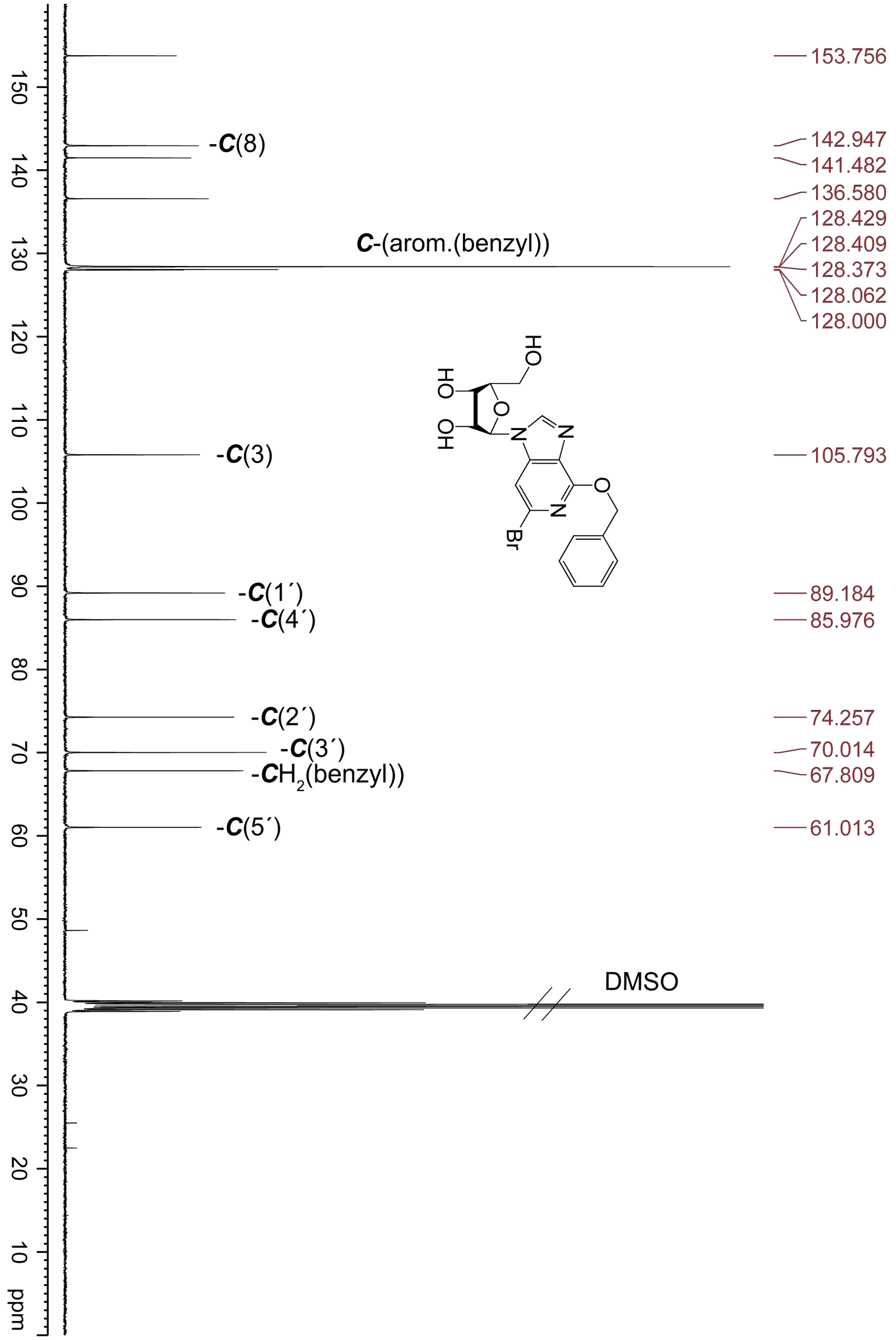

6-Benzoxy-2-bromo-9-[2',3',5'-*O*-tris(*tert.*-butyldimethylsilyl)-*β*-D-ribofuranosyl]-3-deazapurine (2)

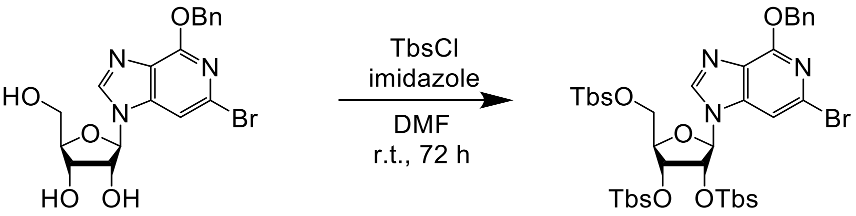

1a **2**

Compound **1a** (6.60 g, 15.13 mmol), *tert*-butyldimethylsilyl chloride (TbsCl, 13.68 g, 90.77 mmol) and imidazole (7.21 g, 105.90 mmol) were dissolved in DMF and stirred for 72 hours at room temperature. Then, the solvent was evaporated and the resulting oily residue was diluted with ethyl acetate (200 ml), washed three times with brine (50 ml), and dried over $MgSO_4$. The crude product was purified by column chromatography on SiO_2 (0 % to 10 % ethyl acetate in cyclohexane). Yield: 10.6 g of compound **2** as a white foam (90 %). TLC: (CH_2Cl_2/MeOH, 9.95/0.05): R_f = 0.79. HR-ESI-MS (m/z): $[M+H]^+$ calcd.: 778.3052; found: 778.3054. ^{1}H-NMR: (400 MHz, $CDCl_3$, 25 °C): δ = -0.55 (s, 3H, Si-C***H₃***); -0.12 (s, 3H, Si-C***H₃***); 0.12 (d, 6H, J=6.93 Hz, 2xSi-C***H₃***); 0.18 (d, 6H, J= 2.00 Hz, 2xSi-C***H₃***); 0.77 (s, 9H, Si-C(C***H₃***)₃)); 0.96 (d, 18H, J=6.86 Hz, 2xSi-C(C***H₃***)₃)); 3.81-3.95 (qxd, 2H, ***H(a)***-C(5') & ***H(b)***-C(5')); 4.13 (m, 1H, ***H***-C(4')); 4.20 (d, 1H, J=5.36 Hz, ***H***-C(3')); 4.32 (q, 1H, ***H***-C(2')); 5.58 (s, 2H, C***H₂***-(benzyl)); 5.76 (d, 1H, J=6.63 Hz, ***H***-C(1')); 7.26-7.38 (m, 3H, ***H***-C(arom.)); 7.55-7.57 (m, 2H, ***H***-C(arom.); 7.36 (s, 1H, ***H***-C(3)); 8.07 (s, 1H, ***H***-C(8)) ppm. ^{13}C-NMR: (100 MHz, $CDCl_3$, 25 °C): δ = (-5.55)-(-4.37) ***C***H_3-Si-***C***H_3(TBDMS); 17.88-18.71 -Si-C(***C***H_3)$_3$ (TBDMS); 25.80-26.33 -Si-***C***(CH_3)$_3$(TBDMS); 63.47 (***C***(5')); 68.71 (-***C***H_2-(benzyl)); 73.06 (***C***(3')); 76.62 (***C***(2')); 87.46 (***C***(4')); 89.08 (***C***(1')); 105.15 (***C***(3)); 128.04-129.93 (m, ***C***-(arom.benzyl)); 136.67; 141.50; 141.55 (***C***(8)); 154.65 (***C***(6)) ppm.

^{1}H-NMR (400 MHz, $CDCl_3$, 25 °C) of compound **2**

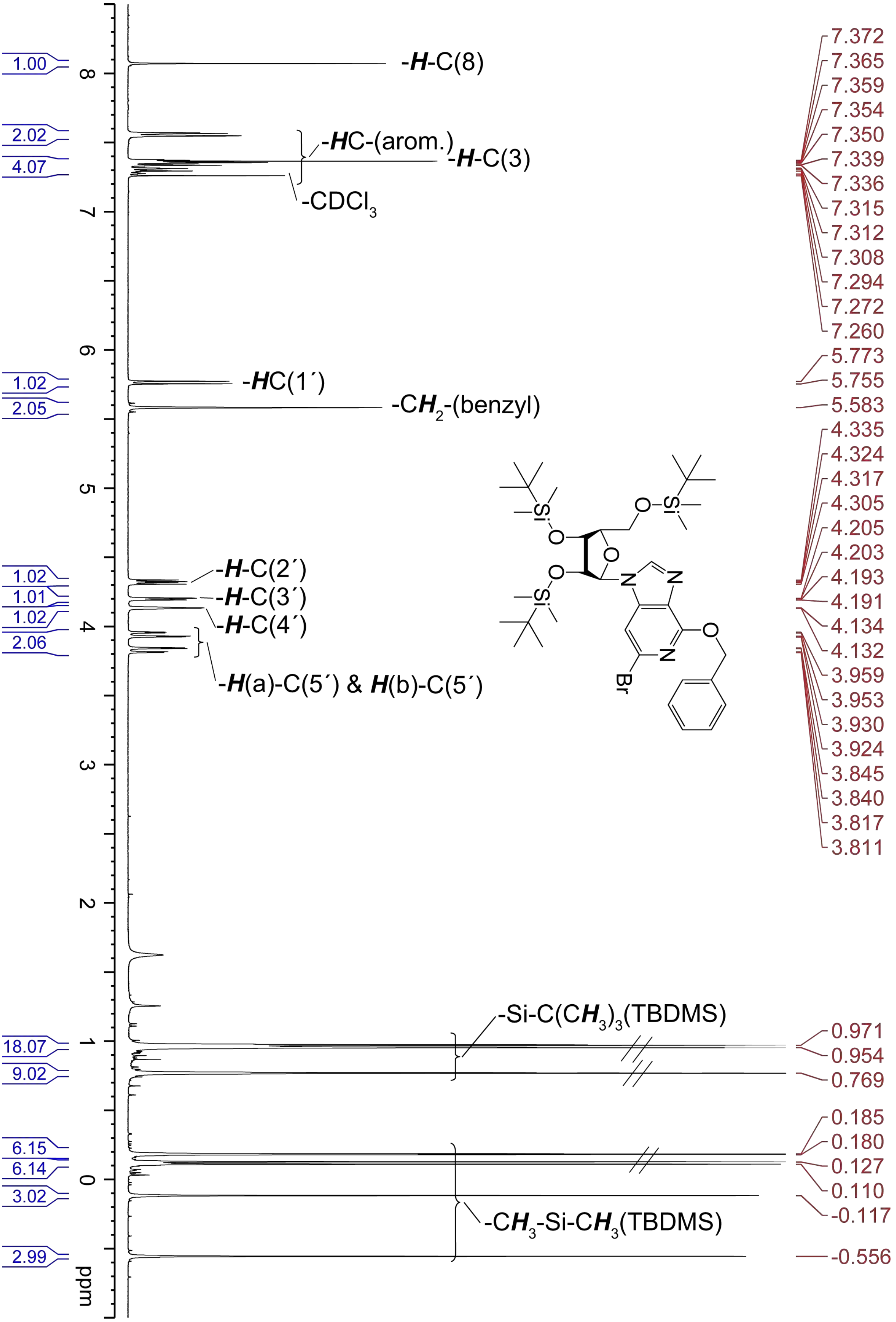

^{13}C-NMR (100 MHz, $CDCl_3$, 25 °C) of compound **2**

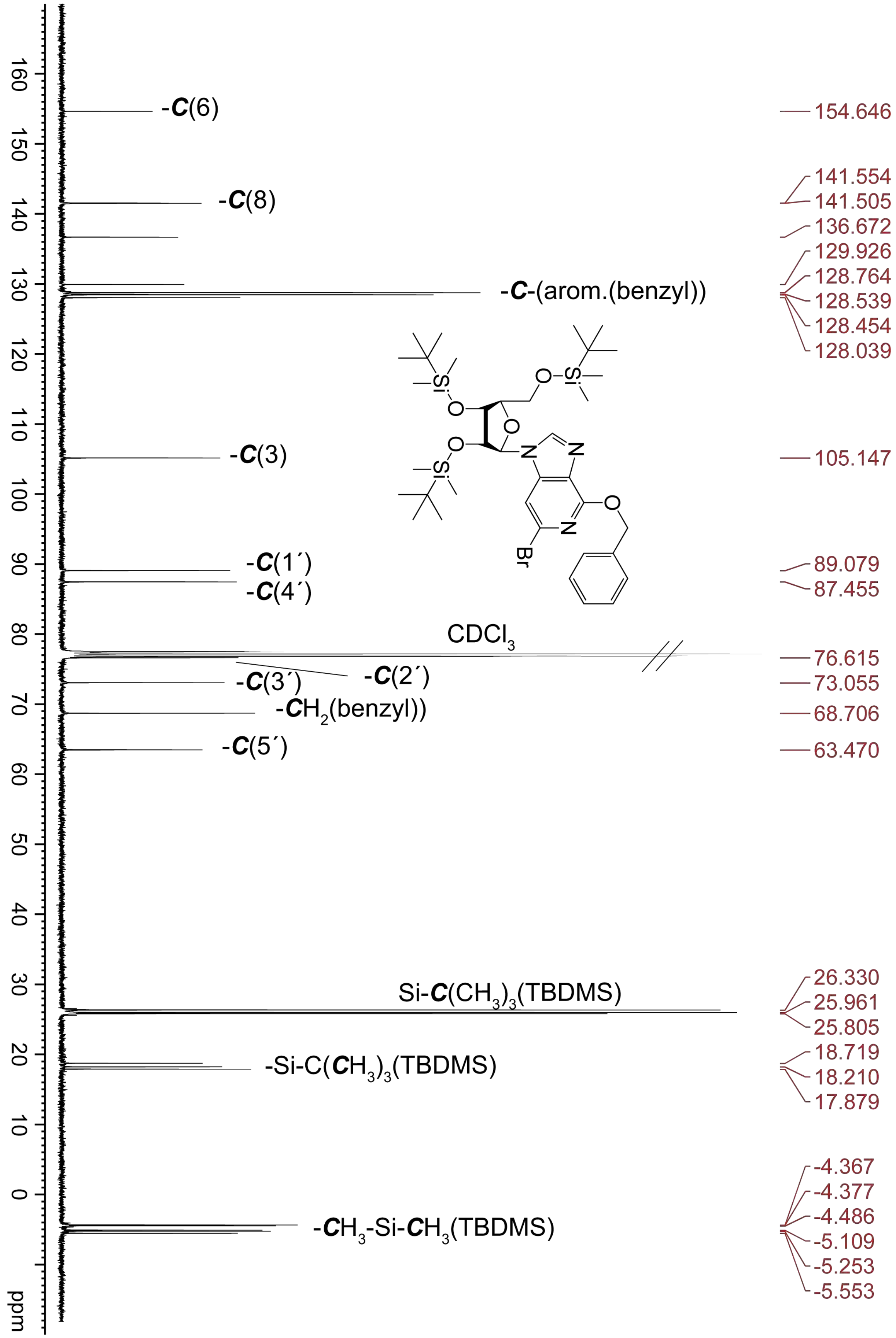

O^6-Benzyl-N^2-trifluoroacetyl-2',3',5'-O-tris(*tert.*-butyldimethylsilyl)-3-deazaguanosine (3)

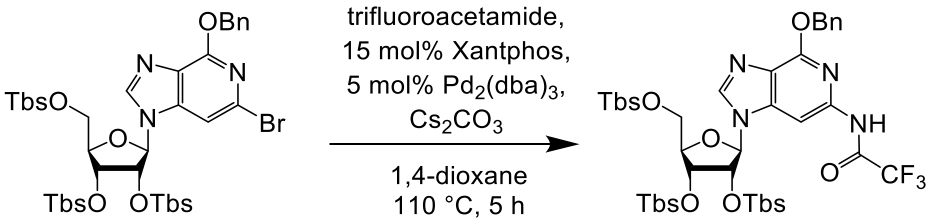

2 **3**

Compound **2** (3.26 g, 4.18 mmol) was dissolved in dry 1,4-dioxane (60 ml) and the resulting solution was degassed with three freeze pump thaw cycles. Meanwhile, the required equipment (100 ml two necked round bottom flask and a mini reflux condenser) was heated up with a hot air gun under high vacuum, backfilled with argon and charged with trifluoroacetamide (567.63 mg, 5.02 mmol), Xantphos (363.20 mg, 0.63 mmol), Cs_2CO_3 (1.91 g, 5.86 mmol) and $Pd_2(dba)_3$ (191.60 mg 0.21 mmol). A rubber septum was applied and the apparatus was evacuated and backfilled with argon two times. Then, compound **2** in degassed 1,4-dioxane was added via a syringe. The yellow mixture was stirred at 110 °C under argon atmosphere for five hours. The resulting suspension was diluted with CH_2Cl_2 (20 ml), filtered over celite and washed with CH_2Cl_2 until UV active substances were no longer visible. The solvent was evaporated and the crude product was purified by column chromatography on SiO_2 (0 % to 15 % ethyl acetate in cyclohexane). Yield: 3.04 g of compound **3** as a white foam (90 %). TLC: (cyclohexane / ethyl acetate, 8/2): R_f = 0.46. HR-ESI-MS (m/z): $[M+H]^+$ calcd.: 811.3879; found: 811.3885. ^{1}H-NMR: (400 MHz, $CDCl_3$, 25 °C): δ = -0.45 (s, 3H, Si-C***H₃***); -0.09 (s, 3H, Si-C***H₃***); 0.13 - 0.16 (m, 12H, Si-C***H₃***); 0.77 (s, 9H, Si-C(C***H₃***)₃)); 0.95 (d, 18H, J=1.75 Hz, 2xSi-C(C***H₃***)₃)); 3.84 – 3.97 (qxd, 2H, ***H(a)***-C(5') & ***H(b)***-C(5')); 4.15 (m, 1H, ***H***-C(4')); 4.23 (d, 1H, J=5.36 Hz, ***H***-C(3')); 4.35 (m, 1H, ***H***-C(2')); 5.55 (s, 2H, C***H₂***-(benzyl)); 5.86 (d, 1H, J=7.35 Hz, ***H***-C(1')); 7.30-7.38 (m, 3H, ***H***-C(arom.)); 7.50 (m, 2H, ***H***-C(arom.); 7.97 (s, 1H, ***H***-C(3)); 8.20 (s, 1H, ***H***-C(8)); 8.36 (s, 1H, -N***H***(Tfa)) ppm. ^{13}C-NMR: (100 MHz, $CDCl_3$, 25 °C): δ = (-5.31)-(-4.31) ***C***H_3-Si-***C***H_3(TBDMS); 17.90-18.62 -Si-C(***C***H_3)$_3$ (TBDMS); 25.78-27.05 -Si-***C***(CH_3)$_3$(TBDMS); 63.43 (***C***(5')); 68.29 (-***C***H_2-(benzyl)); 72.98 (***C***(3')); 76.73 (***C***(2')); 86.80 (***C***(4')); 89.07 (***C***(1')); 91.40 (***C***(3)); 127.23-128.55 (m, ***C***-(arom.benzyl)); 136.81; 140.54; 141.49; 141.78 (***C***(8)); 153.815; 154.19; 154.28 (***C***(6)); 154.57 ppm.

^{1}H-NMR (400 MHz, $CDCl_3$, 25 °C) of compound **3**

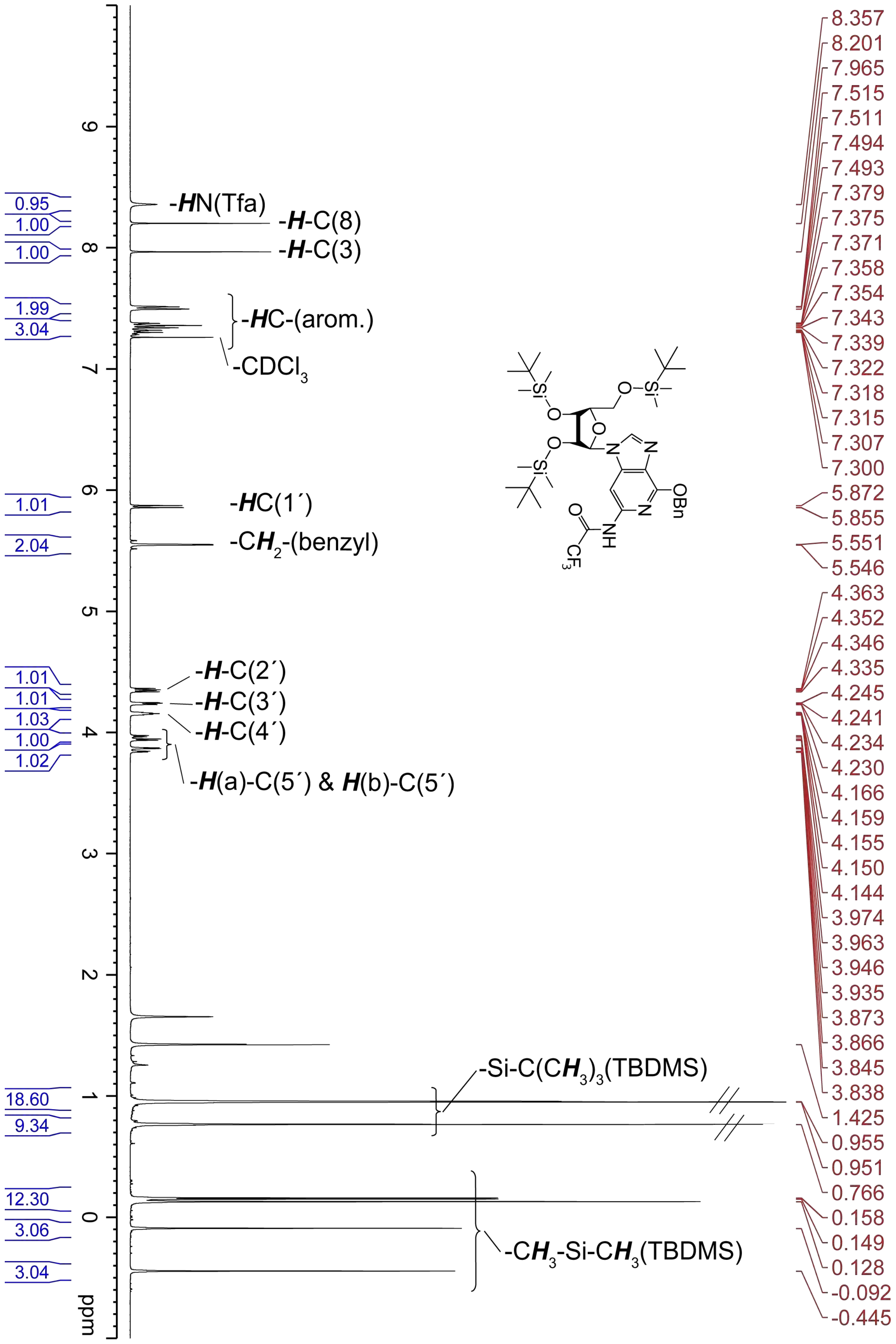

^{13}C-NMR (100 MHz, $CDCl_3$, 25 °C) of compound **3**

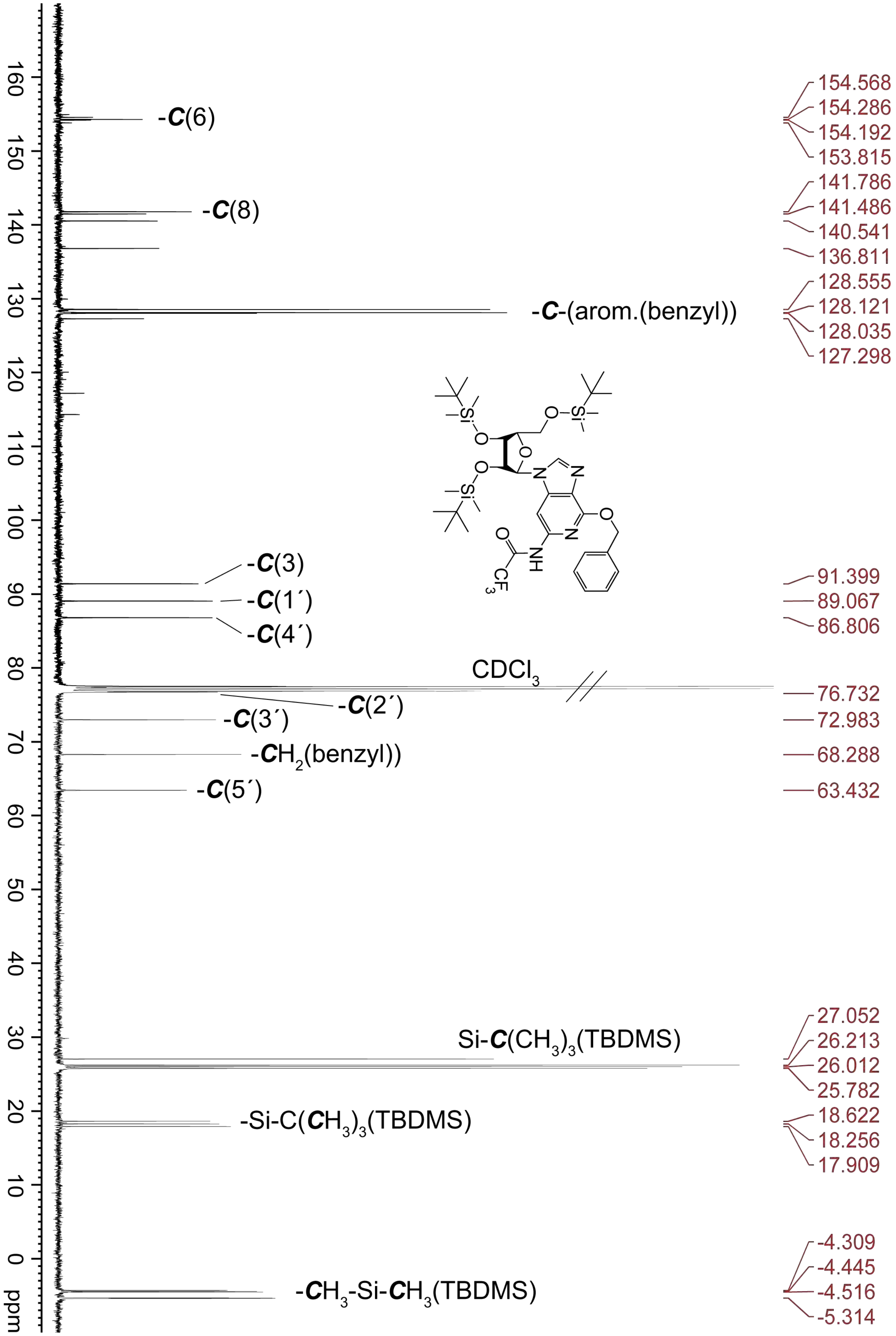

O^6-Benzyl-N^2-trifluoroacetyl-3-deazaguanosine (3)

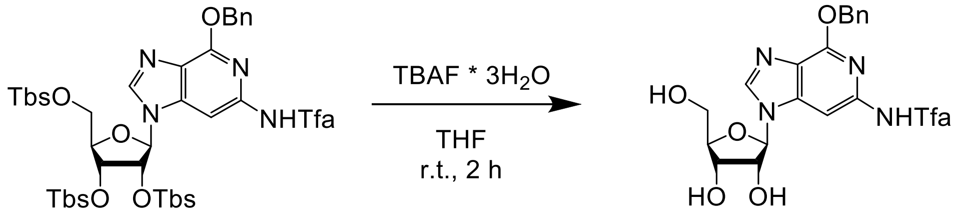

3 **4**

Compound **3** (5.30 g, 6.53 mmol) was dissolved in tetrahydrofuran (50 ml) and tetra-*n*-butylammonium fluoride trihydrate (TBAF * 3 H_2O, 8.45 g, 26.79 mmol) was added. After two hours of stirring, the ribose was completely deprotected and the solvent was evaporated. The crude product was purified by column chromatography on SiO_2 (0 % to 10 % MeOH in CH_2Cl_2). Yield: 2.65 g of compound **4** as a white solid (87 %). TLC: (CH_2Cl_2/MeOH, 9/1): R_f = 0.46. HR-ESI-MS (m/z): $[M+H]^+$ calcd.: 469.1285; found: 469.1304. ^{1}H-NMR: (400 MHz, DMSO-d_6, 25 °C): δ = 3.60-3.66 (m, 2H, ***H(a)***-C(5') & ***H(b)***-C(5')); 3.98 (q, 1H, ***H***-C(4')); 4.09 (m, 1H, ***H***-C(3')); 4.34 (q, 1H, ***H***-C(2')); 5.07 (t, 1H, ***H***O-C(5')); 5.29 (d, 1H, J=4.92 Hz, ***H***O-C(3')); 5.54 (d, 1H, J=6.50 Hz, ***H***O-C(2')); 5.59 (s, 2H, -C***H_2***-(benzyl)); 5.82 (d, 1H, J=6.24 Hz, ***H***-C(1')); 7.35-7.43 (m, 3H, ***H***-C(arom.)); 7.54 (m, 2H, ***H***-C(arom.)); 7.89 (s, 1H, ***H***-C(3)); 8.48 (s, 1H, ***H***-C(8)); 11.68 (s, 1H, ***H***N-(TFA)) ppm. ^{13}C-NMR: (100 MHz, DMSO-d_6, 25 °C): δ = 61.72 (***C***(5')); 67.62 (-***C***H_2-(benzyl)); 70.77 (***C***(3')); 74.50 (***C***(2')); 86.32 (***C***(4')); 89.06 (***C***(1')); 93.44 (***C***(3)); 126.82-128.91 (m, ***C***-(arom.(benzyl)); 137.38; 141.18; 141.69; 143.07 (***C***(8)); 153.67 (***C***(6)); 154.94; 155.31 ppm.

^{1}H-NMR (400 MHz, DMSO-d_6, 25 °C) of compound **4**

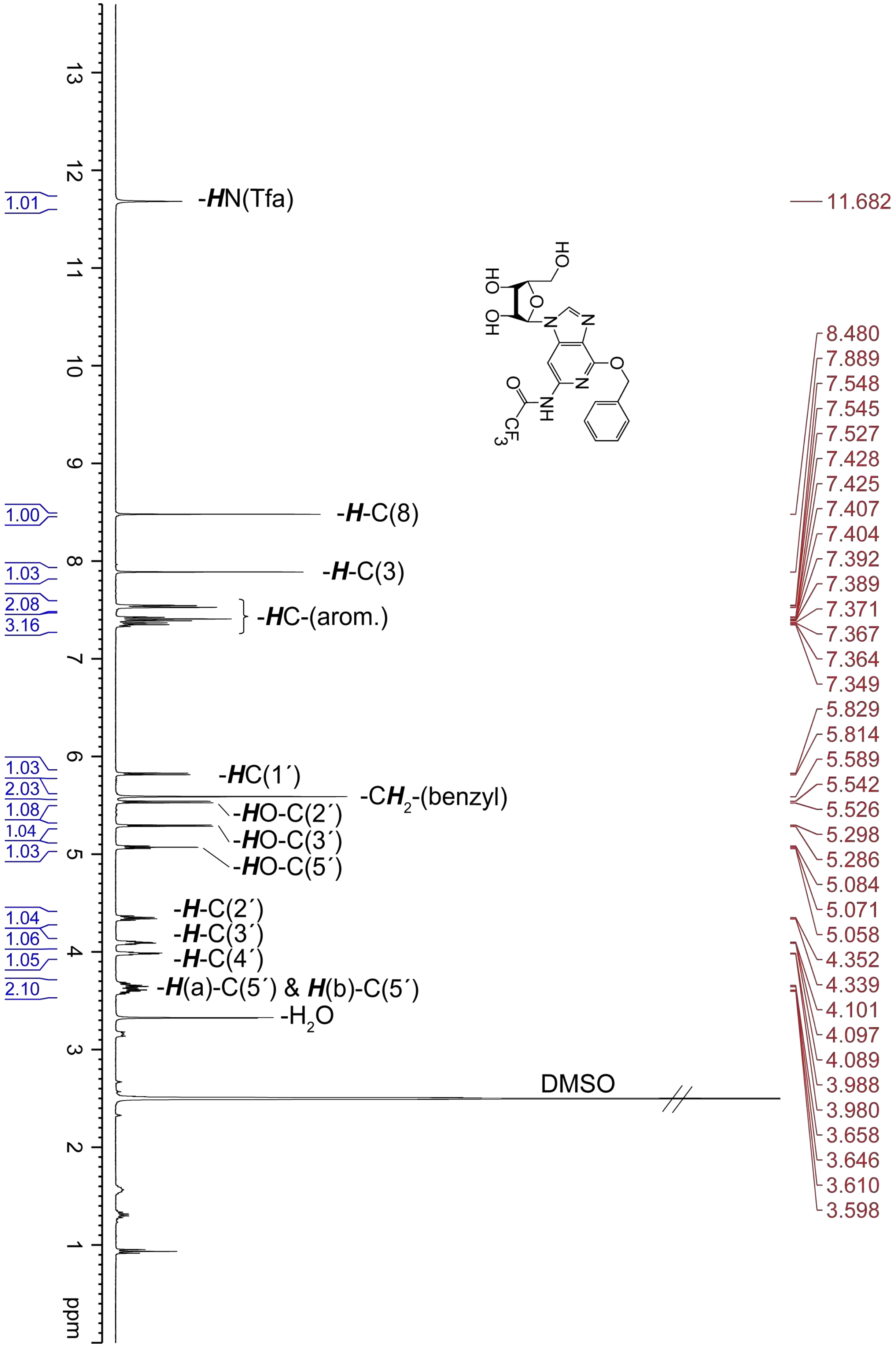

^{13}C-NMR (100 MHz, DMSO-d_6, 25 °C) of compound **4**

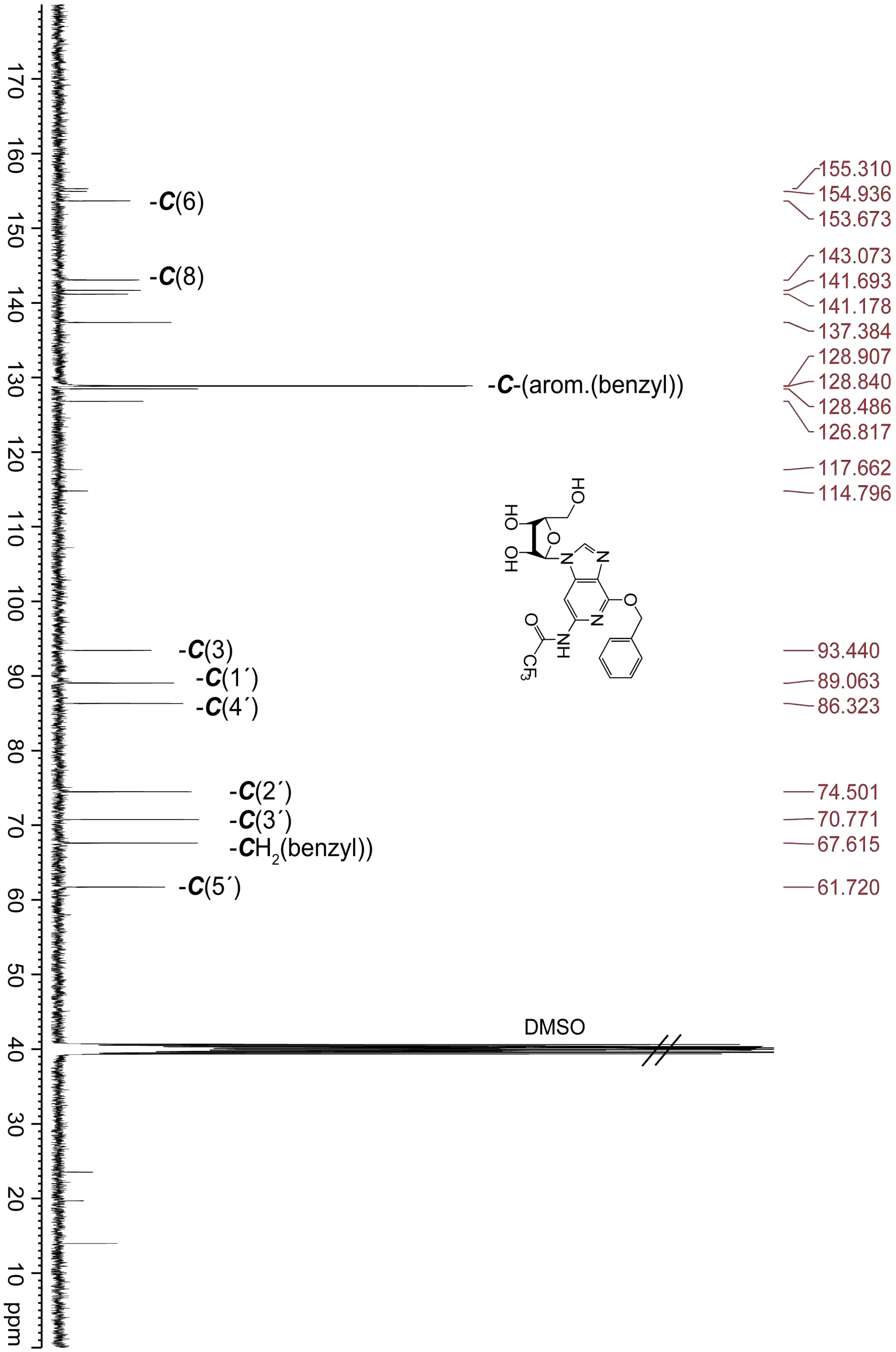

O^6-Benzyl-5'-O-(4,4'-dimethoxytrityl)-N^2-trifluoroacetyl-3-deazaguanosine (5)

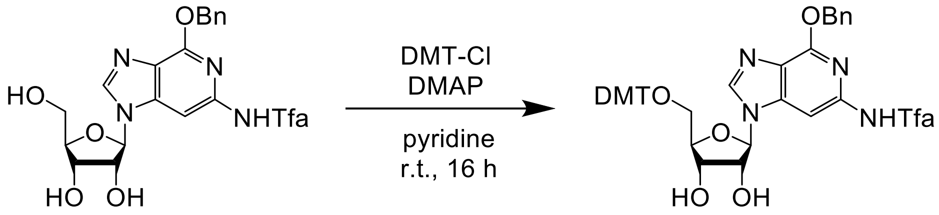

4 **5**

Compound **4** (1.07 g, 2.28 mmol) and 4-(dimethylamino)pyridine (DMAP, 14.0 mg, 0.11 mmol) were co-evaporated three times with dry pyridine and finally dissolved in dry pyridine (19.5 ml). Afterwards, dry 4,4'-dimethoxytrityl chloride (DMT-Cl, 890.14 mg, 2.63 mmol) was added in two portions over a period of one hour. Each time when 4,4'-dimethoxytrityl chloride was added, the reaction mixture was heated up to 35 °C for one minute. Stirring was continued over night for 16 hours at room temperature and the reaction was finally quenched by the addition of 1 ml methanol. The solvent was evaporated and the resulting residue was diluted with CH_2Cl_2 (100 ml) and transferred into a separating funnel. The dissolved crude product was washed first with 5 % citric acid (50 ml), followed by saturated $NaHCO_3$ solution and finally with brine (50 ml). The resulting organic layer was dried over Na_2SO_4, evaporated to dryness and purified by column chromatography on SiO_2 (0 % to 4 % MeOH in CH_2Cl_2). Yield: 1.30 g of compound **5** as a white foam (74 %). TLC: (CH_2Cl_2/MeOH, 95/5): R_f = 0.54. HR-ESI-MS (m/z): $[M+H]^+$ calcd.: 771.2592 ; found: 771.2603. ^{1}H-NMR: (400 MHz, DMSO-d_6, 25 °C): δ = 3.22 (m, 2H, ***H(a)***-C(5') & ***H(b)***-C(5')); 3.70 (d, J= 1.36 Hz, 6H, 2x -O-C***H₃***); 4.09 - 4.17 (m, 2H, ***H***-C(4') & ***H***-C(3')); 4.50 (t, 1H, ***H***-C(2')); 5.30 (d, 1H, J=5.82 Hz, ***H***O-C(3')); 5.59 (d, 2H, J=3.40 Hz, C***H₂***-(benzyl)); 5.70 (d, 1H, J=5.88 Hz, ***H***O-C(2')); 5.91 (d, 1H, J=4.52 Hz, ***H***-C(1')); 6.76-7.56 (m, 18H, ***H***-C(arom.)); 7.93 (s, 1H, ***H***-C(3)); 8.39 (s, 1H, ***H***-C(8)); 11.72 (s, 1H, ***H***N-(TFA)) ppm. ^{13}C-NMR: (75 MHz, DMSO-d_6, 25 °C): δ = 54.92 (2 x -O-***C***H_3); 63.42 (***C***(5')); 67.20 (-***C***H_2-(benzyl)); 70.29 (***C***(3')); 73.55 (***C***(2')); 83.40 (***C***(4')); 85.47 ($\boldsymbol{C}_{(quat.)}$trityl); 89.08 (***C***(1')); 92.79 (***C***(3)); 113.06 - 129.36 (***C***-(arom.)); 135.37; 135.47; 136.11; 136.85; 140.97; 141.43; 141.86 (***C***(8)); 144.77; 149.60; 153.20; 158.00 ppm.

^{1}H-NMR (400 MHz, DMSO-d_6, 25 °C) of compound **5**

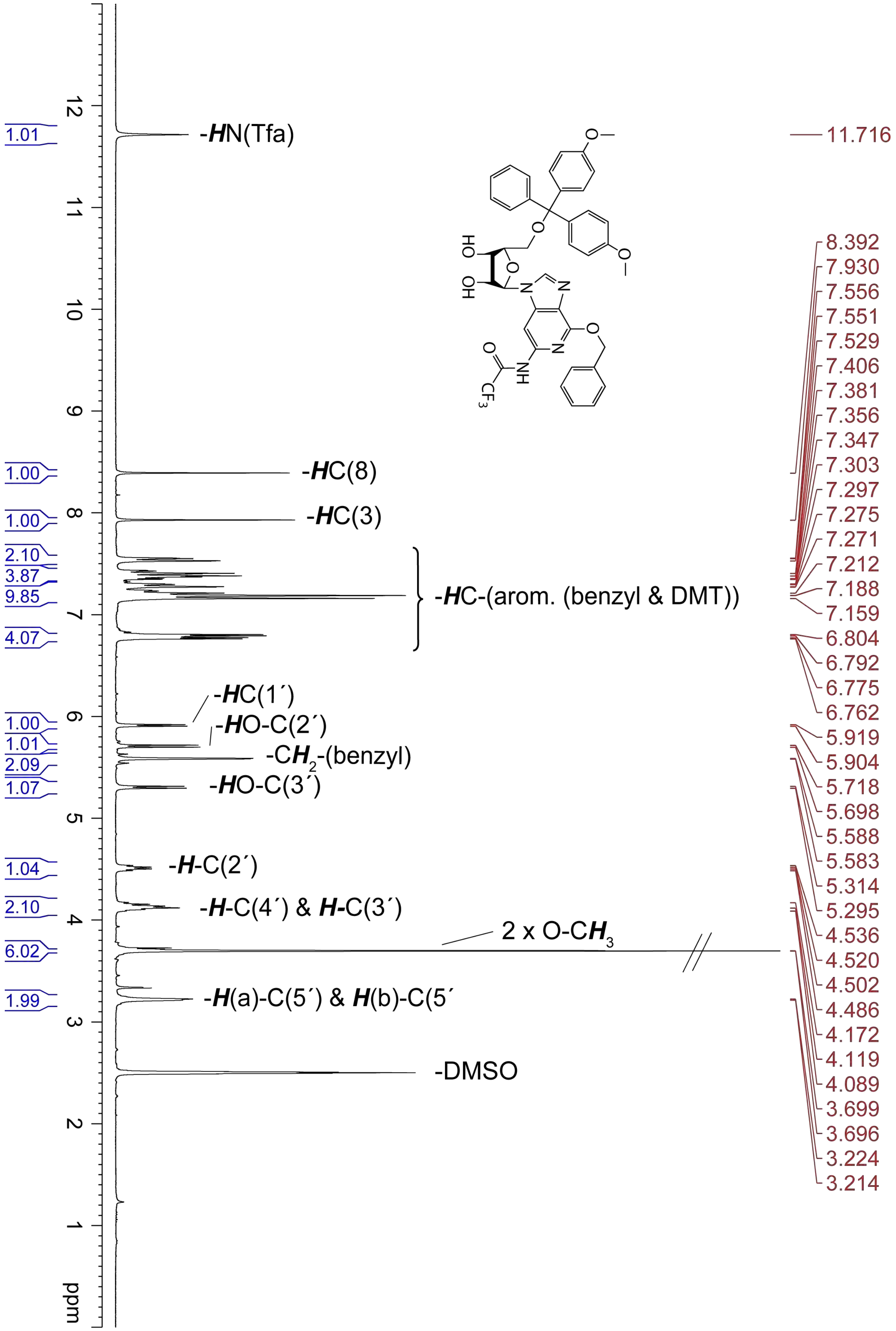

^{13}C-NMR (100 MHz, DMSO-d_6, 25 °C) of compound **5**

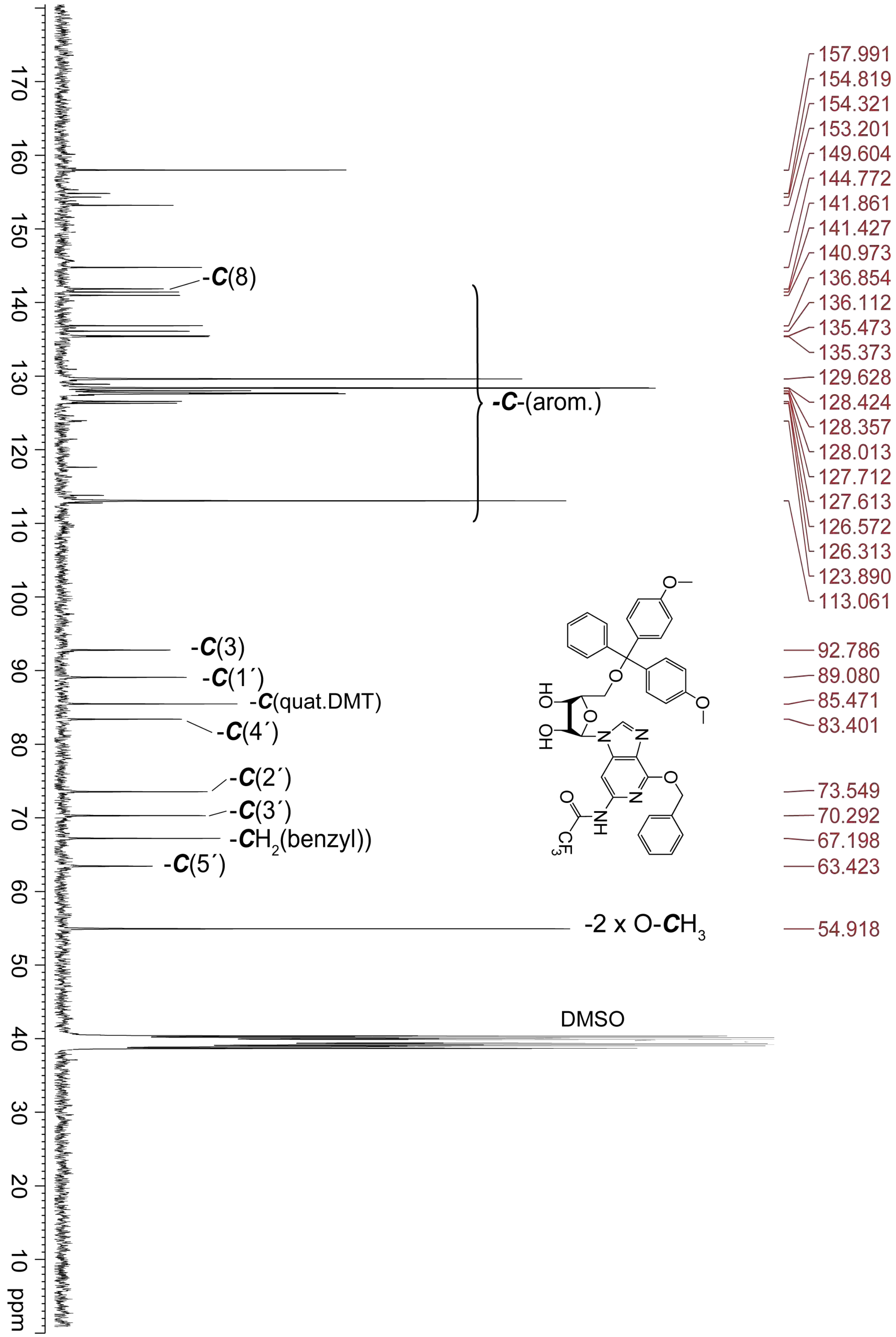

O^6-Benzyl-2'-*O*-*tert*-butyldimethylsilyl-5'-*O*-(4,4'-dimethoxytrityl)-N^2-trifluoroacetyl-3-deazaguanosine (6)

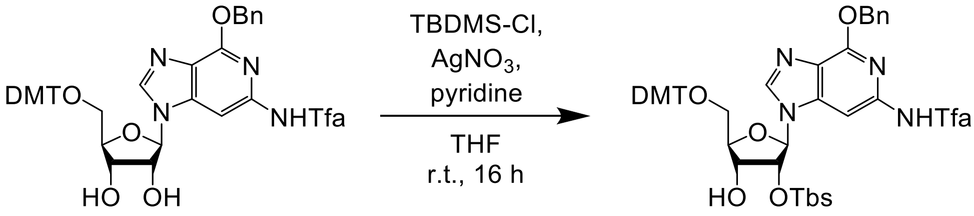

5 **6**

Compound **5** (952.00 mg, 1.24 mmol) and dry pyridine (224.71 mg, 228.83 µl, 2.84 mmol) were dissolved in dry tetrahydrofuran (16 ml). Then, silver nitrate ($AgNO_3$, 398.65 mg, 2.35 mmol) was added and the mixture was stirred under light exclusion for 30 minutes. Subsequently, *tert*-butyl-dimethylsilyl chloride (TBDMS-Cl, 353.71 mg, 2.35 mmol) was added and stirring was continued for 16 hours. After an almost complete consumption of the starting material into a mixture of the 2'- and 3'-regioisomer, the precipitated silver chloride was filtered of and washed with dichloromethane. The solvents were evaporated under reduced pressure and the residue was dissolved in dichloromethane and washed with half saturated bicarbonate solution and brine. The organic layer was dried over Na_2SO_4 and concentrated. The 2'- and 3'-regioisomers were separated by column chromatography on SiO_2 (10 % to 20 % ethylacetate in toluene). TLC: (toluene/ethylacetate, 8/2): R_f = 0.46. Fractions containing both regioisomers were equilibrated in dichloromethane/methanol/triethylamine 8/1/1 and isolated to per description. Yield: After two rounds of equilibration, 423 mg of compound **6** were isolated as a white foam (39 %). HR-ESI-MS (m/z): $[M+H]^+$ calcd.: 885.3456 ; found: 885.3518. $[M+K]^+$ calcd.: 924.3093 ; found: 924.3044 ^{1}H-NMR: (400 MHz, DMSO-d_6, 25 °C): δ = -0.16 (s, 3H, Si-C***H₃***); -0.05 (s, 3H, Si-C***H₃***); 0.76 (s, 9H, Si-C(C***H₃***)₃); 3.28 (m, 2H, ***H(a)***-C(5') & ***H(b)***-C(5')); 3.70 (d, J=0.72Hz, 6H, 2x -O-C***H₃***); 4.11 - 4.14 (m, 2H, ***H***-C(4') & ***H***-C(3')); 4.54 (t, 1H, ***H***-C(2')); 5.25 (d, 1H, J=6.04 Hz, ***H***O-C(3')); 5.58 (s, 2H, C***H₂***-(benzyl)); 5.93 (d, 1H, ***H***-C(1')); 6.80-7.55 (m, 18H, ***H***-C(arom.)); 7.89 (s, 1H, ***H***-C(3)); 8.38 (s, 1H, ***H***-C(8)); 11.70 (s, 1H, ***H***N-(TFA)) ppm. ^{13}C-NMR: (100 MHz, DMSO-d_6, 25 °C): δ = -5.44 (Si-***C***H₃); -4.89 (Si-***C***H₃); 17.78 (Si-***C***(CH₃)₃); 25.53 (Si-C(***C***H₃)₃); 54.94 (2 x -O-***C***H₃); 63.32 (***C***(5')); 67.25 (-***C***H₂-(benzyl)); 70.17 (***C***(3')); 75.74 (***C***(2')); 83.84 (***C***(4')); 85.59 ($C_{(quat.)}$trityl); 88.93 (***C***(1')); 92.59 (***C***(3)); 113.10 - 129.67 (***C***-(arom.)); 135.27; 135.41; 136.79; 136.79; 141.02; 141.20 (***C***(8)); 141.72; 144.77; 153.22; 154.34; 158.20 ppm.

^{1}H-NMR (400 MHz, DMSO-d_6, 25 °C) of compound **6**

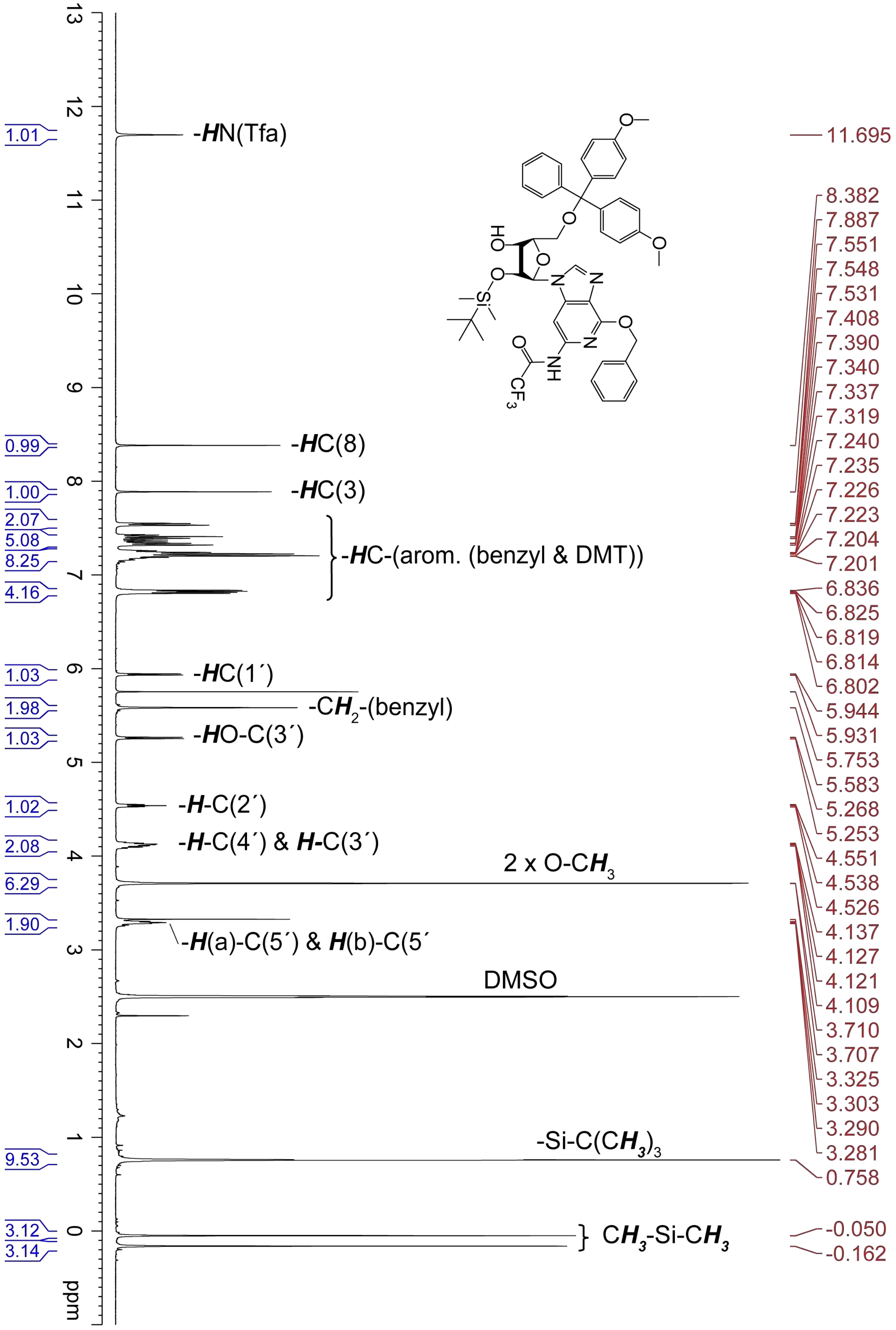

^{13}C-NMR (100 MHz, DMSO-d_6, 25 °C) of compound **6**

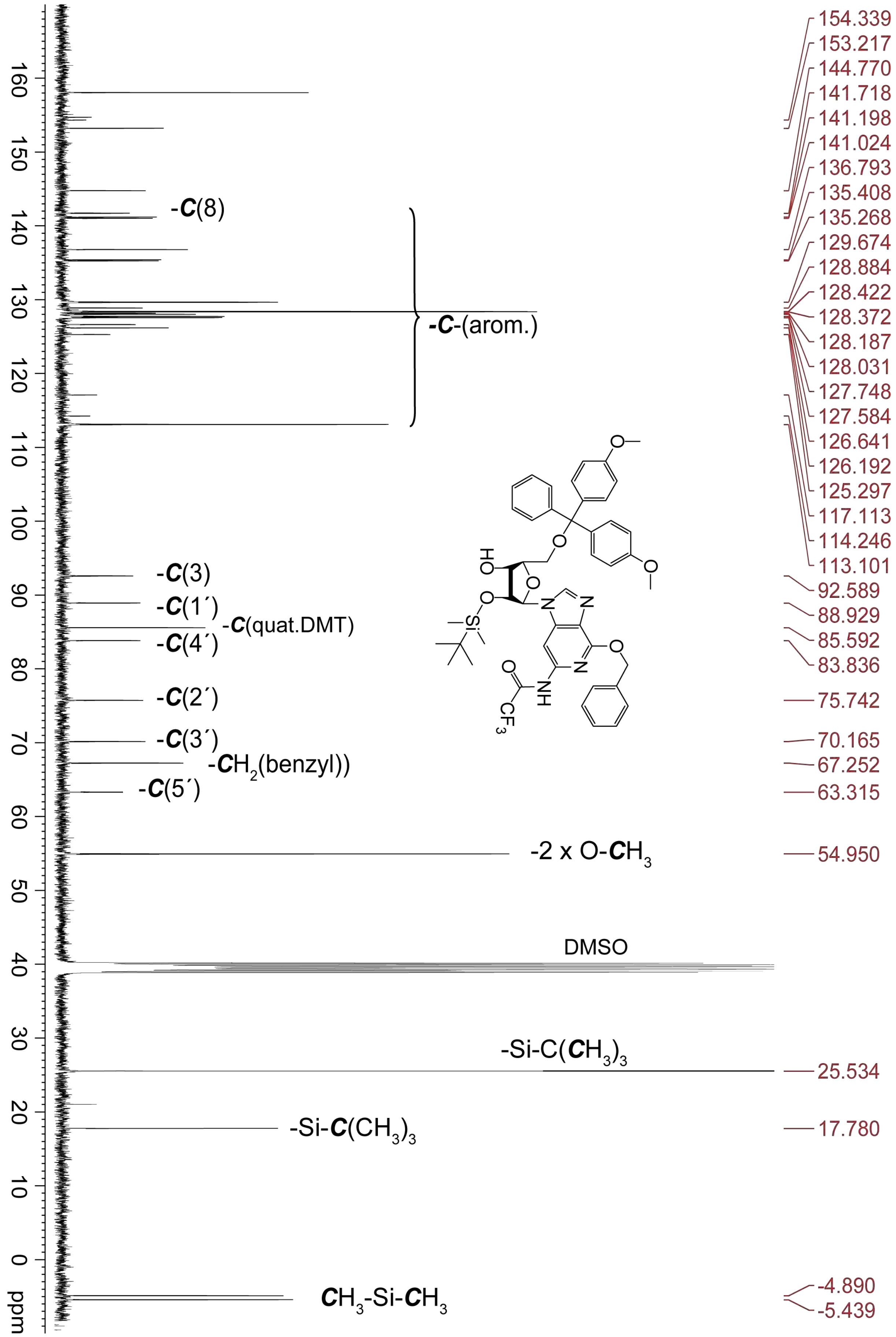

^{1}H-^{1}H-COSY-NMR (400 MHz, DMSO-d_6, 25 °C) of compound **6**

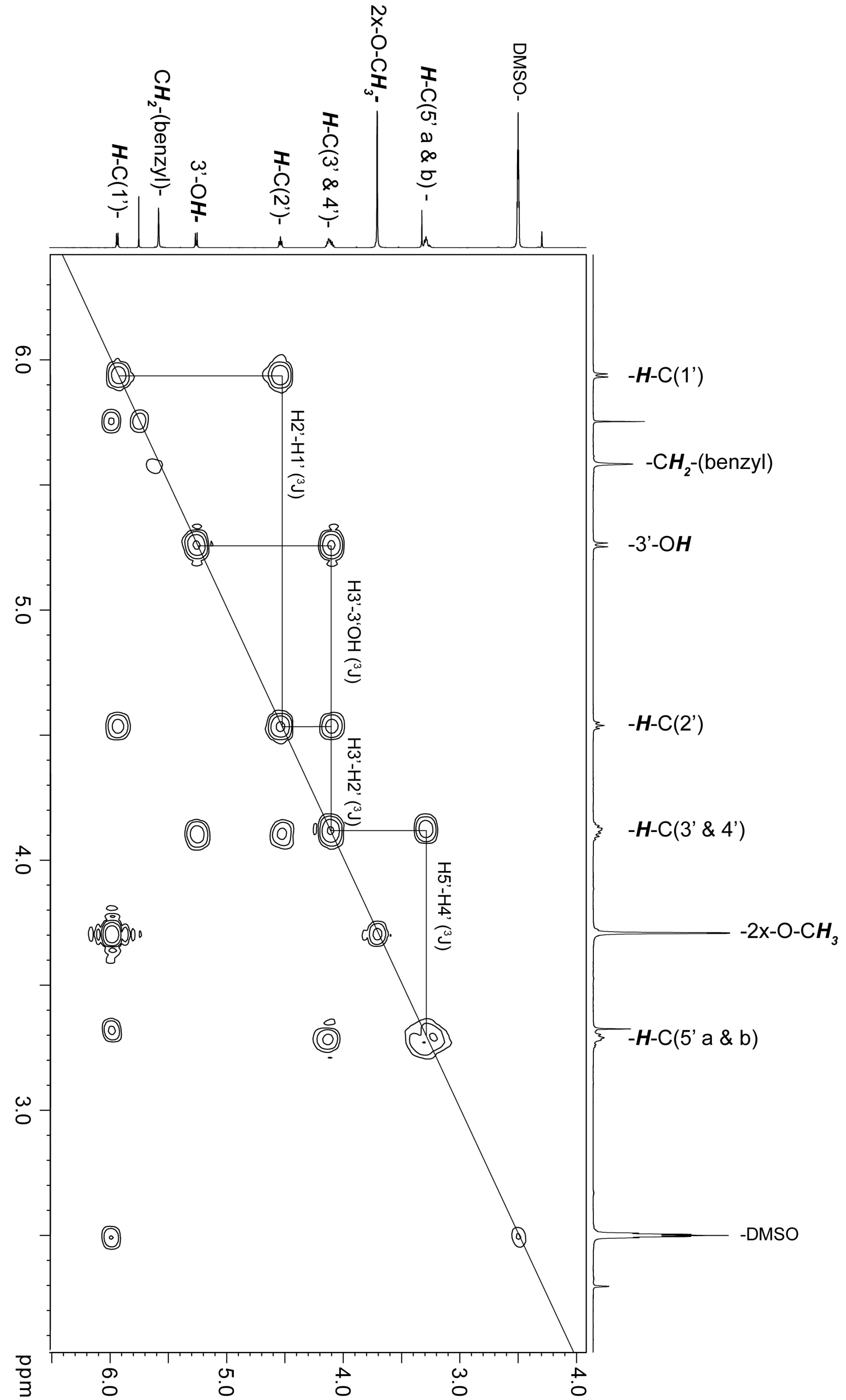

2'-*O*-*tert*-Butyldimethylsilyl-5'-*O*-(4,4'-dimethoxytrityl)-*N*²-trifluoroacetyl-3-deazaguanosine (7)

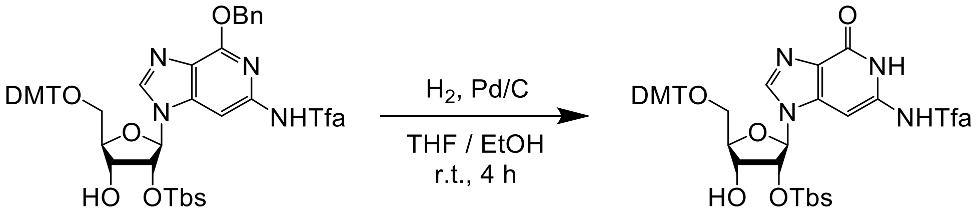

6 **7**

Compound **6** (380 mg, 0.43 mmol) was dissolved in a mixture of anhydrous tetrahydrofuran and dry ethanol (7 ml, 4:1). Subsequently, palladium on charcoal (167.81 mg, 0.14 mmol, 10 % Pd) was added and hydrogen gas (balloon via syringe) was bubbled through a rubber septum to the reaction mixture for 10 minutes followed by stirring under hydrogen atmosphere for four hours. Reaction control by thin layer chromatography showed complete consumption of the starting material. The resulting dark suspension was filtered over celite to remove the catalyst and the filtrate was evaporated to dryness. The crude product was purified by column chromatography on SiO_2 (0 % to 5 % MeOH in CH_2Cl_2). Yield: 270.00 mg of compound **7** as a white foam (79 %). TLC: (CH_2Cl_2/MeOH, 95/5): R_f = 0.39. HR-ESI-MS (m/z): $[M+Na]^+$ calcd.: 834.2624 ; found: 834.2608. ^{1}H-NMR: (400 MHz, DMSO-d_6, 25 °C): δ = -0.14 (s, 3H, Si-C***H₃***); -0.04 (s, 3H, Si-C***H₃***); 0.77 (s, 9H, Si-C(C***H₃***)₃); 3.25 (m, 2H, ***H(a)***-C(5') & ***H(b)***-C(5')); 3.73 (s, 6H, 2x -O-C***H₃***); 4.05 - 4.11 (m, 2H, ***H***-C(4') & ***H***-C(3')); 4.47 (t, 1H, ***H***-C(2')); 5.21 (d, 1H, J=6.04 Hz, ***H***O-C(3')); 5.80 (d, 1H, ***H***-C(1')); 6.84-7.37 (m, 14H, ***H***-C(arom.) & ***H***-C(3)); 8.15 (s, 1H, ***H***-C(8)); 11.35 (s, 2H, ***H***N-(TFA) & N***H***(1)) ppm. ^{13}C-NMR: (100 MHz, DMSO-d_6, 25 °C): δ = -5.43 (Si-***C***H_3); -4.93 (Si-***C***H_3); 17.80 (Si-***C***$(CH_3)_3$); 25.56 (Si-C(***C***$H_3)_3$); 54.90 (2 x -O-***C***H_3); 63.43 (***C***(5')); 70.14 (***C***(3')); 75.92 (***C***(2')); 83.99 (***C***(4')); 85.67 (***C***$_{(quat.)}$trityl); 88.53 (***C***(1')); 113.17 – 135.40 (***C***-(arom.)); 138.95 (***C***(8)); 144.74; 156.12; 158.09 ppm.

^{1}H-NMR (400 MHz, DMSO-d_6, 25 °C) of compound **7**

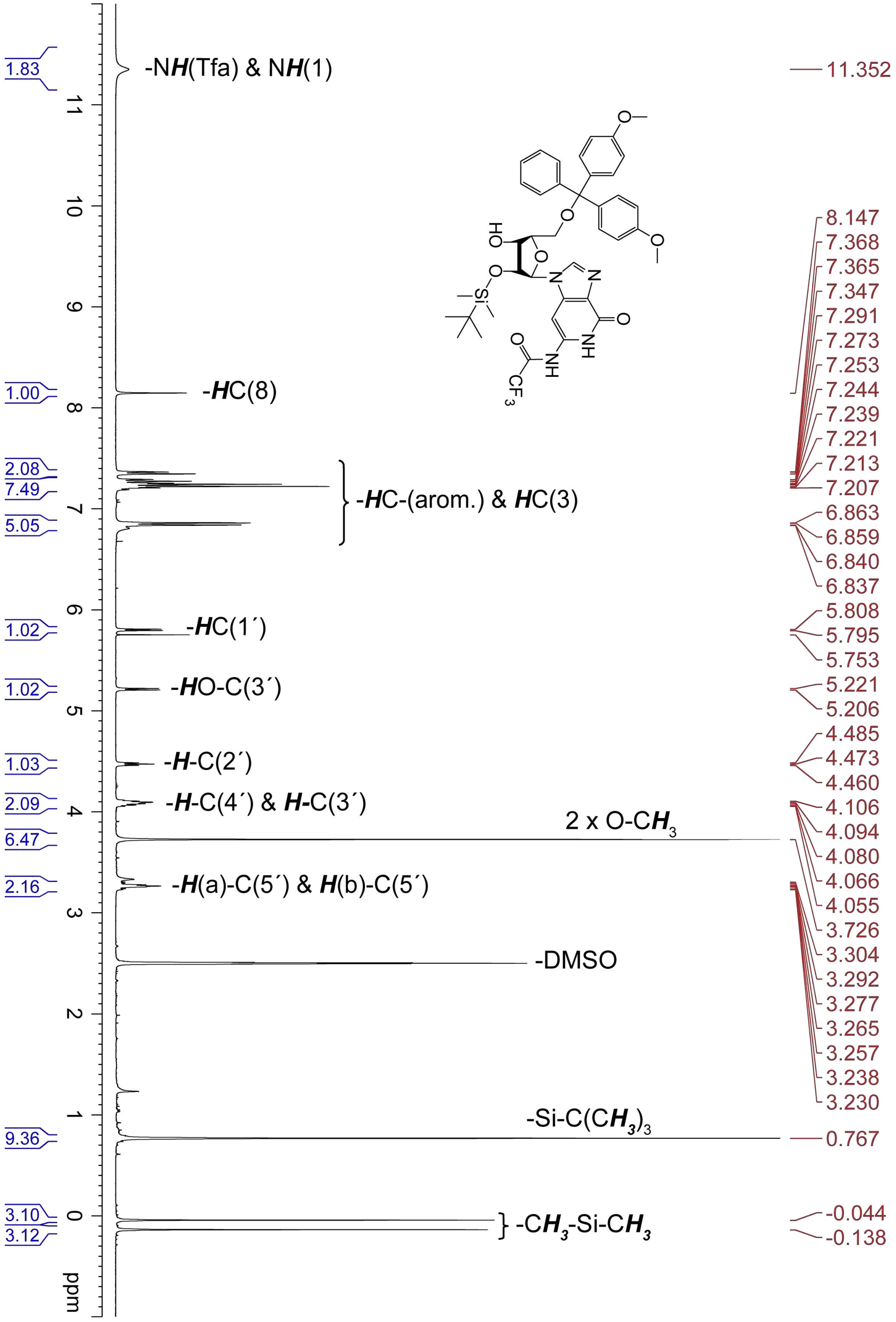

^{13}C-NMR (100 MHz, DMSO-d_6, 25 °C) of compound **7**

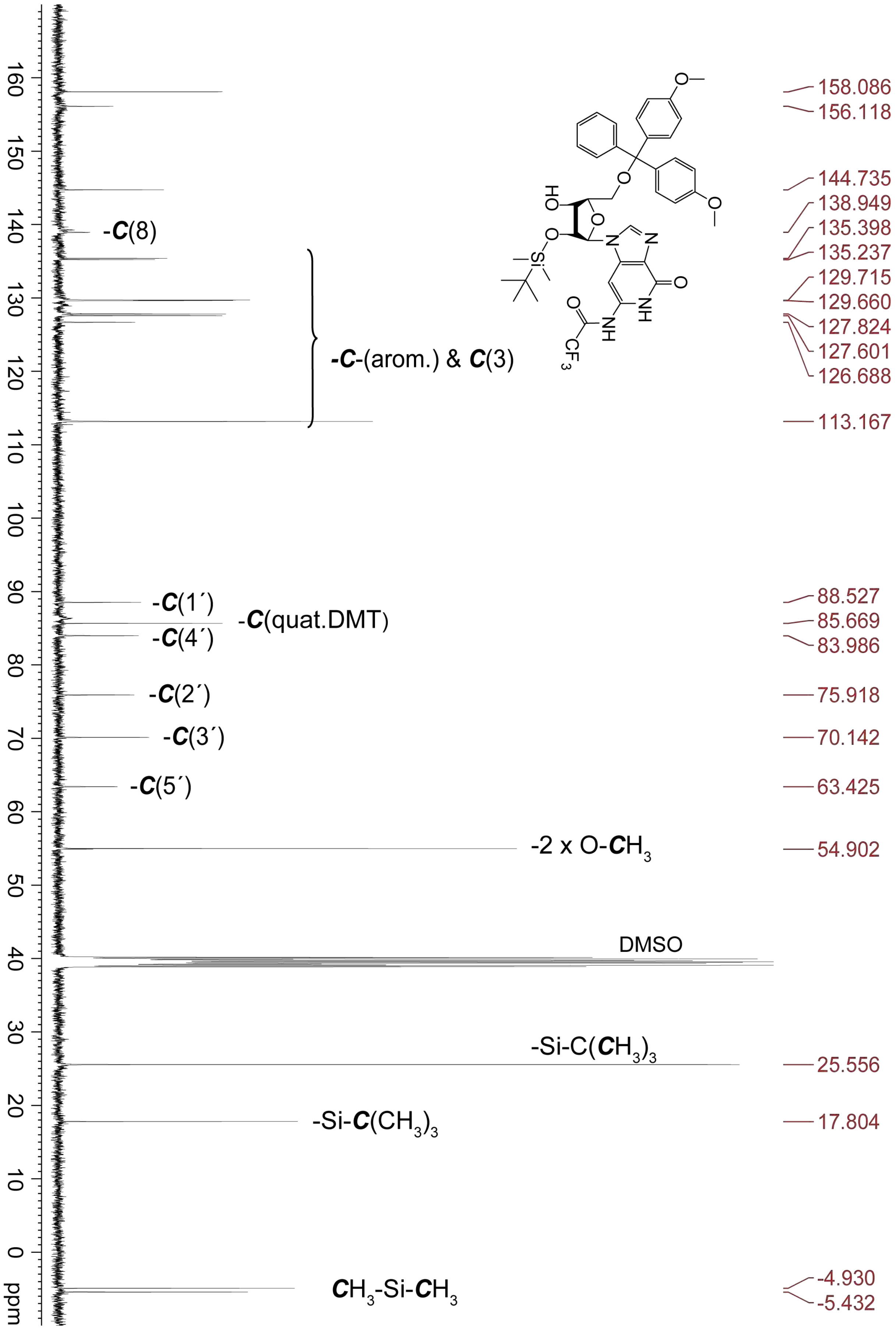

2'-*O*-*tert*-Butyldimethylsilyl-5'-*O*-(4,4'-dimethoxytrityl)-*N*^{2}-trifluoroacetyl-3-deazaguanosine 3'-*O*-2-cyanoethyl-*N*,*N*-di*iso*propylphosphoramidite (8)

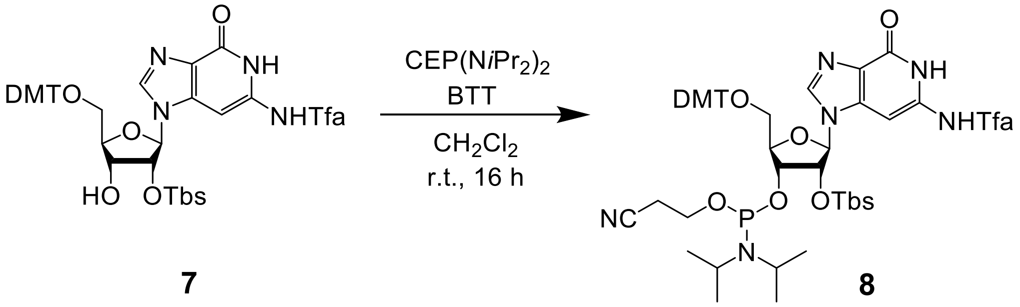

7 **8**

Compound **7** (220 mg, 276.76 µmol) and 5-(benzylthio)-1*H*-tetrazole (BTT, 21.28 mg, 110.71 µmol) were dissolved in dry dichloromethane (7.5 ml), mixed with 3 Å molecular sieve and stirred for two hours under argon atmosphere at room temperature. Then, 2-cyanoethyl *N*,*N*,*N'*,*N'*-tetra*iso*propyl-phosphorodiamidite (CEP(N*i*Pr$_2$)$_2$, 265 µl, 830.29 µmol) was added via syringe and the mixture was stirred at room temperature over night for 16 hours. Afterwards, the solvent was evaporated and the crude product was purified with column chromatography on SiO_2 using chloroform/acetone (7:2) containing 0.5 % triethylamine to remove all reagents, followed by elution of compound **8** with pure acetone. Yield: 218.00 mg of compound **8** as a white foam (79 %). TLC: (CH_2Cl_2/MeOH, 95/5): R_f = 0.25 HR-ESI-MS ([M+H]$^+$ calcd.: 1034.3702 ; found: 1034.3688. ^{1}H-NMR: (400 MHz, $CDCl_3$, 25 °C): δ = -0.22 (s, 3H, Si-C***H*$_3$**); -0.07 (s, 3H, Si-C***H*$_3$**); 0.76 (s, 9H, Si-C(C***H*$_3$**)$_3$); 1.16 - 1.25 (m, 12H, ((C***H*$_3$**)$_2$-CH)$_2$-N); 2.68-2.71 (m, 2H, -C***H*$_2$**CN); 3.25 - 3.40 (m, 2H, ***H(a)***-C(5') & ***H(b)***-C(5')); 3.52 – 3.62 (m, 2H, ((CH$_3$)$_2$-C***H***)$_2$-N); 3.77 (s, 6H, 2x -O-C***H*$_3$**); 3.88 – 4.00 (m, 2H, 2x -C***H*$_2$(a)**-O-P- & -C***H*$_2$(b)**-O-P-); 4.29 - 4.38 (m, 2H, ***H***-C(4') & ***H***-C(3')); 4.56 – 4.63 (m, 1H, ***H***-C(2')); 5.68 – 5.76 (m, 1H, ***H***-C(1')); 6.80 - 7.45 (m, 14H, ***H***-C(arom.) & ***H***-C(3)); 7.97 (s, 1H, ***H***-C(8)) ppm. ^{31}P-NMR: (162 MHz, $CDCl_3$, 25 °C)= 151.71 & 148.54 ppm.

^{1}H-NMR (400 MHz, $CDCl_3$, 25 °C) of compound **8**

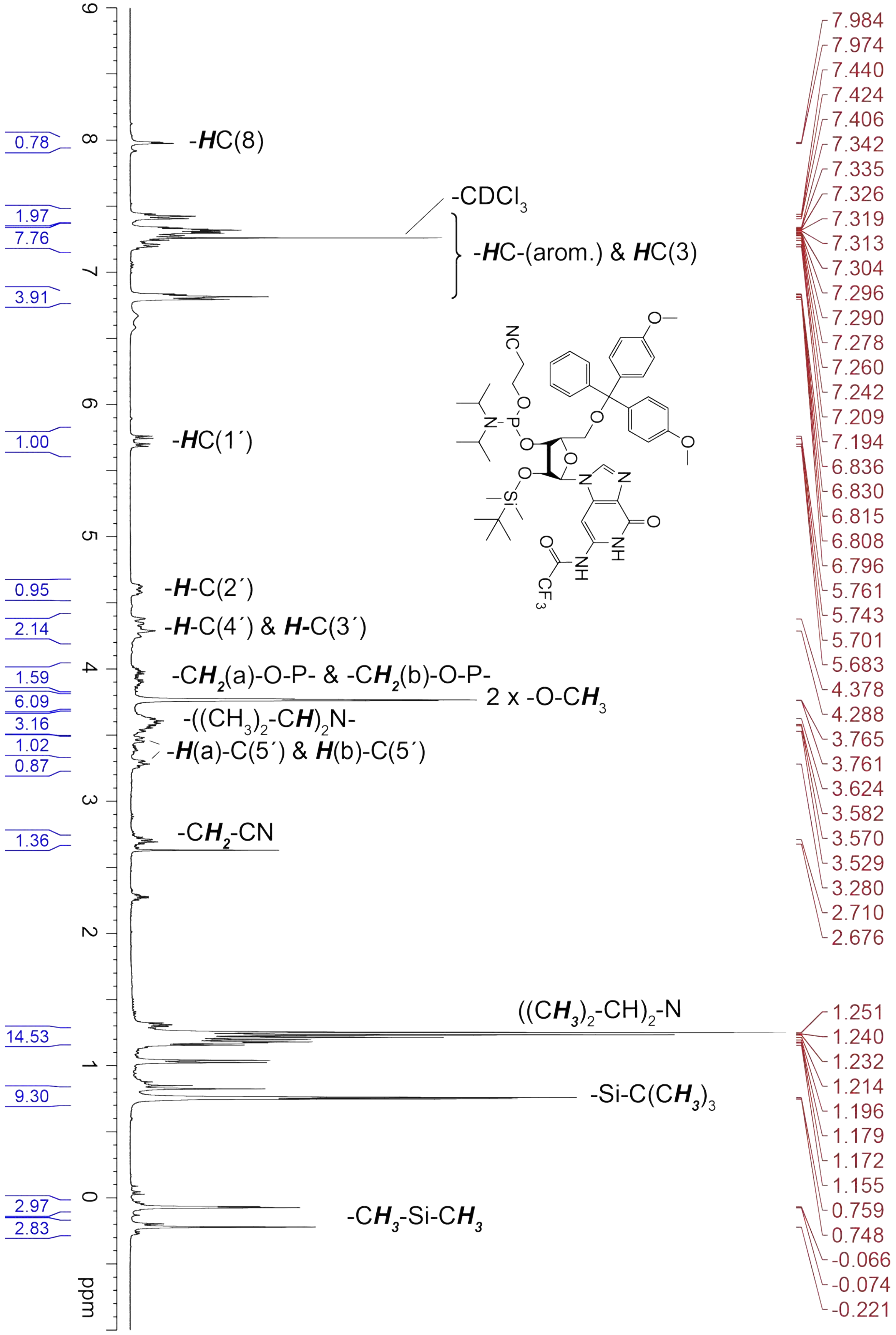

^{31}P-NMR (162 MHz, $CDCl_3$, 25 °C) of compound **9**

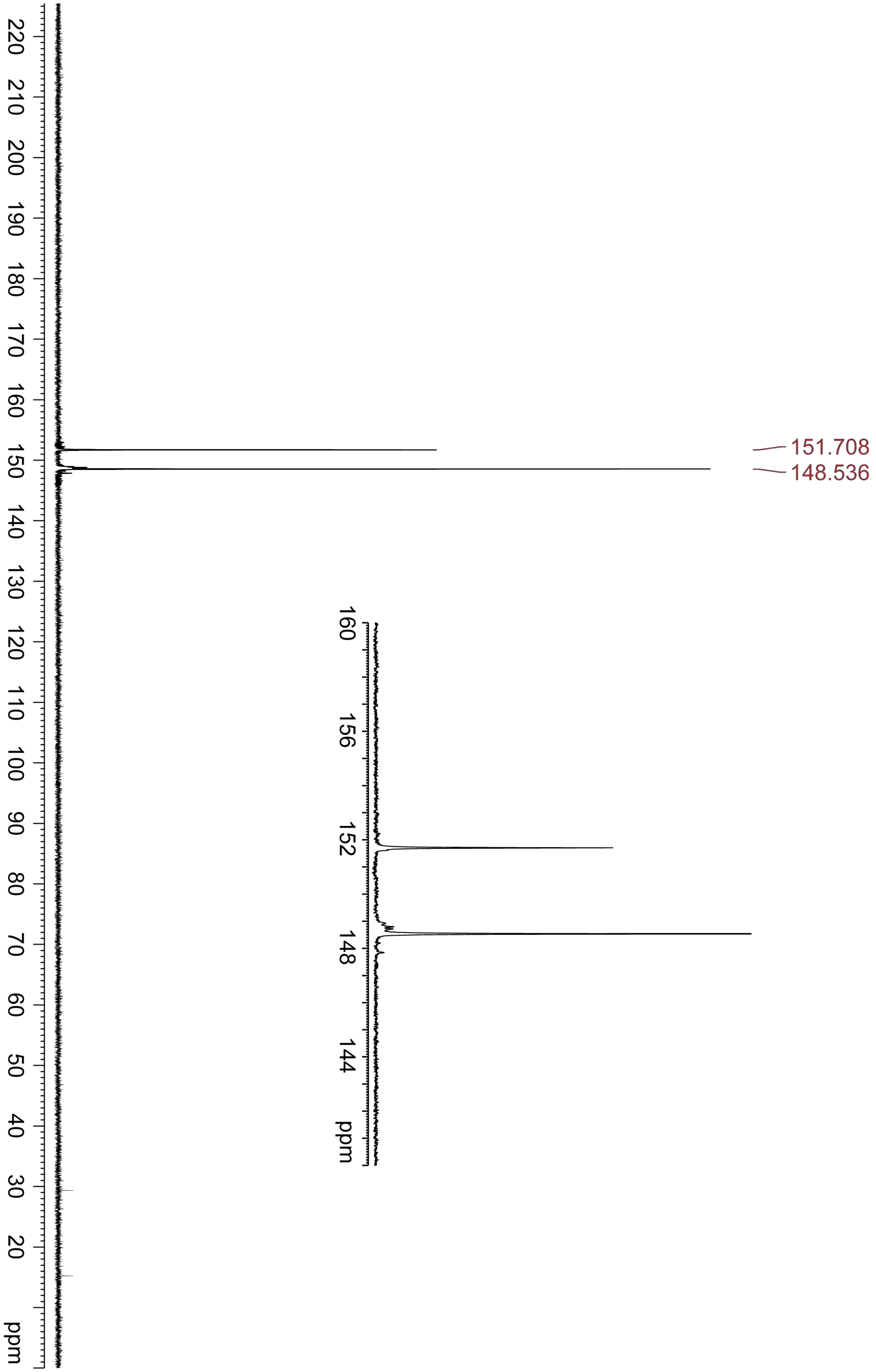

Synthesis of *N*²-Tfa 2'-*O*-Cem 3-deazaguanosine phosphoramidite 9

*O*⁶-Benzyl-*N*²-trifluoroacetyl-3',5'-*O*-(1,1,3,3-tetra-*iso*propyldisiloxane-1,3-diyl)-3-deazaguanosine (4a)

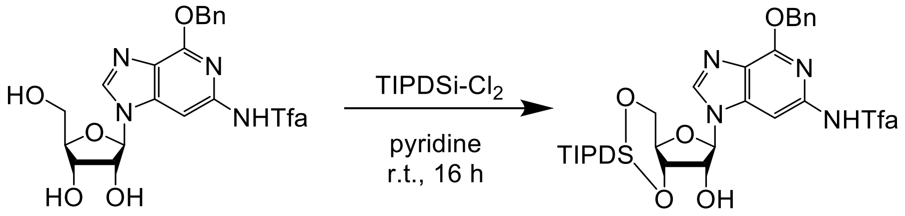

4 **4a**

Compound **4** (2.21 g, 4.72 mmol) was co-evaporated twice with dry pyridine. The resulting residue was dissolved in dry pyridine (40 ml) and 1,3-dichloro-1,1,3,3-tetra-*iso*propyldisiloxane (TIPDSCl$_2$, 1.60 ml, 5.00 mmol) was slowly dropped to the solution. The mixture was stirred under argon atmosphere for 16 hours followed by quenching with methanol (1 ml). The solvent was removed under reduced pressure and the oily residue was co-evaporated twice with toluene, diluted with CH_2Cl_2 (100 ml) and washed consecutively with aqueous saturated $NaHCO_3$ solution (50 ml) and 5 % citric acid (50 ml). The organic layer was dried over Na_2SO_4, filtrated and evaporated to dryness. The crude product was purified by column chromatography on SiO_2 (0 % to 2 % MeOH in CH_2Cl_2).Yield: 2.41 g of compound **4a** as a white foam (72 %). TLC: (CH_2Cl_2/MeOH, 96/4): R_f = 0.49. HR-ESI-MS (m/z): $[M+H]^+$ calcd.: 711.2807 ; found: 711.2785. ^{1}H-NMR: (400 MHz, CDCl$_3$, 25 °C): δ = 1.00 - 1.10 (m, 28H, 4xSi-C***H***(C***H₃***)); 4.08-4.19 (m, 3H, ***H(a)***-C(5') & ***H(b)***-C(5') & ***H***-C(4'); 4.21-4.24 (m, 1H, ***H***-C(3')); 4.54 (m, 1H, ***H***-C(2')); 4.33-4.38 (q, 1H, ***H***-C(2')); 5.53 (s, 2H, -C***H₂***-(benzyl)); 5.86 (d, 1H, J=2.40 Hz, ***H***-C(1')); 7.28-7.38 (m, 3H, ***H***-C(arom.)); 7.49 (d, 2H, ***H***-C(arom.)); 7.95 (s, 1H, ***H***-C(3)); 8.09 (s, 1H, ***H***-C(8)); 8.41 (s, 1H, ***H***N-(TFA)) ppm. ^{13}C-NMR: (100 MHz, CDCl$_3$, 25 °C): δ = 61.62 (***C***(5')); 68.32 (-***C***H$_2$-(benzyl)); 70.43 (***C***(3')); 75.09 (***C***(2')); 82.67 (***C***(4')); 90.33 (***C***(1')); 90.94 (***C***(3)); 127.58-128.57 (m, ***C***-(arom.(benzyl)); 136.72; 140.64; 140.80 (***C***(8)); 154.31 (***C***(6)); 154.64 (***C***(C=O,TFA)) ppm.

^{1}H-NMR (400 MHz, $CDCl_3$, 25 °C) of compound **4a**

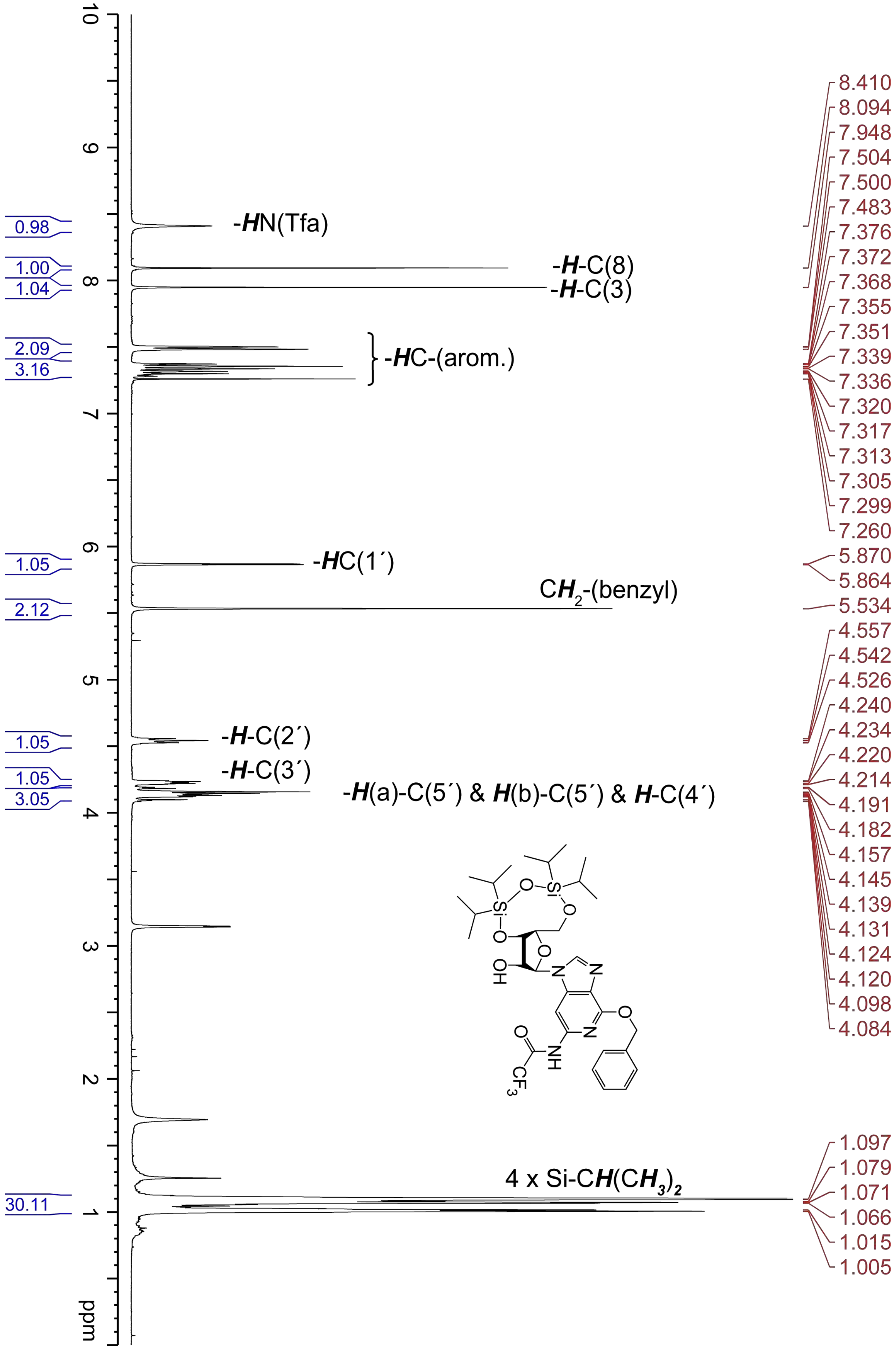

^{13}C-NMR (100 MHz, CDCl$_3$, 25 °C) of compound **4a**

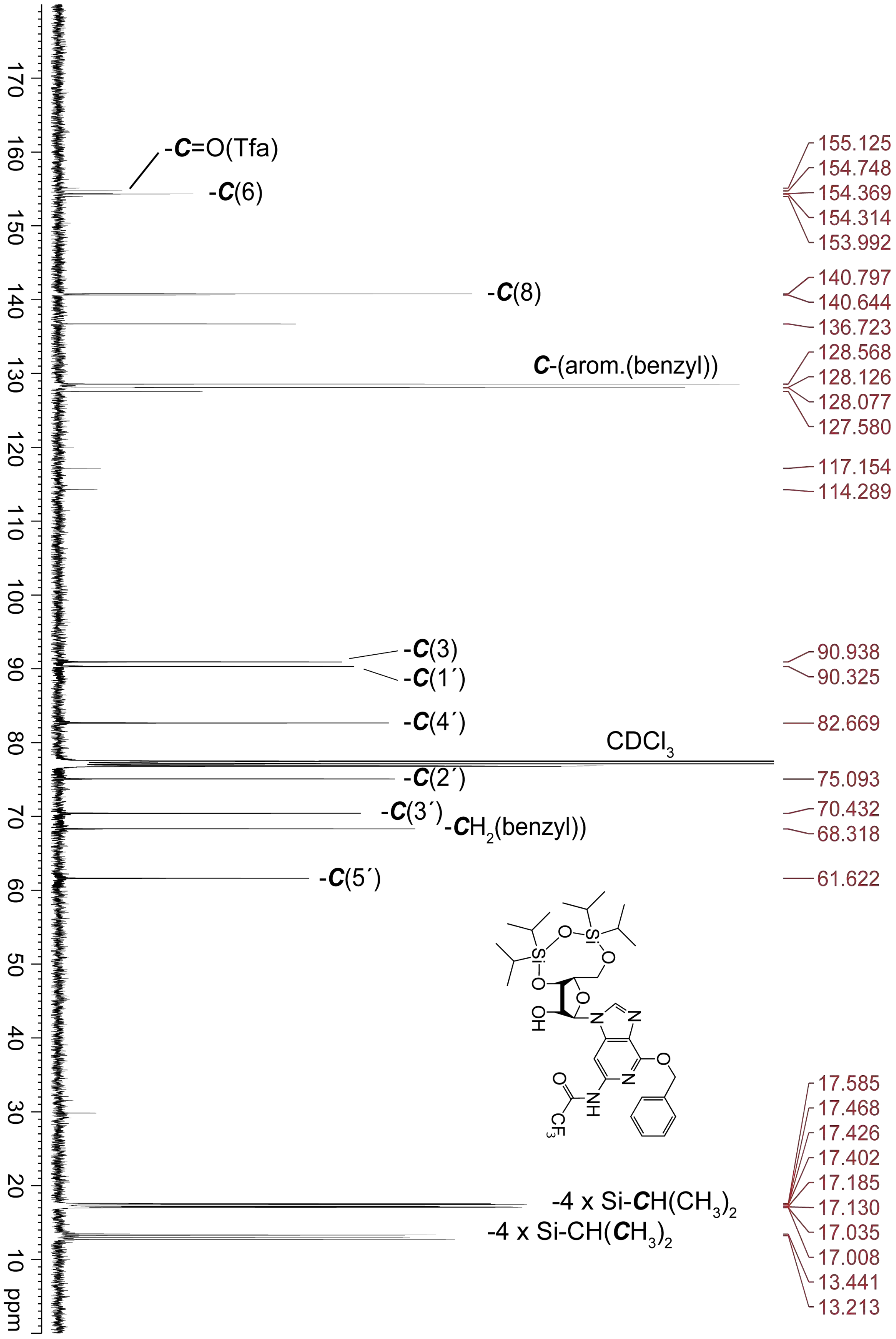

O^6-Benzyl-2'-*O*-(2-cyanoethoxymethyl)-N^2-trifluoroacetyl-3',5'-*O*-(1,1,3,3-tetra-*iso*propyldisiloxane-1,3-diyl)-3-deazaguanosine (4b)

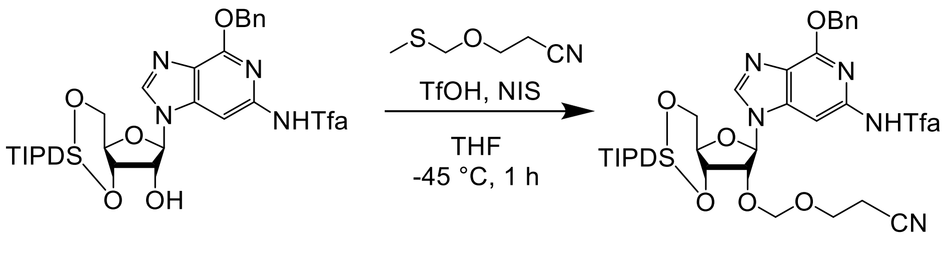

4a **4b**

Compound **4a** (1.30 g, 1.83 mmol) was dissolved in anhydrous tetrahydrofuran and mixed with 2-cyanoethyl methylthiomethylether [2] (374.26 mg, 2.85 mmol). The resulting solution was cooled to -45 °C (freezing mixture made of acetonitrile and liquid nitrogen) and stirred for 30 minutes. Afterwards, trifluoromethanesulfonic acid (TfOH, 252 µl, 2.85 mmol) was dropped to the solution over a period of 10 minutes, whereby the colour of the solution turns into bright orange. Then, *N*-iodosuccinimide (NIS, 641.82 mg, 2.85 mmol) was added and the bright orange turned into a dark brown solution. This dark brown coloured mixture was stirred at -45 °C for further 35 minutes. The reaction was quenched by addition of triethylamine (397 µl, 2.85 mmol) and stirring was continued for another 15 minutes. The quenched mixture was diluted with CH_2Cl_2 (50 ml) and washed consecutively two times with saturated ice cold $Na_2S_2O_3$ solution (50 ml), two times with ice cold saturated $NaHCO_3$ solution (50 ml) and brine (50 ml). The resulting organic layer was dried over Na_2SO_4, evaporated to dryness and purified via column chromatography on SiO_2 (0 % to 30 % ethyl acetate in cyclohexane). Yield: 1.30 g of compound **4b** as a slightly yellow foam (89 %). TLC: (cyclohexane / ethyl acetate, 6/4): R_f = 0.60. HR-ESI-MS (m/z): $[M+H]^+$ calcd.: 794.3178 ; found: 794.3150. ^{1}H-NMR: (400 MHz, $CDCl_3$, 25 °C): δ = 0.94-1.12 (m, 28H, 4xSi-C***H***(C***H₃***)); 2.69 (t, 2H, CH_2C***H₂***CN); 3.79-3.84 (m, 1H, -O-C***H₂***(a)CH_2-CN); 4.02-4.06 (dxd, 1H, ***H(a)***-C(5')); 4.12-4.20 (m, 3H, -O-C***H₂***(b)CH_2-CN & ***H***-C(3') & ***H***-C(4')); 4.32 (d, 1H, J=13.43 Hz, ***H(b)***-C(5)); 4.52 (q, 1H, ***H***-C(2')); 4.95-5.15 (dxd, 2H, -O-C***H₂***-O-); 5.54 (s, 2H, -C***H₂***-(benzyl)); 5.96 (s, 1H, ***H***-C(1')); 7.28-7.38 (m, 3H, ***H***-C(arom.)); 7.49 (d, 2H, ***H***-C(arom.)); 7.88 (s, 1H, ***H***-C(3)); 8.30 (s, 1H, ***H***-C(8)); 8.47 (s, 1H, ***H***N-(TFA)) ppm. ^{13}C-NMR: (100 MHz, $CDCl_3$, 25 °C): δ = 13.52-13.39 (4xSi-CH(***C***H_3)$_2$); 17.00-17.63 (4xSi-***C***H(CH_3)$_2$); 19.00 (-OCH$_2$***C***H$_2$CN); 59.61 (***C***(5')); 63.90 (O***C***H$_2$CH$_2$CN); 68.36 (-***C***H$_2$-(benzyl)); 69.16 (***C***(2')); 79.24 & 81.66 (***C***(3')) & (***C***(4')); 89.51 (***C***(3)); 90.09 (***C***(1')); 95.79 (-O-***C***H$_2$-O-); 114.26; 117.12; 118.03; 127.57-128.44 (m, ***C***-(arom.(benzyl)); 136.71; 140.15 (***C***(8)); 140.47; 140.77; 154.37 (***C***(6)); 154.57 (***C***(C=O,TFA)); 154.95 ppm.

^{1}H-NMR (400 MHz, $CDCl_3$, 25 °C) of compound **4b**

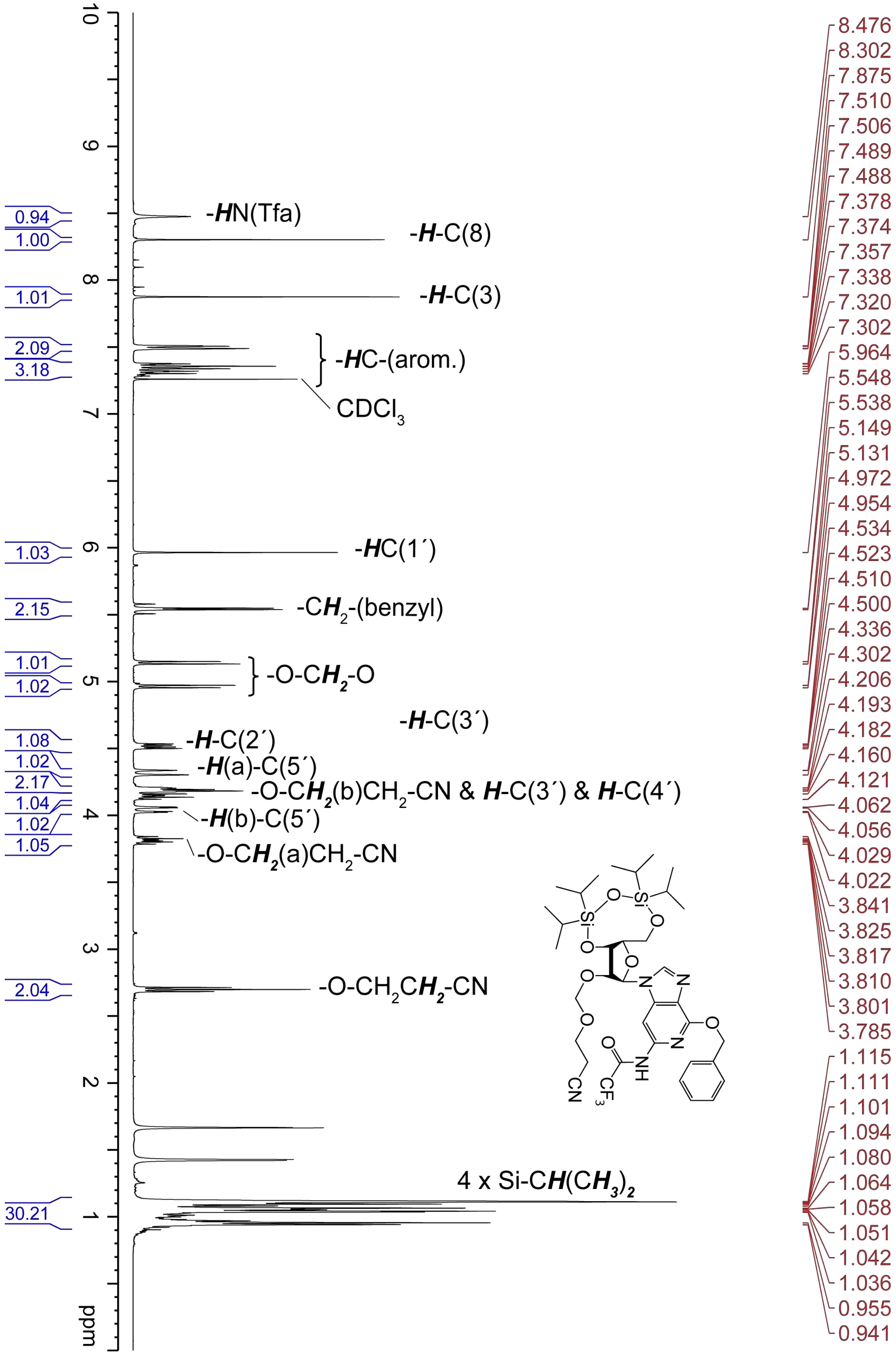

^{13}C-NMR (100 MHz, $CDCl_3$, 25 °C) of compound **4b**

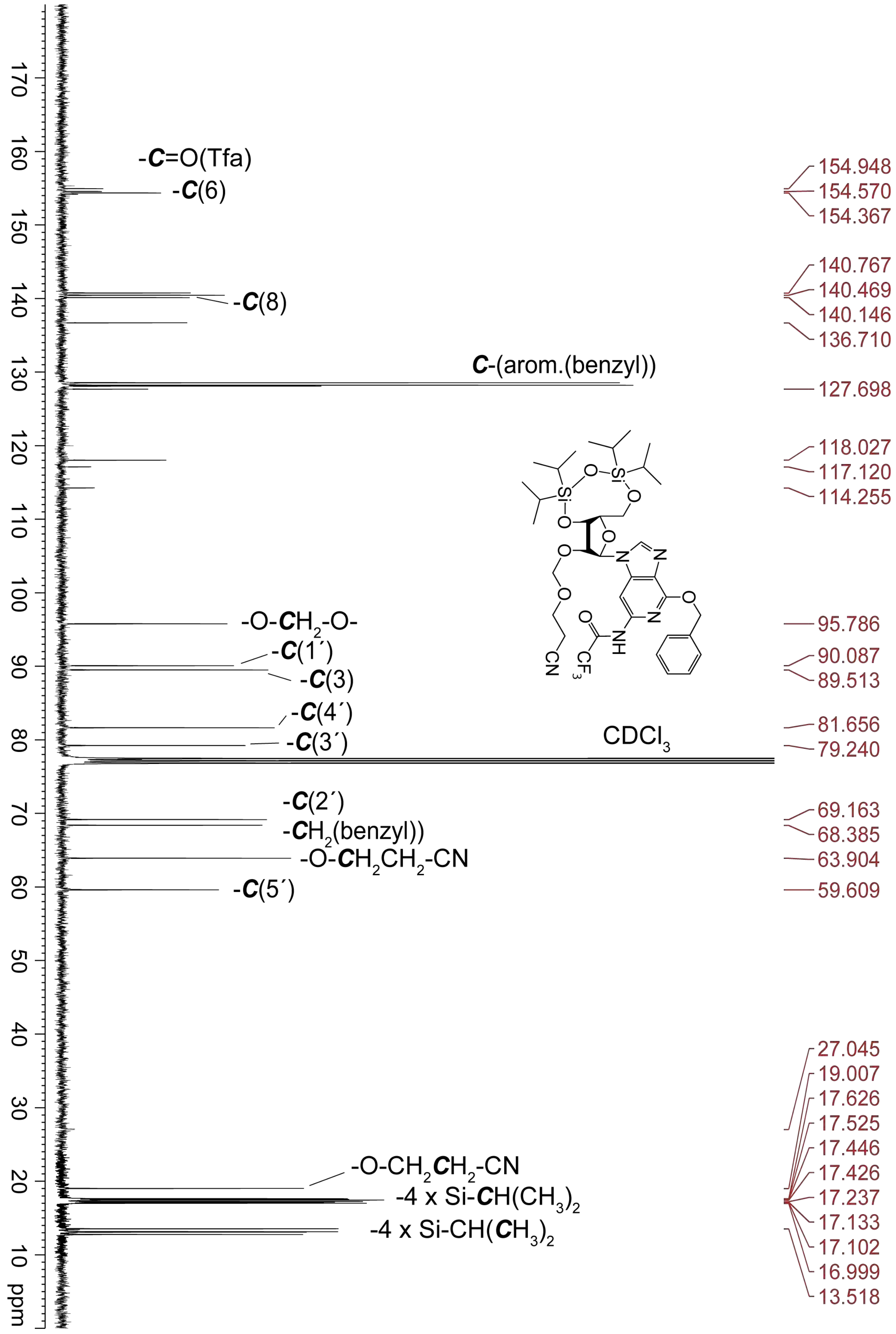

O^6-Benzyl-2'-O-(2-cyanoethoxymethyl)-N^2-trifluoroacetyl-3-deazaguanosine (4c)

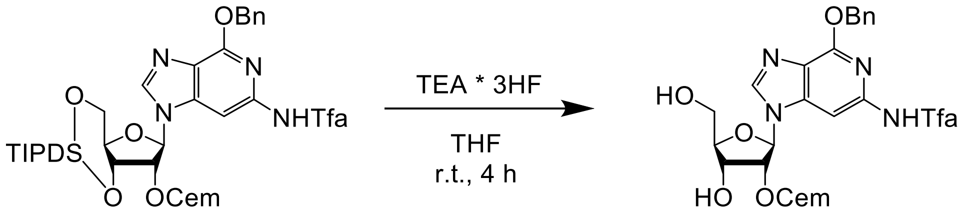

4b **4c**

Compound **4b** (2.46 g, 3.10 mmol) was dissolved in anhydrous tetrahydrofuran (20 ml) and triethylamine trihydrofluoride (TEA * 3HF, 459.52 mg, 468.90 µl, 2.85 mmol) was added and stirred for four hours under argon atmosphere at room temperature. After completion of the reaction, the solvent was evaporated and the crude product was purified by column chromatography on SiO_2 (0 % to 5 % MeOH in CH_2Cl_2). Yield: 1.47 g of compound **4c** as a slightly yellow foam (86 %). TLC: (CH_2Cl_2/MeOH, 95/5): R_f = 0.30. HR-ESI-MS (m/z): $[M+H]^+$ calcd.: 552.1622 ; found: 552.1700. ^{1}H-NMR: (400 MHz, DMSO-d_6, 25 °C): δ = 2.54-2.58 (m, 2H, -OCH_2C***H_2***CN); 3.39-3.41 (m, 1H, -O-C***H_2***(a)CH_2-CN); 3.58-3.63 (m, 2H, -O-C***H_2***(b)CH_2-CN & ***H(a)***-C(5')); 3.63-3.67 (m, 1H, ***H(b)***-C(5')); 4.02 (q, 1H, ***H***-C(4')); 4.29 (q, 1H, ***H***-C(3')); 4.47 (t, 1H, ***H***-C(2')); 4.70-4.76 (q, 1H, -O-C***H_2***-O-); 5.13 (t, 2H, ***H***O-C(5')); 5.43 (d, 2H, J=5.54 Hz, ***H***O-C(3')); 5.59 (s, 2H, -C***H_2***-(benzyl)); 6.03 (s, 1H, ***H***-C(1')); 7.35-7.43 (m, 3H, ***H***-C(arom.)); 7.54 (m, 2H, ***H***-C(arom.)); 7.90 (s, 1H, ***H***-C(3)); 8.53 (s, 1H, ***H***-C(8)); 11.69 (s, 1H, ***H***N-(TFA)) ppm. ^{13}C-NMR: (100 MHz, DMSO-d_6, 25 °C): δ = 17.76 (-OCH_2***C***H_2CN); 60.95 (***C***(5')); 62.42 (O***C***H_2CH_2CN); 67.19 (-***C***H_2-(benzyl)); 68.97 (***C***(3')); 78.96 (***C***(2')); 86.26 (***C***(4')); 86.99 (***C***(1')); 92.73 (***C***(3)); 94.16 (-O-***C***H_2-O-); 118.84; 126.30-128.43 (m, ***C***-(arom.(benzyl)); 136.87; 140.882; 141.02; 142.51 (***C***(8)); 153.23 (***C***(C=O,TFA)) ppm.

^{1}H-NMR (400 MHz, DMSO-d_6, 25 °C) of compound **4c**

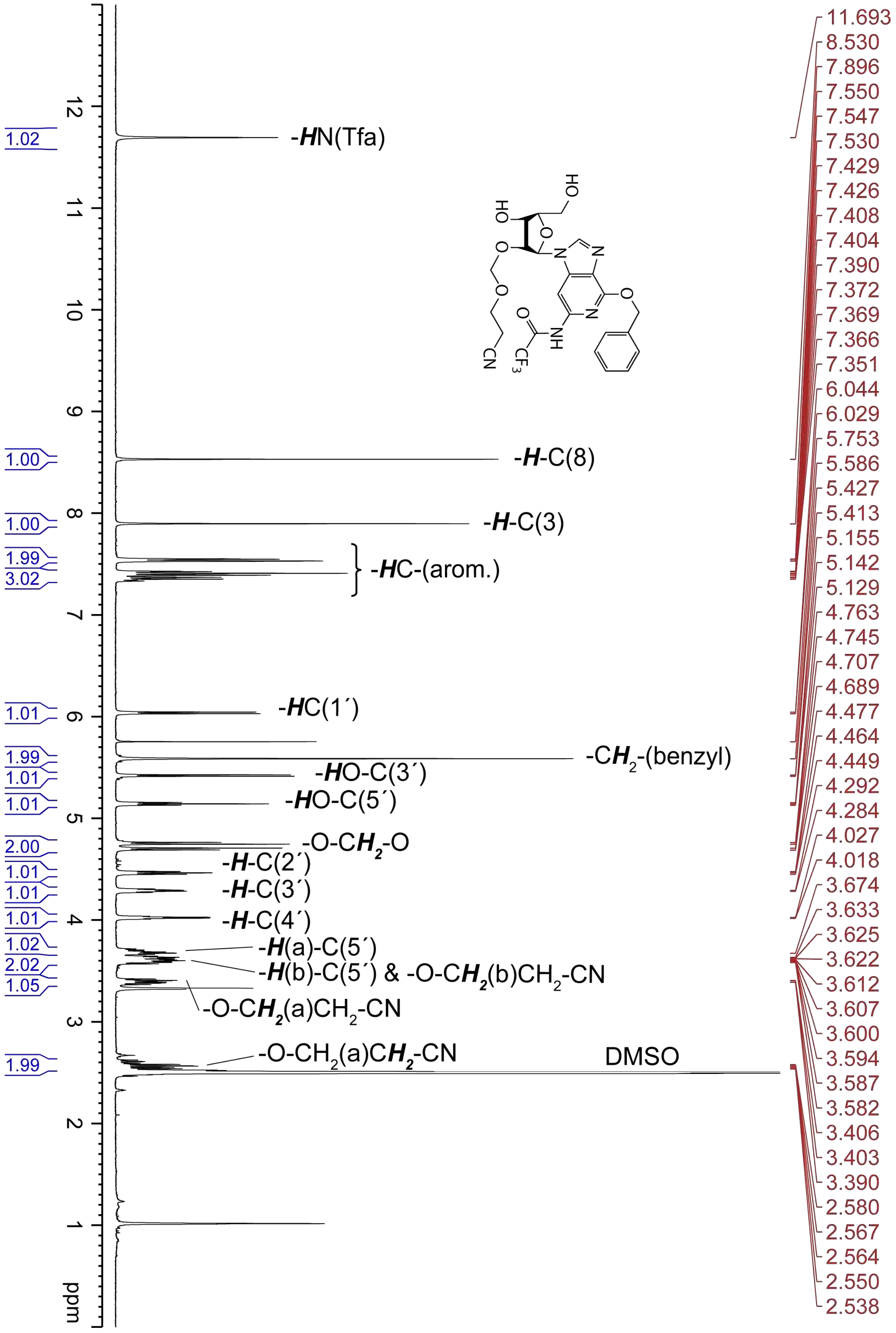

^{13}C-NMR (100 MHz, DMSO-d_6, 25 °C) of compound **4c**

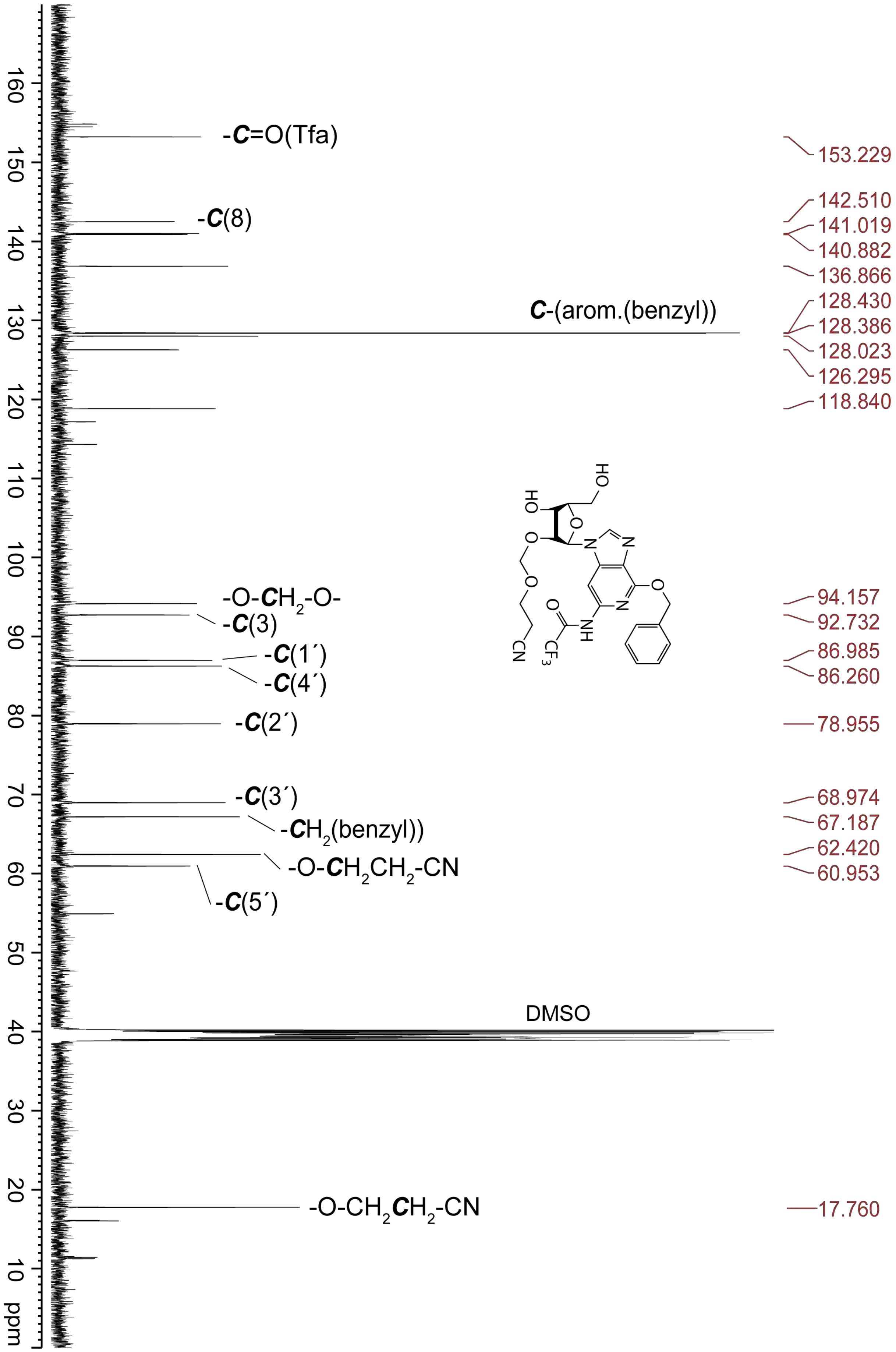

O^6-Benzyl-2'-O-(2-cyanoethoxymethyl)-5'-O-(4,4'-dimethoxytrityl)-N^2-trifluoroacetyl-3-deazaguanosine (4d)

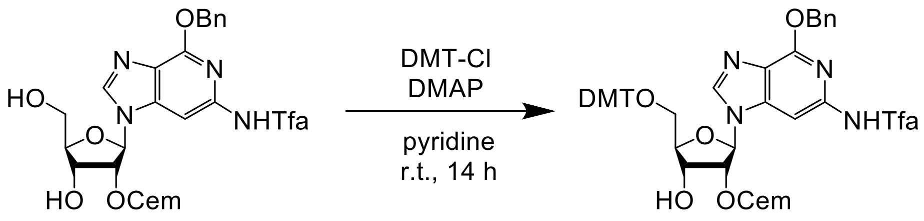

4c **4d**

Compound **4c** (954.00 mg, 1.73 mmol) and 4-(dimethylamino)pyridine (DMAP, 46.00 mg, 0.38 mmol) were transferred into a 100 ml round bottom flask and co-evaporated three times with dry pyridine and subsequently dissolved in dry pyridine (45 ml). Afterwards, 4,4'-dimethoxytrityl chloride (DMT-Cl, 879.21 mg, 2.59 mmol) was added in three portions at room temperature over a period of three hours. Stirring was continued for 16 hours at the same temperature and the reaction was finally quenched by the addition of MeOH (1 ml). The solvents were evaporated and the resulting residue was diluted with CH_2Cl_2 (100 ml) and transferred into a separating funnel. The dissolved crude product was washed first with 5 % citric acid (50 ml), followed by saturated $NaHCO_3$ solution and finally with brine (50 ml). The organic layer was dried over Na_2SO_4, evaporated to dryness and purified by column chromatography on SiO_2 (0 % to 5 % MeOH in CH_2Cl_2). Yield: 960.00 mg of compound **4d** as a yellow foam (65 %). TLC: (CH_2Cl_2/MeOH, 96/4): R_f = 0.42. HR-ESI-MS (m/z): $[M+H]^+$ calcd.: 854.2963 ; found: 854.2999. ^{1}H-NMR: (400 MHz, DMSO-d_6, 25 °C): δ = 2.61-2.67 (m, 2H, -OCH_2C***H_2***CN); 3.23 (m, 2H, ***H(a)***-C(5') & ***H(b)***-C(5')); 3.56-3.59 (m, 1H, -O-C***H_2***(a)CH_2-CN); 3.69-3.73 (m, 7H, 2x -O-C***H_3*** & O-C***H_2***(a)CH_2-CN); 4.12 (q, 1H, ***H***-C(4')); 4.33 (q, 1H, ***H***-C(3')); 4.64 (t, 1H, ***H***-C(2')); 4.82 (q, 1H, -O-C***H_2***-O-); 5.43 (d, 1H, J=6.47 Hz, ***H***O-C(3')); 5.58 (d, 2H, J=4.63 Hz, C***H_2***-(benzyl); 6.12 (d, 1H, ***H***-C(1')); 6.76-7.54 (m, 18H, ***H***-C(arom.)); 7.94 (s, 1H, ***H***-C(3)); 8.41 (s, 1H, ***H***-C(8)); 11.72 (s, 1H, ***H***N-(TFA)) ppm. ^{13}C-NMR: (100 MHz, DMSO-d_6, 25 °C): δ = 17.94 (-OCH_2***C***H_2CN); 54.92 (2 x -O-***C***H_3); 62.52 (O***C***H_2CH_2CN); 63.06 (***C***(5')); 67.23 (-***C***H_2-(benzyl)); 69.24 (***C***(3')); 78.38 (***C***(2')); 83.55 (***C***(4')); 85.50 (***C***(1')); 87.29 (***C***$_{(quart.)}$trityl); 92.66 (***C***(3)); 94.32 (-O-***C***H_2-O-); 113.06-129.62 (***C***-(arom.)); 135.32; 135.45; 136.12; 136.83; 141.10; 141.34; 141.71 (***C***(8)); 144.72; 149.61; 153.23 (***C***(C=O,TFA)); 158.00 ppm.

^{1}H-NMR (400 MHz, DMSO-d_6, 25 °C) of compound **4d**

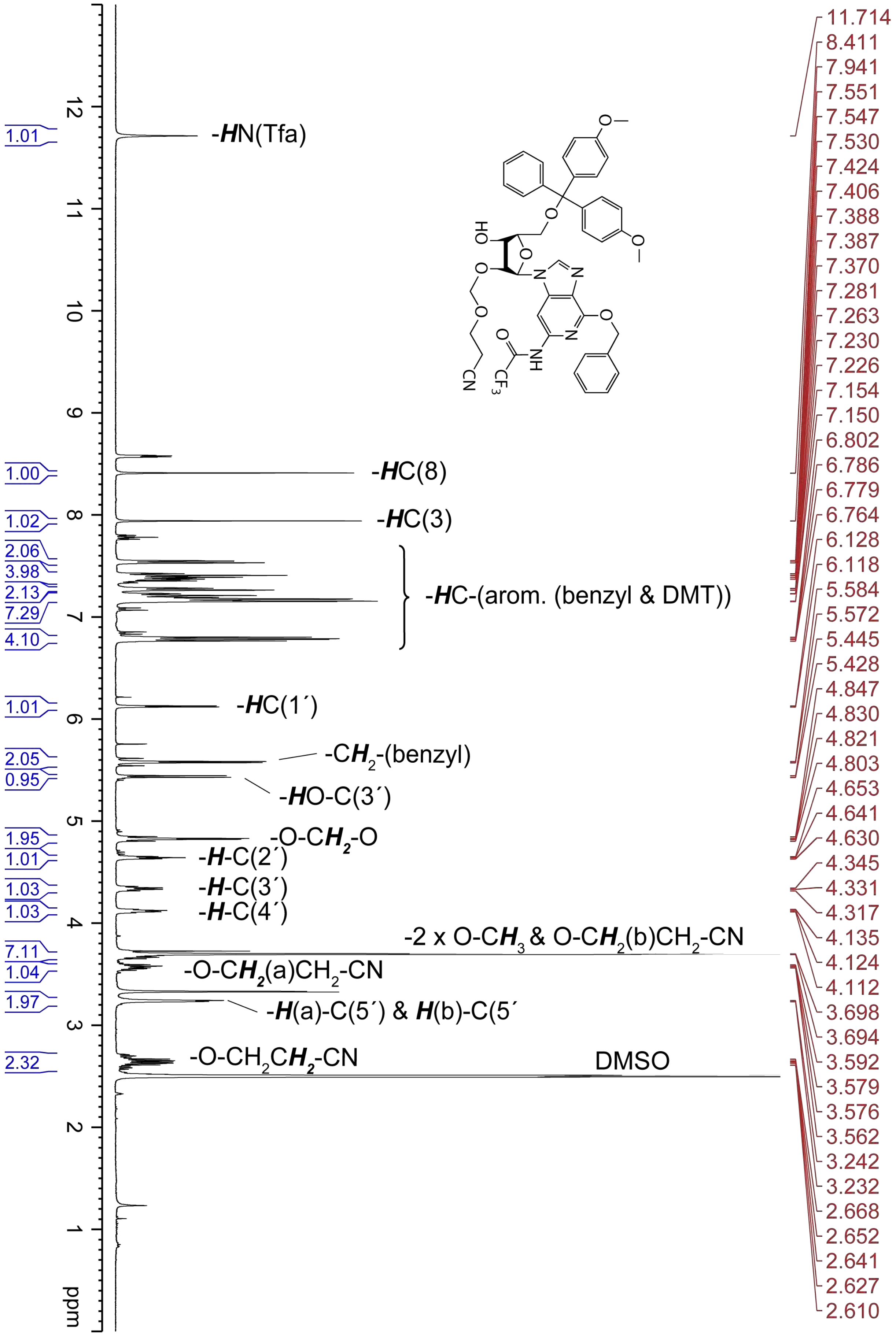

^{13}C-NMR (100 MHz, DMSO-d_6, 25 °C) of compound **4d**

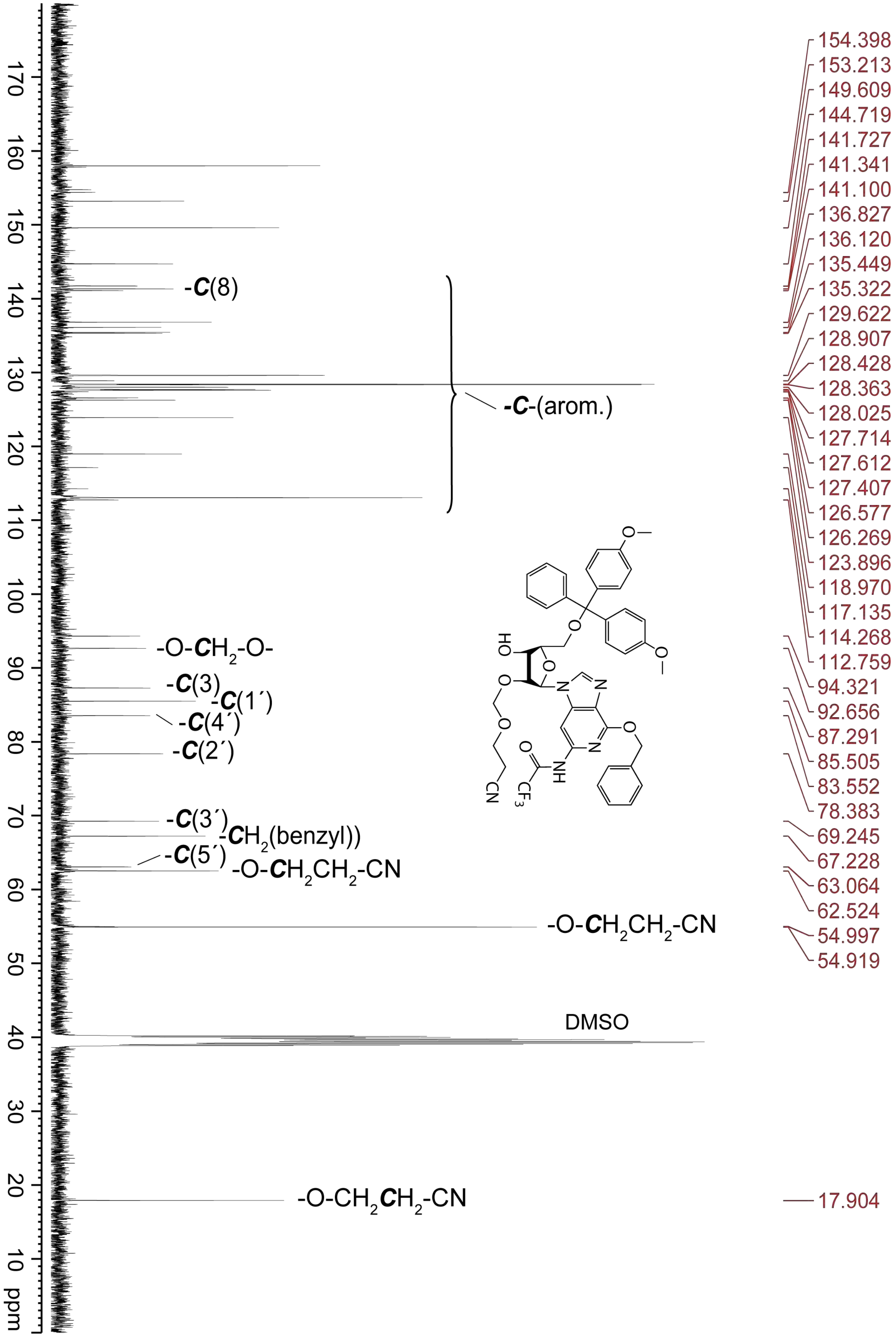

2'-*O*-(2-Cyanoethoxymethyl)-5'-*O*-(4,4'-dimethoxytrityl)-*N*^{2}- trifluoroacetyl-3-deazaguanosine (4e)

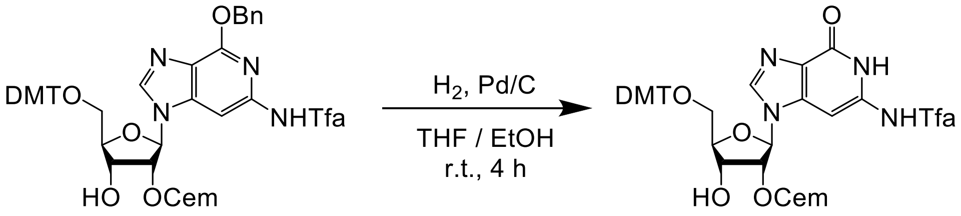

4d **4e**

Compound **4d** (1.04 g, 1.22 mmol) was dissolved in a mixture of anhydrous tetrahydrofuran (16 ml) and dry ethanol (4 ml). Subsequently, palladium on charcoal (476.03 mg, 0.40 mmol, 10 % Pd) was added and hydrogen gas (balloon via syringe) was bubbled through a rubber septum to the reaction mixture for 10 minutes followed by stirring under hydrogen atmosphere for four hours. Reaction control by thin layer chromatography showed complete consumption of the starting material. The resulting dark suspension was filtered over celite to remove the catalyst and the filtrate was evaporated to dryness. The crude product was purified by column chromatography on SiO_2 (2 % to 8 % MeOH in CH_2Cl_2). Yield: 600.00 mg of compound **4e** as a white foam (65 %). TLC: (CH_2Cl_2/MeOH, 97/3): R_f = 0.35. HR-ESI-MS (m/z): $[M+Na]^+$ calcd.: 786.2357 ; found: 786.2347. ^{1}H-NMR: (400 MHz, DMSO-d_6, 25 °C): δ = 2.69-2.74 (m, 2H, -OCH_2C***H_2***CN); 3.23 (m, 2H, ***H(a)***-C(5´) & ***H(b)***-C(5´)); 3.54-3.59 (m, 1H, -O-C***H_2***(a)CH_2-CN); 3.65-3.72 (m, 7H, 2x -O-C***H_3*** & O-C***H_2***(a)CH_2-CN); 4.09 (q, 1H, ***H***-C(4´)); 4.32 (q, 1H, ***H***-C(3´)); 4.56 (t, 1H, ***H***-C(2´)); 4.82 (q, 1H, -O-C***H_2***-O-); 5.42 (d, 1H, J=6.25 Hz, ***H***O-C(3´)); 5.99 (d, 1H, J=5.50 Hz, ***H***-C(1´)); 6.81-6.84 (m, 5H, ***H***-C(arom.)); 7.19-7.33 (m, 9H, ***H***-C(arom.) & H-C(3)); 8.17 (s, 1H, ***H***-C(8)); 11.33 (s, 2H, ***H***N-(TFA) & ***H***N(1)) ppm. ^{13}C-NMR: (100 MHz, DMSO-d_6, 25 °C): δ = 17.92 (-OCH_2***C***H_2CN); 54.97 (2 x -O-***C***H_3); 62.52 (O***C***H_2CH_2CN); 63.24 (***C***(5´)); 69.14(***C***(3´)); 78.59 (***C***(2´)); 83.63 (***C***(4´)); 85.59 (***C***$_{(quart.)}$trityl); 86.91 (***C***(1´)); 94.25 (-O-***C***H_2-O-); 113.14-135.44 (***C***-(arom.)); 138.82 (***C***(8)); 156.09; 158.04 ppm.

^{1}H-NMR (400 MHz, DMSO-d_6, 25 °C) of compound **4e**

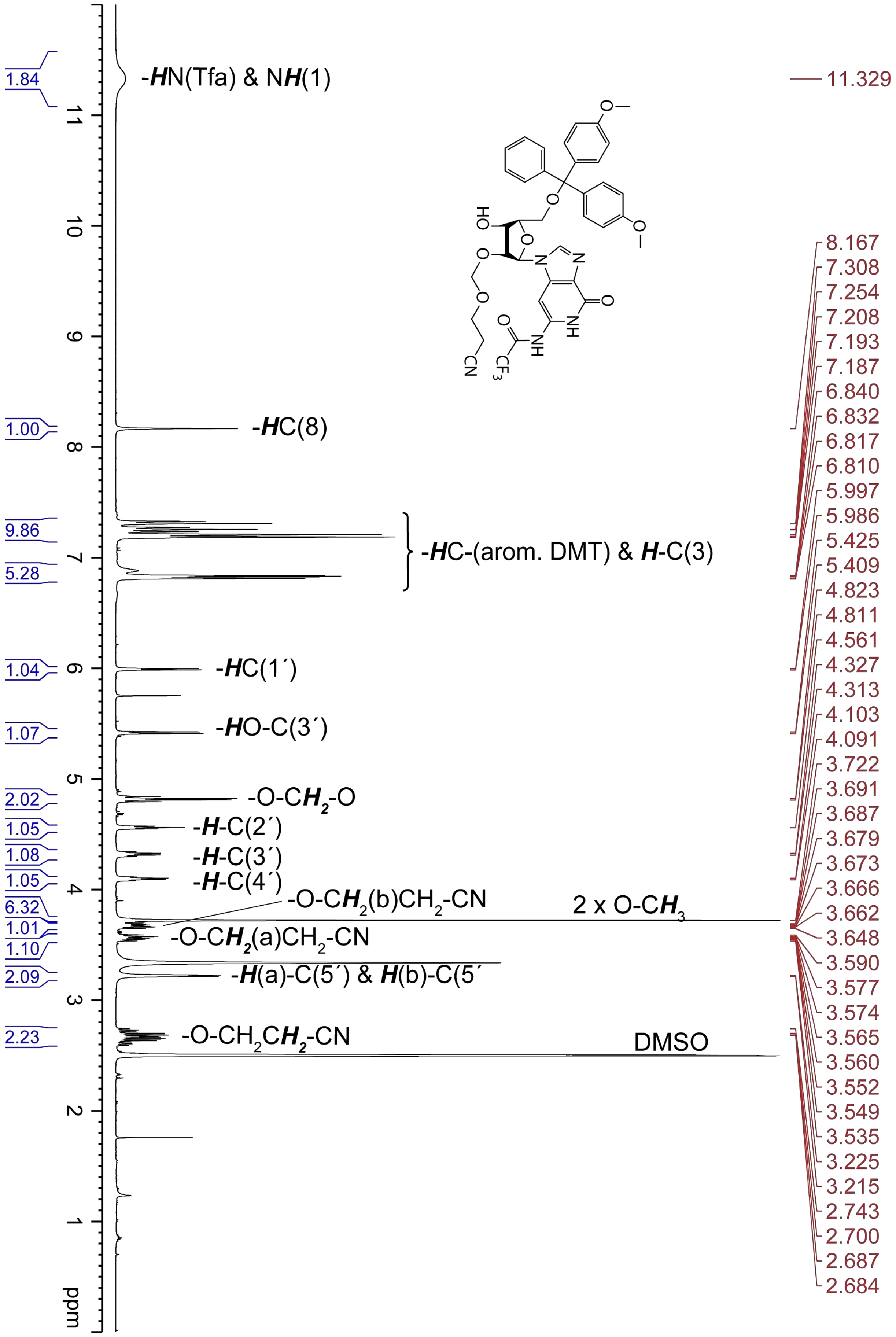

^{13}C-NMR (100 MHz, DMSO-d_6, 25 °C) of compound **4e**

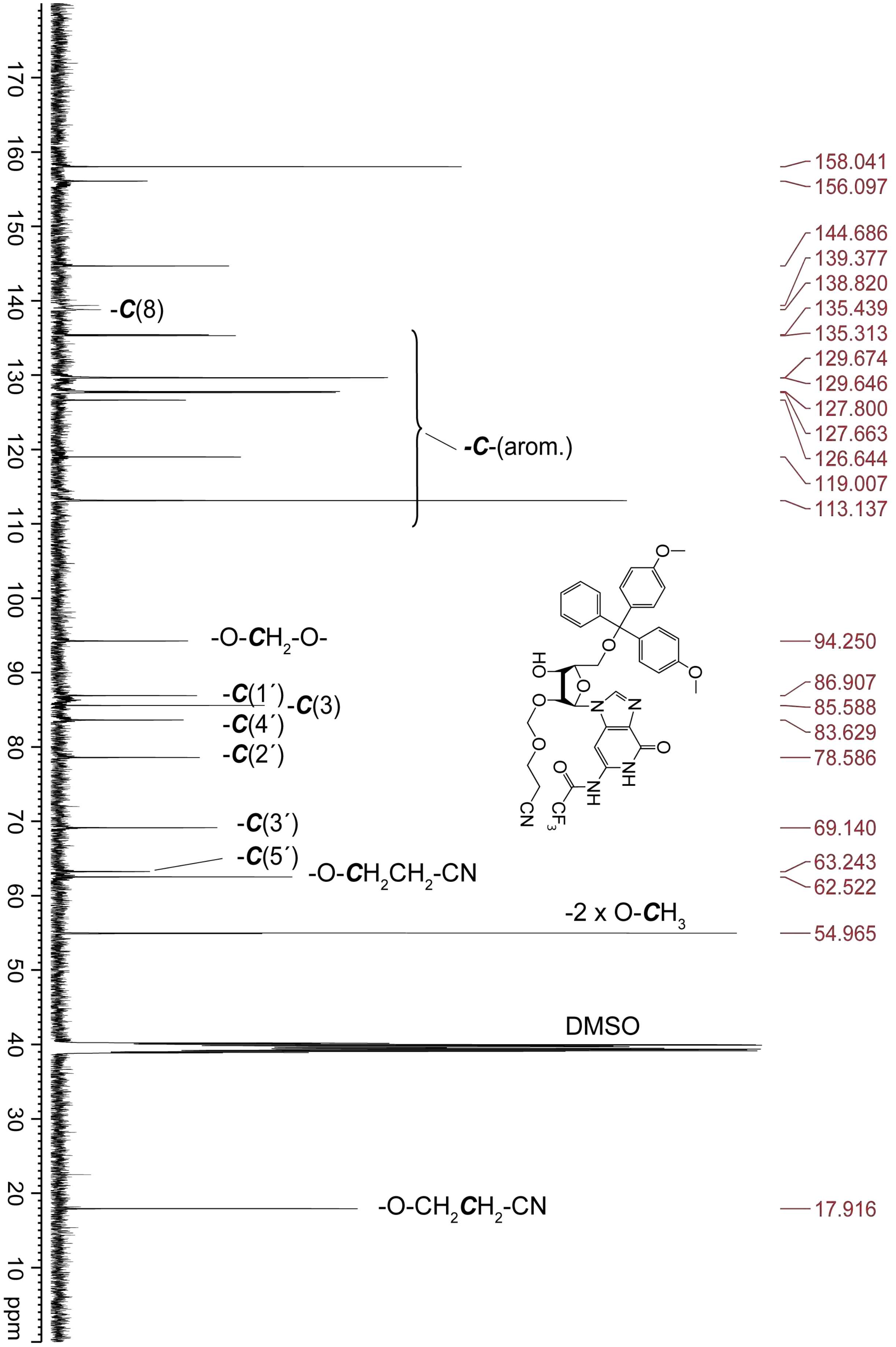

2’-*O*-(2-Cyanoethoxymethyl)-5’-*O*-(4,4’-dimethoxytrityl)-*N*²-trifluoroacetyl-3-deazaguanosine-3’-*O*-2-cyanoethyl-*N*,*N*-di*iso*propylphosphoramidite (9)

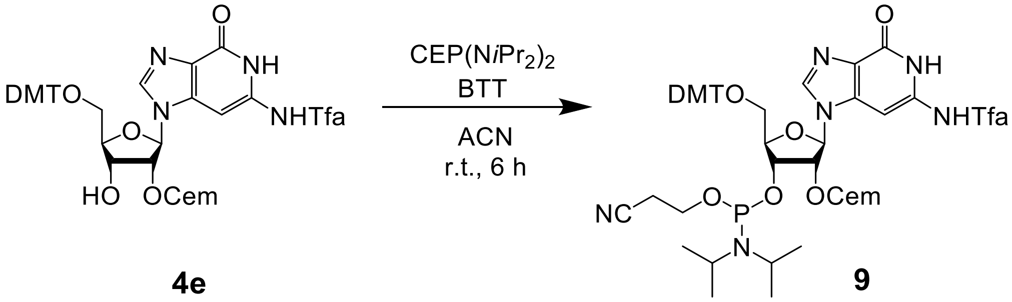

Compound **4e** (112.60 mg, 147.43 µmol) and 5-(benzylthio)-1*H*-tetrazole (BTT, 11.34 mg, 58.97 µmol) were dissolved in dry acetonitrile (3.0 ml) and mixed with 3 Å molecular sieve and stirred for two hours under argon atmosphere at room temperature. Then, 2-cyanoethyl *N*,*N*,*N*',*N*'-tetra*iso*propyl-phosphorodiamidite (CEP(N*i*Pr$_2$)$_2$, 141 µl, 442.30 µmol) was added via syringe and the mixture was stirred at room temperature for six hours. Afterwards, the solvent was evaporated and the crude product was purified with column chromatography on SiO_2 using chloroform/acetone (7:3) containing 0.5 % triethylamine to remove all reagents, followed by eluting of compound **9** with pure acetone. Yield: 125.00 mg of compound **9** as a white foam (88 %). TLC: (CH_2Cl_2/MeOH, 95/5): R_f = 0.25 HR-ESI-MS (m/z): $[M+H]^+$ calcd.: 964.3572, found: 964.3579; $[M+Na]^+$ calcd.: 986.3436, found: 986.3579; $[M+K]^+$ calcd.: 1002.3175, found: 1002.3124 ^{1}H-NMR: (400 MHz, $CDCl_3$, 25 °C): δ = 1.04-1.29 (m, 12H, ((C***H₃***)$_2$-CH)$_2$-N); 2.37-2.67 (m, 4H, -OCH$_2$C***H₂***CN (CEM) & -C***H₂***CN); 3.33-3.57 (m, 6H, ***H(a)***-C(5´) & ***H(b)***-C(5´) & ((CH$_3$)$_2$-C***H***)$_2$-N- & -O-C***H₂(a)***CH$_2$-CN & -O-C***H₂(b)***CH$_2$-CN); 3.77-3.90 (m, 8H, 2x -O-C***H₃*** & -C***H₂(a)***-O-P- & -C***H₂(b)***-O-P-); 4.30-4.38 (m, 1H, ***H***-C(4´)); 4.44-4.92 (m, 4H, ***H***-C(3´) & ***H***-C(2´), -O-C***H₂***-O-); 5.84-5.87 (m, 1H, ***H***-C(1´)); 6.56 (m, 1H, ***H***-C(3)) 6.62-6.83 (m, 5H, ***H***-C(arom.)); 7.20-7.42 (m, 10H, ***H***-C(arom.)); 7.99 (2s, 1H, ***H***-C(8)) ppm. ^{31}P-NMR: (162 MHz, $CDCl_3$, 25 °C)= 150.36 & 150.91 ppm.

^{1}H-NMR (400 MHz, $CDCl_3$, 25 °C) of compound **9** (both diastereomers)

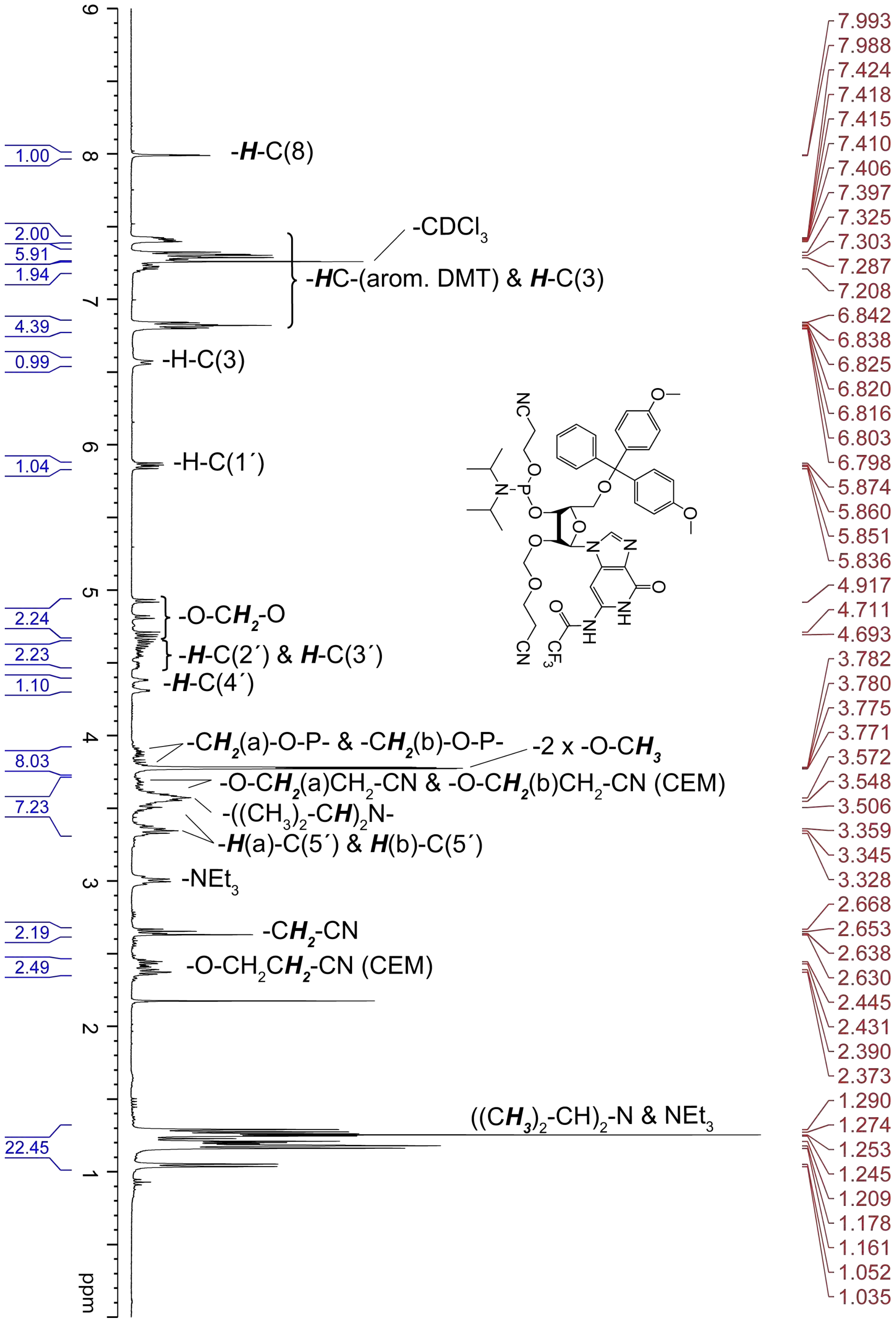

^{31}P-NMR (162 MHz, $CDCl_3$, 25 °C) of compound **9** (both diastereomers)

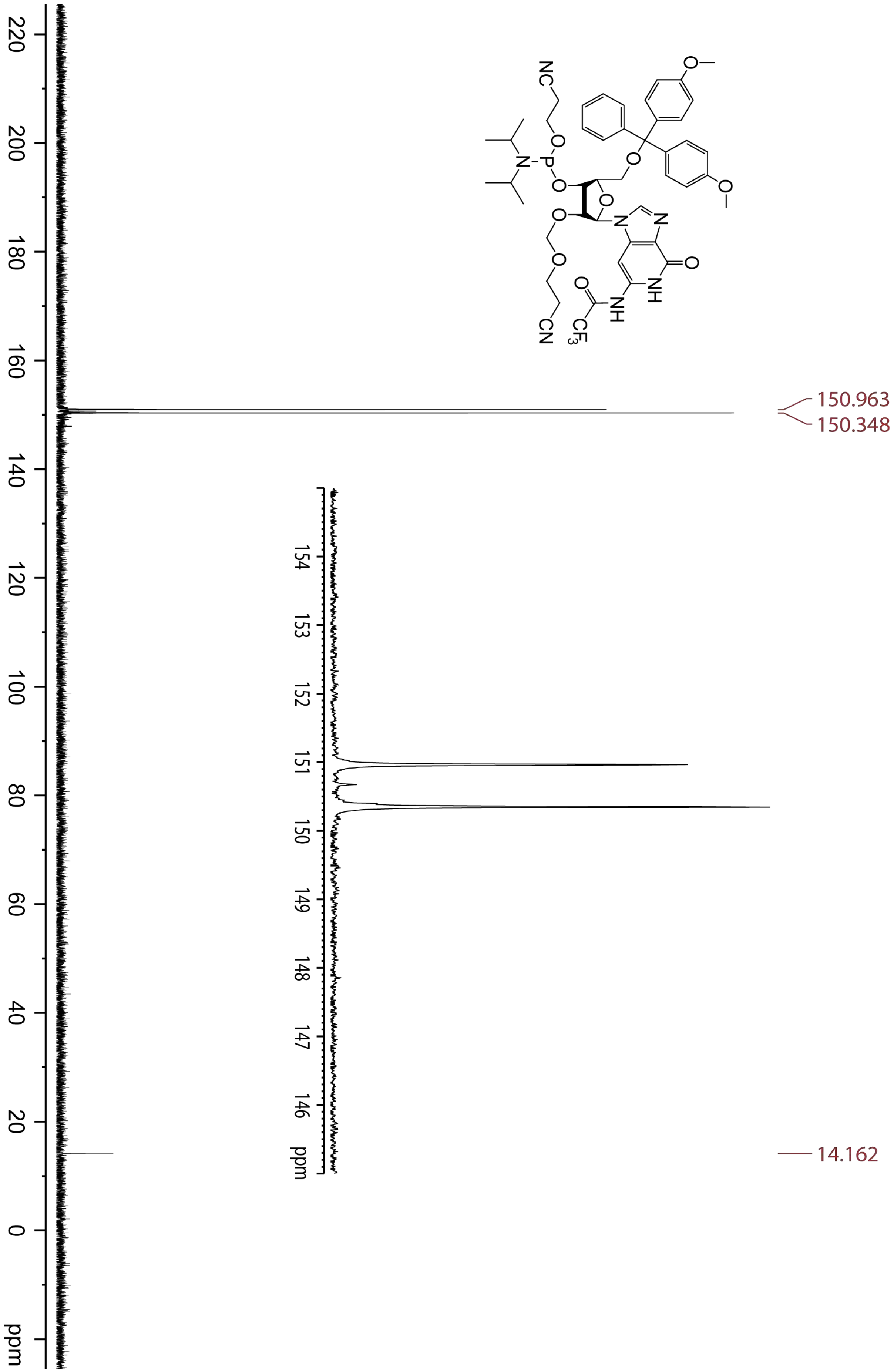

Synthesis of 3-deazaadenosine phosphoramidite 17

1*H*-Imidazo[4,5-c]pyridine (11)

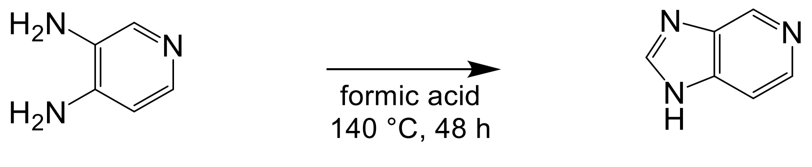

11

3,4-Diaminopyridine (5.00 g, 45.8 mmol) was dissolved in formic acid (17.3 mL) and stirred at 140 °C for 16 hours. The solid residues were dissolved in methanol and evaporated to dryness. This operation was repeated twice. The crude product was purified by column chromatography on SiO_2 (10 % to 20 % methanol in dichloromethane). Yield: 5.30 g of compound **11** as slight yellow solid (97 %). TLC: (CH_2Cl_2 / MeOH, 4/1): R_F = 0.50. ESI-MS (m/z): $[M+H]^+$ calcd.: 120.0556; found: 120.0559. ^{1}H-NMR: (400 MHz, DMSO-d_6, 25 °C): δ 7.59 (1H, d, J=5.45 Hz, ***H***-C(7)), 8.30 (1H, d, J=5.54 Hz, ***H***-C(6)), 8.40 (1H, s, ***H***-C(4)), 8.95 (1H, s, ***H***-C(2)), 12.86 (1H, b, ***H***-N(1)). ^{13}C-NMR: (100 MHz, DMSO-d_6 + 3% HCl (5 %), 25 °C): δ 110.10 (1C, s, ***C***(7)), 137.83 (1C, s, ***C***(7a)), 138.12 (1C, s, ***C***(4)), 139.09 (1C, s, ***C***(6)), 142.09 (1C, s, ***C***(3a)), 145.98 (1C, s, ***C***(2)).

^{1}H-NMR: (400 MHz, DMSO-d_6, 25 °C) of compound **11**

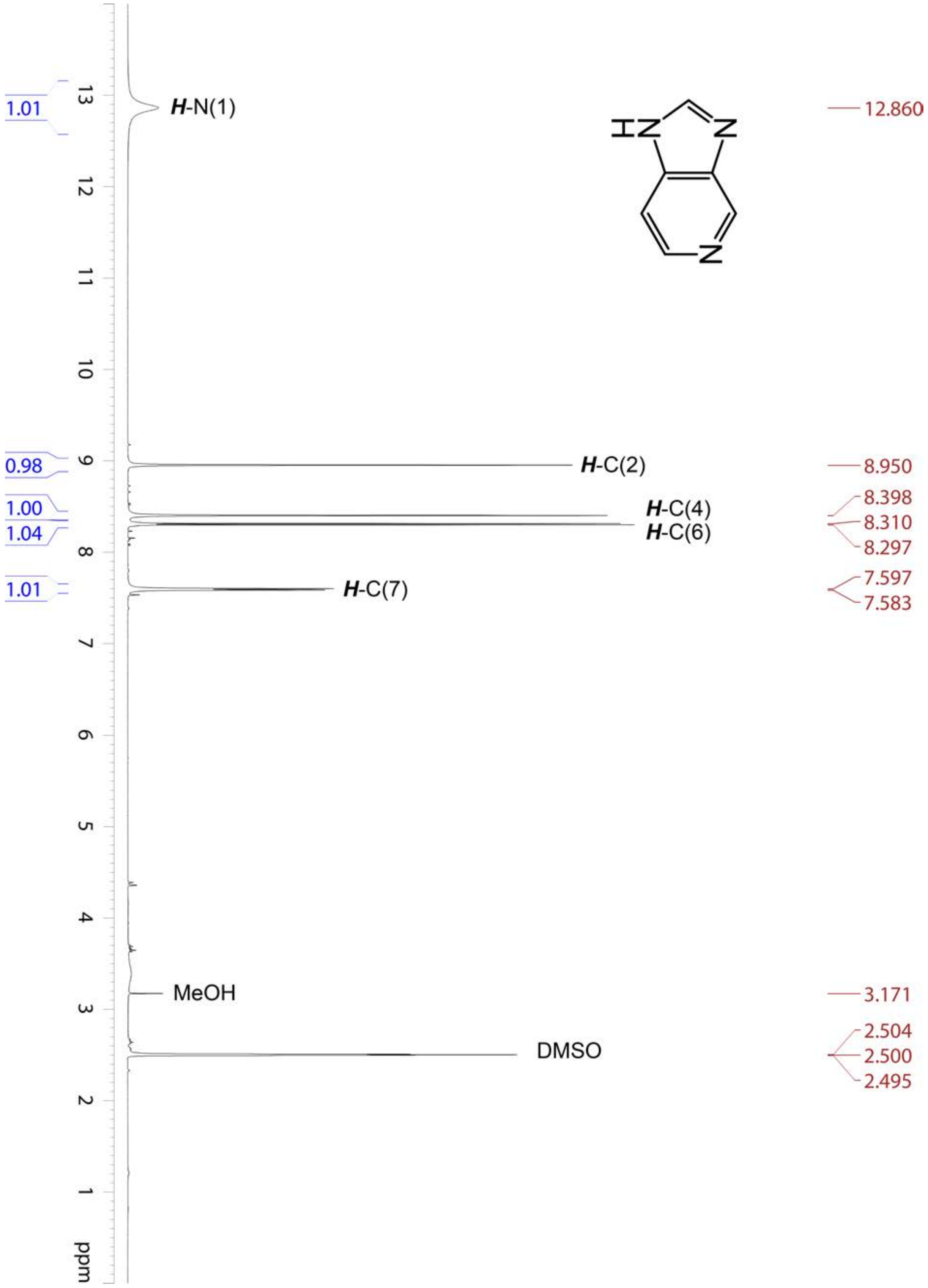

^{13}C-NMR: (100 MHz, DMSO-d_6 + 3% HCl (5 %), 25 °C) of compound **11**

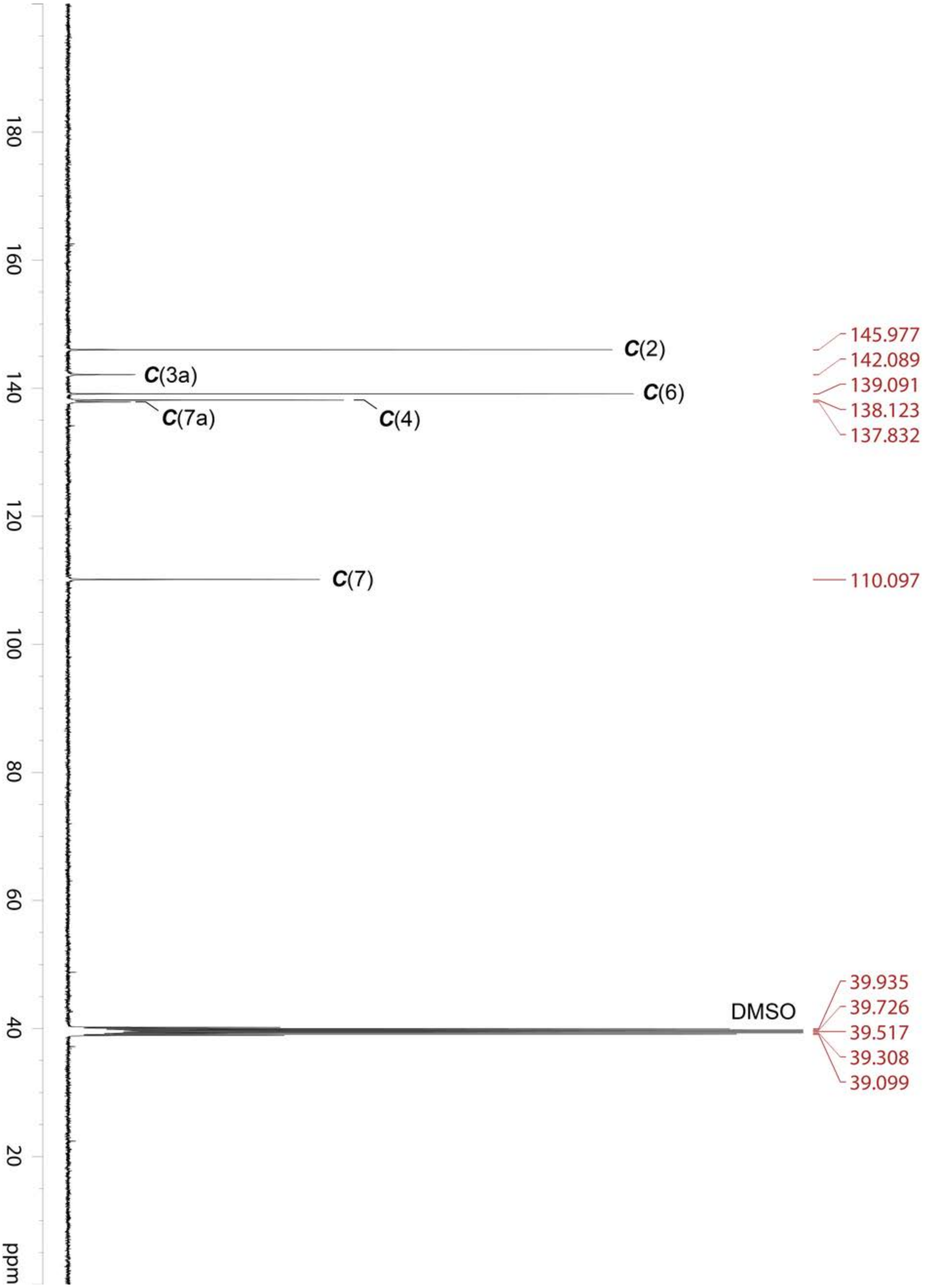

1*H*-Imidazo[4,5-c]pyridine-5-oxide (12)

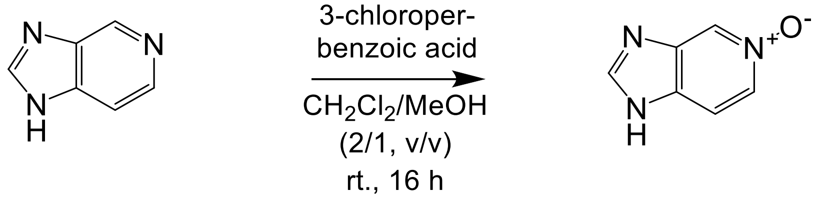

11 **12**

Compound **11** (5.25 g, 44.1 mmol) was dissolved in dichloromethane/methanol (2/1, v/v, 113 mL), treated with 3-chloroperbenzoic acid (21.7 g, 70 %, 88.1 mmol) and stirred at ambient temperatures for 16 hours. Upon completion, methanol was added until the precipitate was dissolved and the solution was charged on silica gel. The crude product was purified by column chromatography on SiO_2 (10 % to 30 % methanol in dichloromethane). Yield: 5.73 g of compound **12** as white solid (96 %). TLC: (CH_2Cl_2 / MeOH, 4/1): R_F = 0.11. ESI-MS (m/z): $[M+H]^+$ calcd.: 136.0505; found: 136.0507. ^{1}H-NMR: (400 MHz, DMSO-d_6, 25 °C): δ 7.61 (1H, d, J=6.88 Hz, ***H***-C(7)), 8.02 (1H, d, J=6.88 Hz, ***H***-C(6)), 8.41 (1H, s, ***H***-C(4)), 8.65 (1H, s, ***H***-C(2)). ^{13}C-NMR: (100 MHz, H_2O/D_2O (9/1) + 3% DMSO +3% HCl (5 %), 25 °C): δ 112.60 (1C, s, ***C***(7)), 130.59 (1C, s, ***C***(2)), 134.66 (1C, s, ***C***(6)), 137.50 (1C, s, ***C***(3a)), 138.34 (1C, s, ***C***(7a)), 149.09 (1C, s, ***C***(4)).

^{1}H-NMR: (400 MHz, DMSO-d$_6$, 25 °C) of compound **12**

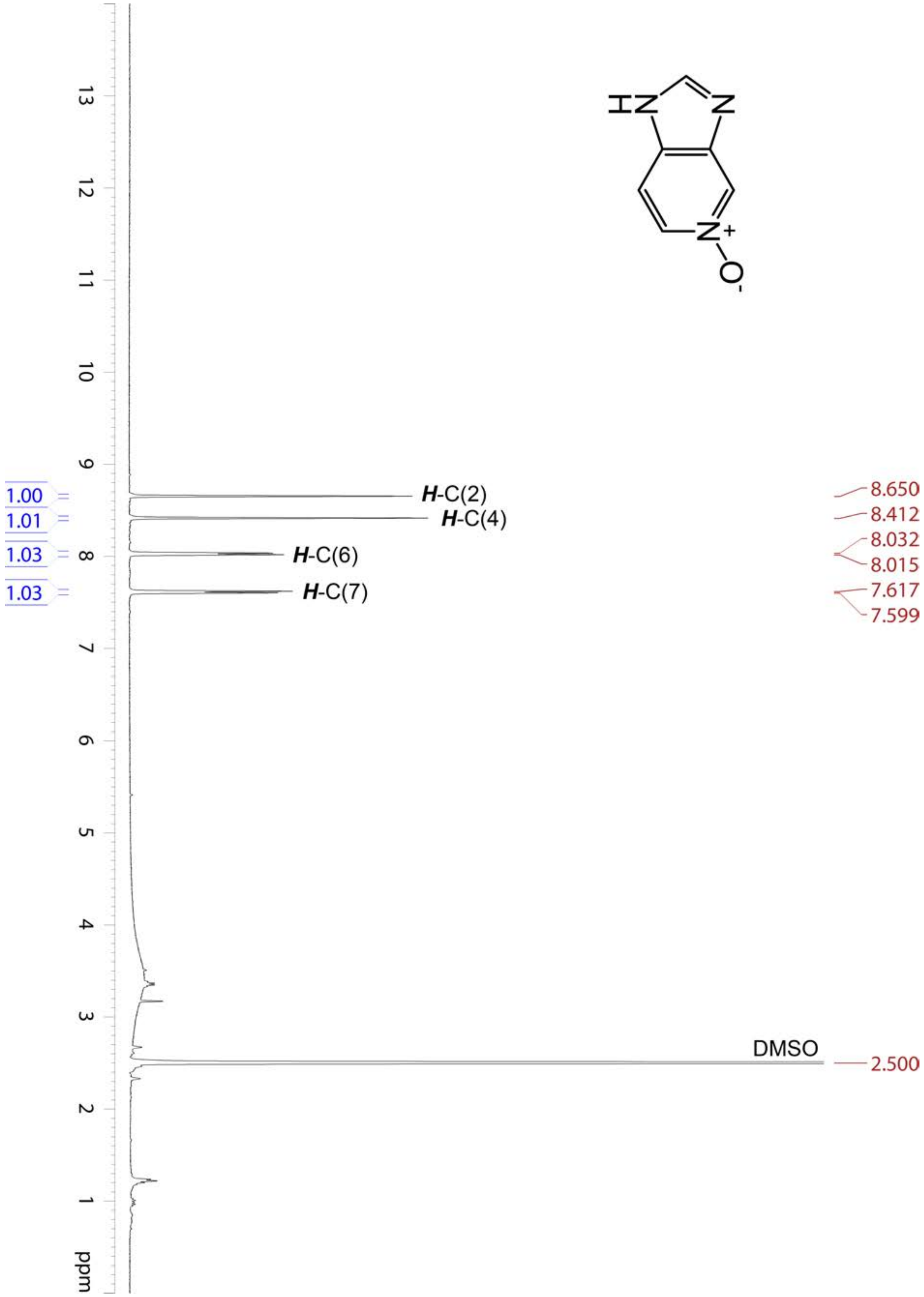

^{13}C-NMR: (100 MHz, H_2O/D_2O (9/1) + 3% DMSO +3% HCl (5 %), 25 °C) of compound **12**

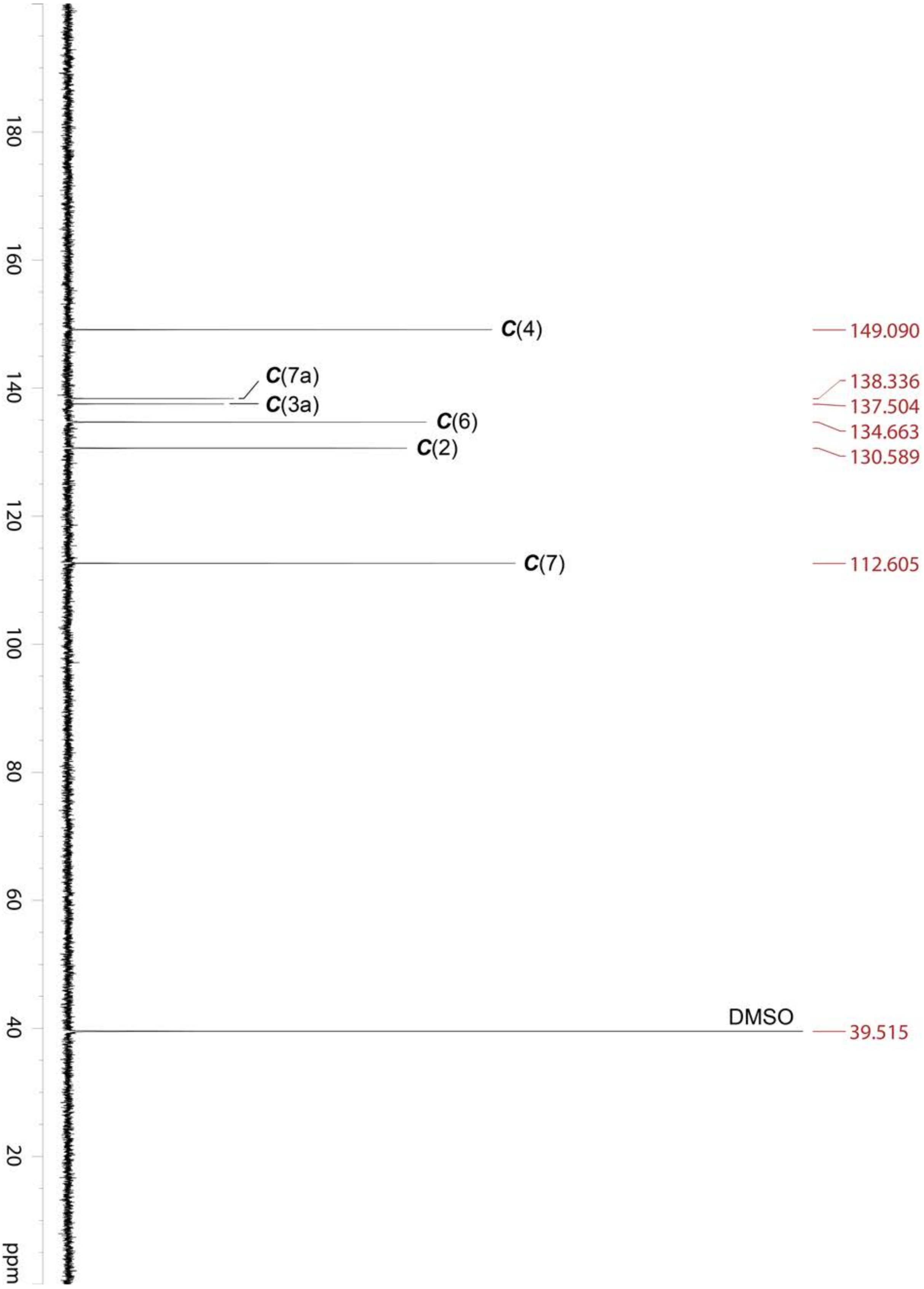

4-Chloro-1*H*-Imidazo[4,5-c]pyridine (13)

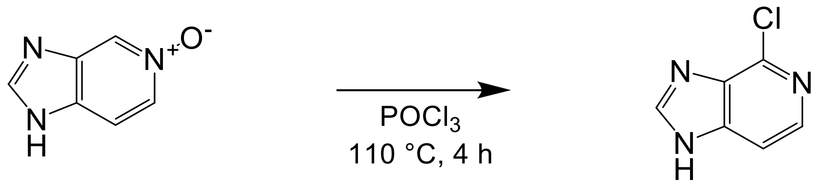

12 **13**

Compound **12** (5.55 g, 41.1 mmol) was dissolved in phosphorous oxychloride (154 mL) and stirred at 110 °C for four hours. Upon completion, solvents were evaporated, aqueous ammonia was added (10 mL) and residues were loaded onto silica gel. The crude product was purified by column chromatography on SiO_2 (2 % to 8 % methanol in dichloromethane). Yield: 4.20 g of compound **13** as grey solid (67 %). TLC: (CH_2Cl_2 / MeOH, 9/1): R_F = 0.62. ESI-MS (m/z): $[M+H]^+$ calcd.: 154.0167; found: 154.0164. ^{1}H-NMR: (400 MHz, DMSO-d_6, 25 °C): δ 7.61 (1H, s, ***H***-C(7)), 8.11 (1H, d, J=5.51 Hz, ***H***-C(6)), 8.46 (1H, s, ***H***-C(2)), 13.17 (1H, b, ***H***-N(1)). ^{13}C-NMR: (100 MHz, DMSO-d_6 + 3% HCl (5 %), 25 °C): δ 109.02 (1C, s, ***C***(7)), 134.96 (1C, s, ***C***(3a)), 139.41 (1C, s, ***C***(4)), 141.02 (1C, s, ***C***(7a)), 141.06 (1C, s, ***C***(2)), 144.77 (1C, s, ***C***(6)).

^{1}H-NMR: (400 MHz, DMSO-d$_6$, 25 °C) of compound **13**

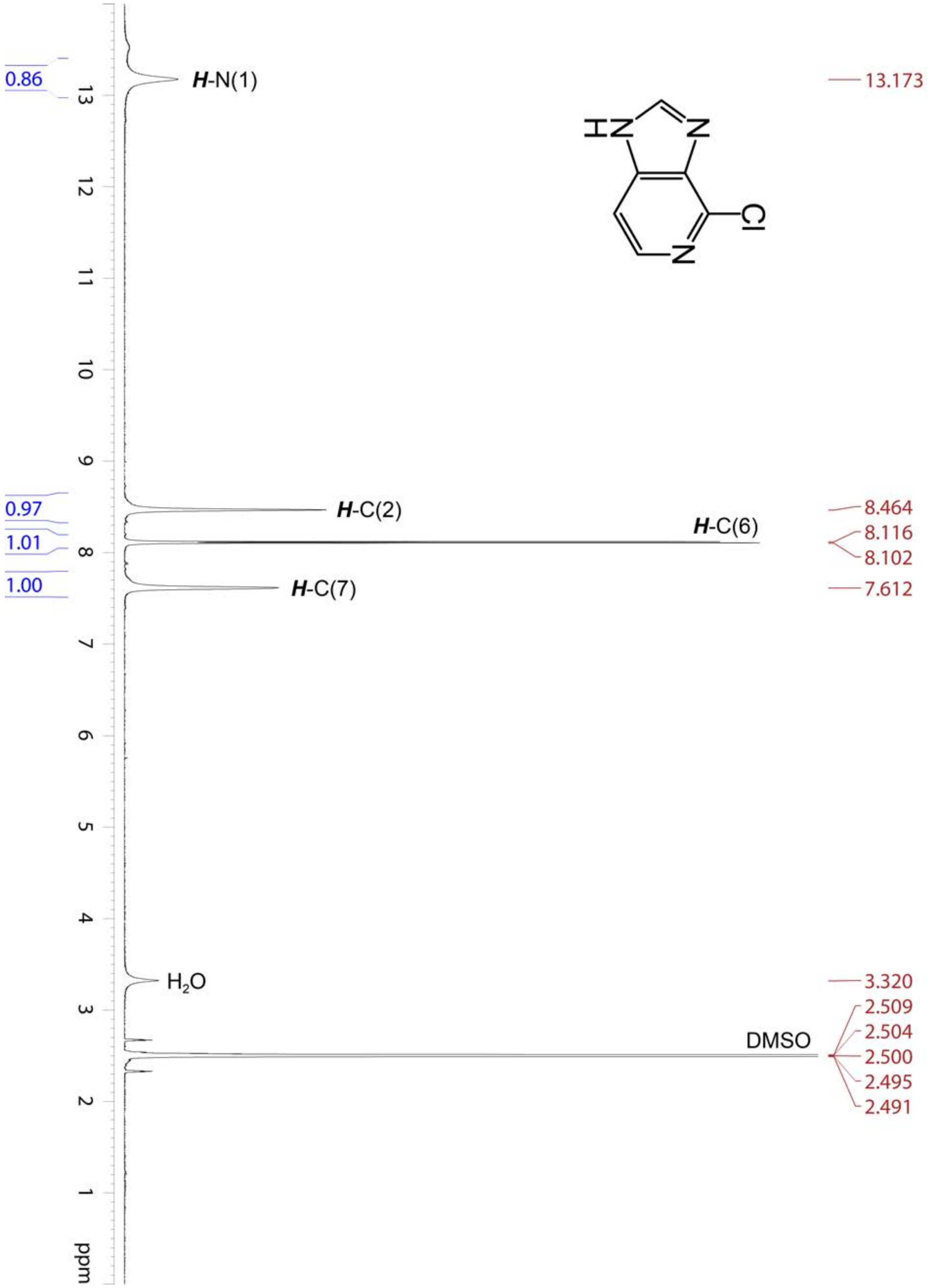

^{13}C-NMR: (100 MHz, DMSO-d_6 + 3% HCl (5 %), 25 °C) of compound **13**

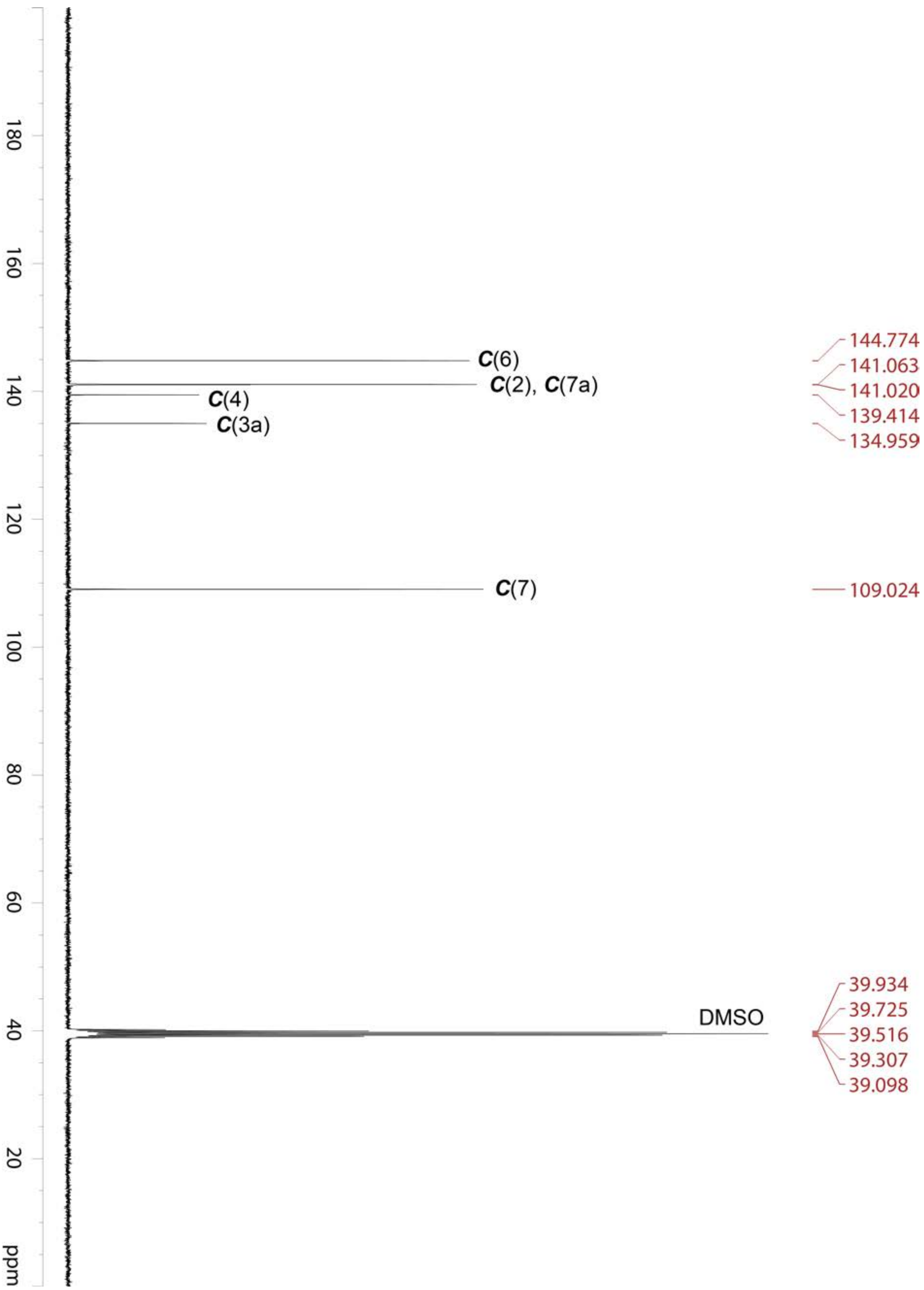

1*H*-Imidazo[4,5-c]pyridine-4-amine (14)

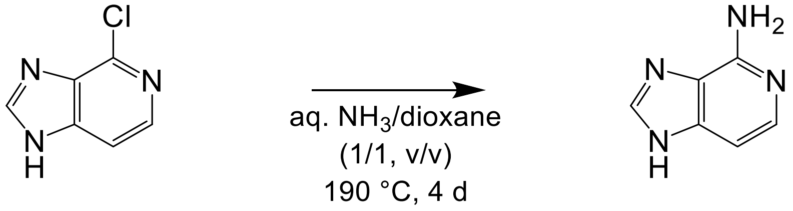

13 **14**

Compound **13** (3.00 g, 19.5 mmol) was dissolved in aqueous ammonia (33 %, 50 mL) and 1,4-dioxane (50 mL) and heated to 190 °C for 4 days in an autoclave. Upon completion, solvents were evaporated to dryness, residues were dissolved in boiling ethanol, allowed to cool to room temperature and filtered. The filtrate was concentrated and purified by column chromatography on SiO_2 (10 % to 25 % methanol in dichloromethane). Yield: 2.27 g of compound **14** as a white solid (87%). TLC: (CH_2Cl_2 / MeOH, 4/1): R_F = 0.22. ESI-MS (m/z): $[M+H]^+$ calcd.: 135.0665; found: 135.0664. ^{1}H-NMR: (400 MHz, DMSO-d_6, 25 °C): δ 7.02 (1H, s, ***H***-C(7)), 7.66 (1H, d, J=4.93 Hz, ***H***-C(6)), 7.85 (2H, s, ***H₂***N-C(4)), 8.35 (1H, s, ***H***-C(2)), 13.27 (1H, b, ***H***-N(1)). ^{13}C-NMR: (100 MHz, H_2O/D_2O (9/1) + 3% DMSO +3% HCl (5 %), 25 °C): δ 101.32 (1C, s, ***C***(7)), 125.29 (1C, s, ***C***(3a)), 129.59 (1C, s, ***C***(6)), 140.52 (1C, s, ***C***(7a)), 144.25 (1C, s, ***C***(2)), 148.43 (1C, s, ***C***(4)).

^{1}H-NMR: (400 MHz, DMSO-d$_6$, 25 °C) of compound **14**

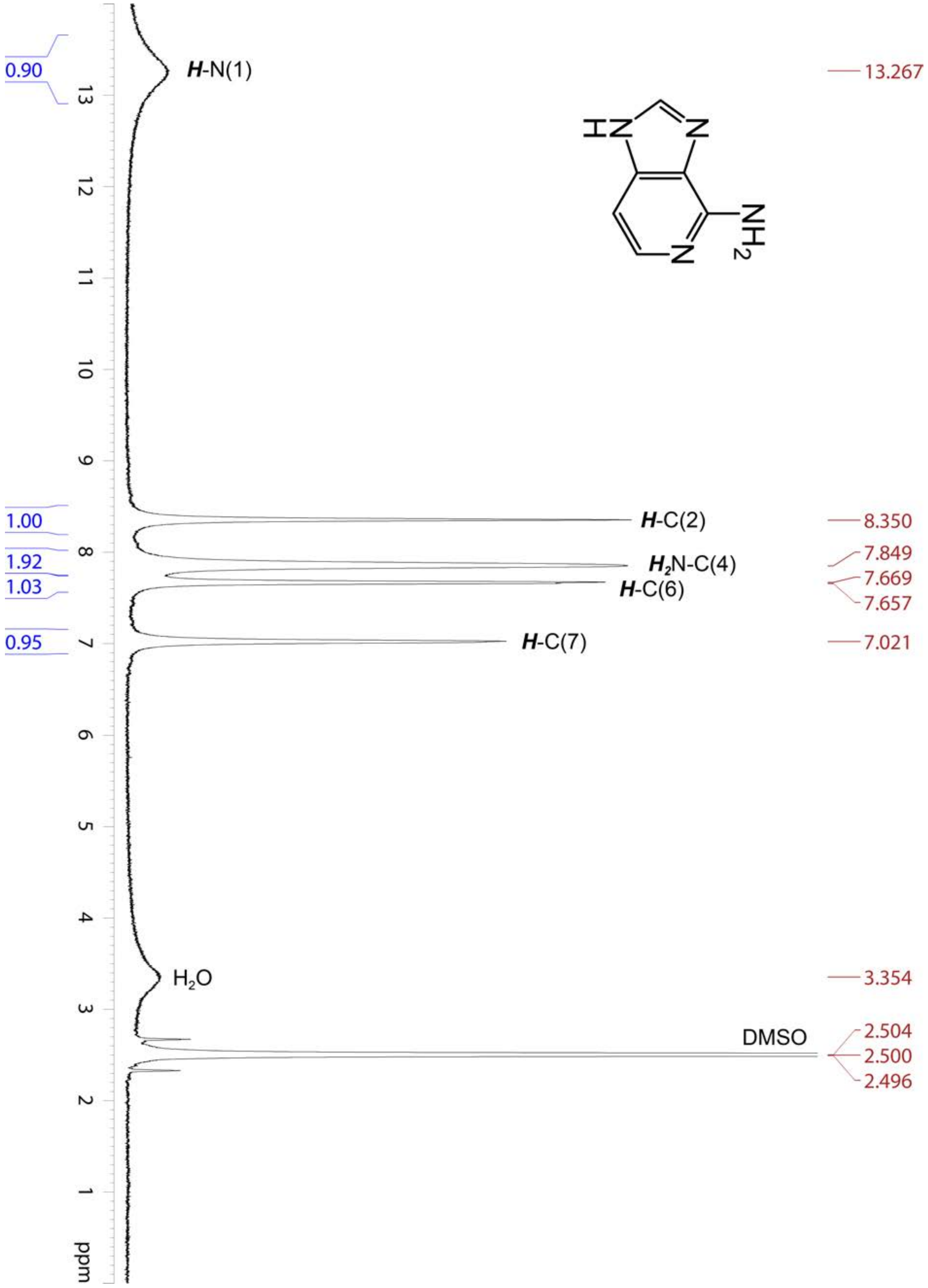

^{13}C-NMR: (100 MHz, H_2O/D_2O (9/1) + 3% DMSO + 3% HCl (5 %), 25 °C) of compound **14**

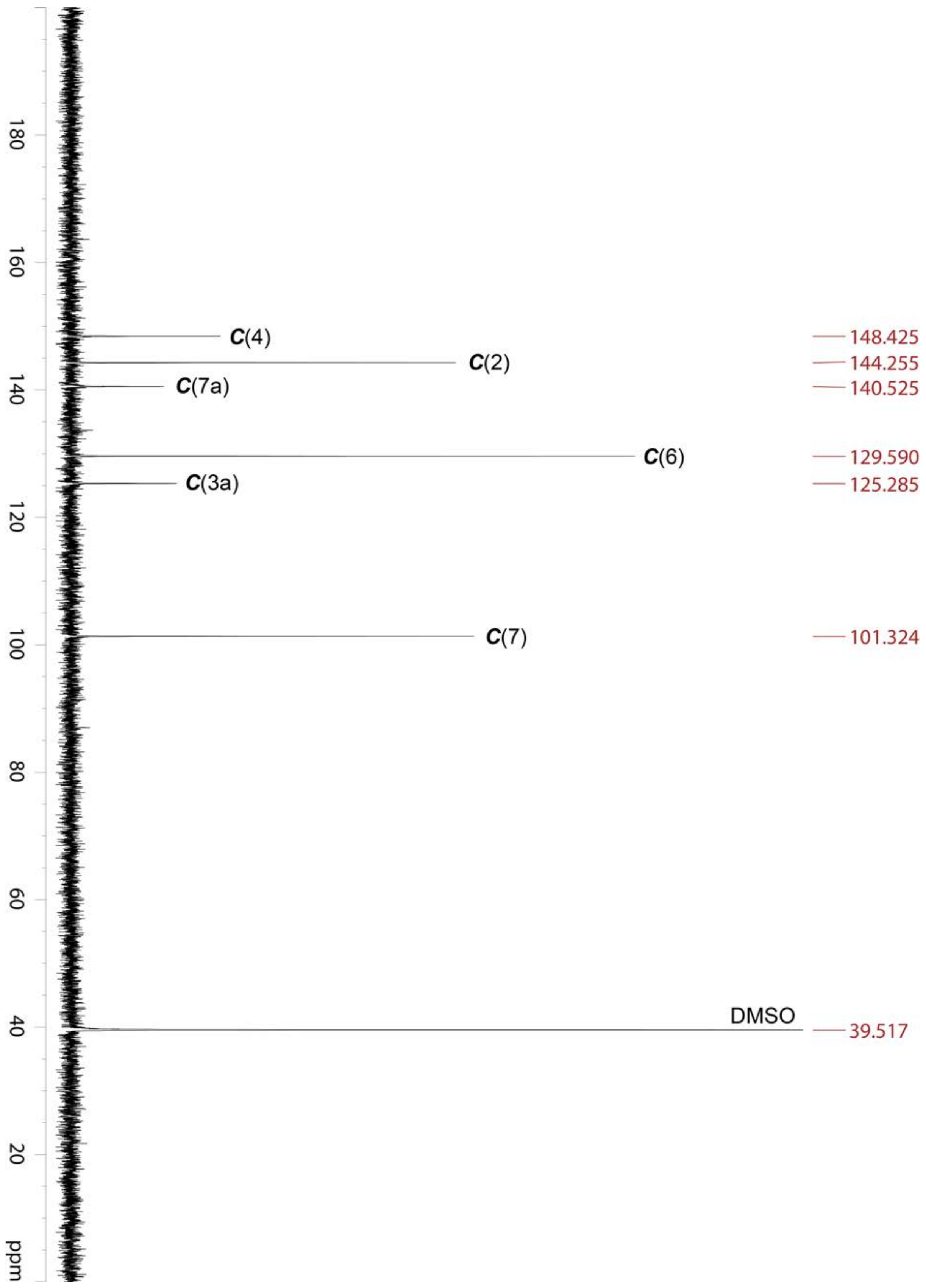

Supporting Table 1. Mass spectrometric analysis of unmodified and modified RNAs.

Sequence (5' → 3')	nt	Molecular weight	
		calc.	found
GGUCGACC (**III**)	8	2524.59	2524.59
GGUCc^3GACC (**IIIa**)	8	2523.60	2523.89
GGUCc^7GACC (**IIIb**)	8	2523.60	2523.76
GGUCGc^3ACC (**IIIc**)	8	2523.60	2523.65
GGUCGc^7ACC (**IIId**)	8	2523.60	2523.24
GGCUAGCC (**III'**)	8	2524.59	2524.82
GGCUAc^3GCC (**III'a**)	8	2523.60	2523.82
GGCUAc^7GCC (**III'b**)	8	2523.60	2523.23
GGCUc^3AGCC (**III'c**)	8	2523.60	2523.35
GGCUc^7AGCC (**III'd**)	8	2523.60	2523.65
GGCAGAGGC (**I**)	9	2932.87	2932.88
GCCUCUGCC (**I**)	9	2766.70	2766.88
GGCAc^3GAGGC (**Ia**)	9	2931.88	2932.02
GGCAc^7GAGGC (**Ib**)	9	2931.88	2931.88
GGCAGc^3AGGC (**Ic**)	9	2931.88	2931.63
GGCAGc^7AGGC (**Id**)	9	2931.88	2932.10
GAAGGGCAACCUUCG (**II**)	15	4813.99	4813.55
GAAc^3GGGCAACCUUCG (**IIa**)	15	4813.00	4813.43
GAAc^7GGGCAACCUUCG (**IIb**)	15	4813.00	4813.42
GAc^3AGGGCAACCUUCG (**IIc**)	15	4813.00	4813.55
GAc^7AGGGCAACCUUCG (**IId**)	15	4813.00	4813.22
ACCCc^3GCAAGGCCGACGGC	18	5766.43	5766.49
UGCUCCUAGUACGAGAGGACCGc^3GAGUG	27	8726.34	8727.72
UGCUCCUAc^3GUACGAGAGGACCGGAGUG	27	8726.34	8726.67
UGCUCCUAGUACGAGAGGACCGGc^3AGUG	27	8726.34	8728.10
CGUGGUUAGGGCCACGUUAAAUAGU<br>UGCUUAAGCCCUAAc^3GCGUUGAU	47	15077.96	15077.98

Supporting Table 2. Top-down FT-ICR mass spectrometric sequence analysis of c^3G and c^3A modified RNAs.

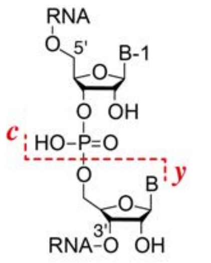

MS data from CAD of $(M - 4H)^{4-}$ ions of GGCUAc^3GCC:

m/z_{exp}	charge	m_{exp} [Da]	assignment	m_{calc} [Da]	Δm [Da]
689.0877	1-	690.0950	c_2	690.0949	0.0001
994.1273	1-	995.1345	c_3	995.1362	-0.0016
649.5737	2-	1301.1619	c_4	1301.1615	0.0005
814.0990	2-	1630.2125	c_5	1630.2140	-0.0015
657.0819	3-	1974.2674	c_6	1974.2662	0.0013
758.7618	3-	2279.3071	c_7	2279.3074	-0.0004
629.5914	4-	2522.3945	M	2522.3930	0.0015
547.1197	1-	548.1270	y_2	548.1268	0.0001
891.1709	1-	892.1782	y_3	892.1790	-0.0008
609.6089	2-	1221.2324	y_4	1221.2315	0.0009
762.6219	2-	1527.2583	y_5	1527.2568	0.0015
609.7589	3-	1832.2985	y_6	1832.2981	0.0004
724.7745	3-	2177.3452	y_7	2177.3455	-0.0004

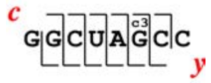

MS data from CAD of $(M - 4H)^{4-}$ ions of GGUCGc^3ACC:

m/z_{exp}	charge	m_{exp} [Da]	assignment	m_{calc} [Da]	Δm [Da]
344.0396	1-	345.0469	c_1	345.0474	-0.0005
689.0874	1-	690.0947	c_2	690.0949	-0.0002
995.1127	1-	996.1200	c_3	996.1202	-0.0002
649.5737	2-	1301.1619	c_4	1301.1615	0.0004
547.7291	3-	1646.2092	c_5	1646.2089	0.0003
657.0817	3-	1974.2670	c_6	1974.2662	0.0008
758.7615	3-	2279.3062	c_7	2279.3074	-0.0012
629.5908	4-	2522.3925	M	2522.3930	-0.0005
547.1196	1-	548.1268	y_2	548.1268	0.0000
437.0848	2-	876.1841	y_3	876.1841	0.0000
609.6088	2-	1221.2321	y_4	1221.2315	0.0006
762.1292	2-	1526.2730	y_5	1526.2728	0.0002
915.1416	2-	1832.2978	y_6	1832.2981	-0.0003
724.7754	3-	2177.3481	y_7	2177.3455	0.0026

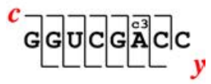

Supporting Table 3. X-ray data collection and crystallographic refinement statistics.

		SRL-C[3]A2670
PDB ID		7L3R
Space group		$P4_3$
a (Å)		29.54
b (Å)		29.54
c (Å)		76.30
Beamline		SLS PX I - X06SA
Resolution range (Å)		50.0 – 1.0
Number of frames		3600
Oscillation angle		0.1°
Wavelength (Å)		0.82
Average redundancy		6.8
Completeness [1]		99.9% (99.8%)
CC1/2 [1]		99.9 (80.9)
Average I/σ [1]		19.1 (1.9)
ISa		21.6
R/R_{free}		12.7 / 14.6
Coordinate error (Å)		0.14
Phase Error (°)		15.49
R.M.S. deviations		
Bond length (Å)		0.009
Bond angles (°)		1.298
Molprobity Clash Score		3.0
Coordinate error (Å)		0.13
Atoms	RNA	620
	Water	168
	Ligand	28
Mean B (Å²)	RNA	15.3
	Water	31.8
	Ions	34.0

[1] Values for last resolution shell are shown in parenthesis

I)

5' GC 3'
GC
GC
CG
AU
GC
AU
GC
GC
3' CG 5'

concentration / µM	T_m(duplex) / °C
1.8	61.3
4.8	64.0
11.6	66.6
35.2	70.6
88.6	72.2

ΔG^0 kcal mol^{-1}	ΔH^0 kcal mol^{-1}	ΔS^0 cal mol^{-1} K^{-1}
-16.4	-78.8	-209.5

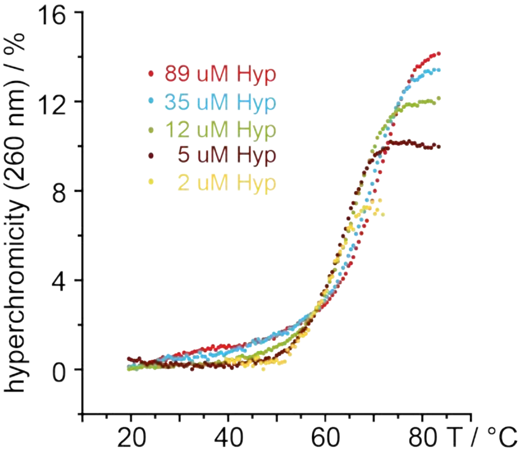

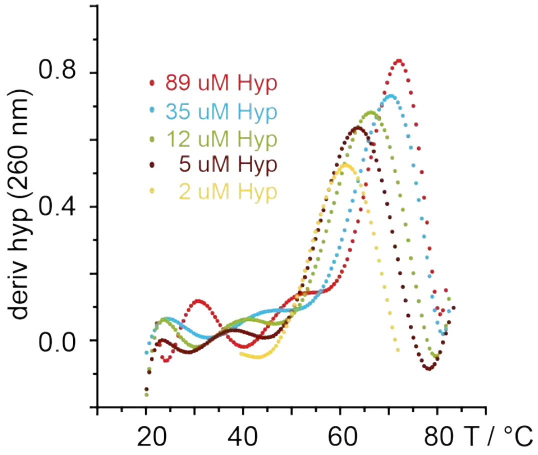

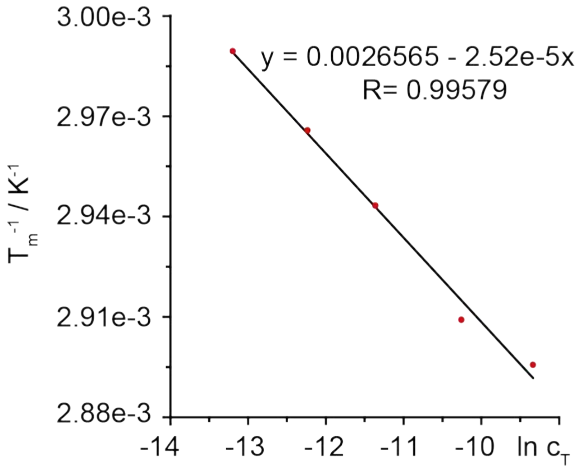

Supporting Figure 1. Thermodynamic analysis of RNA base pairing. Non-modified 9 bp duplex **I**: Sequence and secondary structure, summary of RNA concentrations, T_m values, and thermodynamic parameters (top left); graph illustrating the superposition of UV-melting profiles (top right), graph illustrating the superposition of the first derivatives of the melting curves (bottom left), and ln c versus 1/T plot (bottom right). Conditions: 10 mM Na_2HPO_4, 150 mM NaCl, pH 7.0.

Ia)

5' GC 3'
GC
CG
AU
c^3GC
AU
GC
GC
CG
3' 5'

concentration / µM	T_m(duplex) / °C
2.3	58.1
5.7	61.0
13.6	63.0
42.4	67.1

ΔG^0 kcal mol^{-1}	ΔH^0 kcal mol^{-1}	ΔS^0 cal mol^{-1} K^{-1}
-15.1	-74.3	-198.6

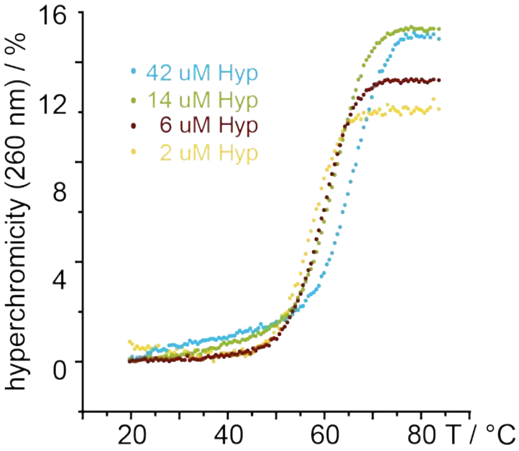

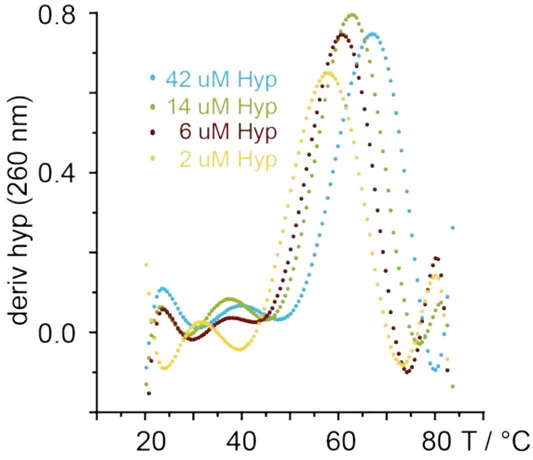

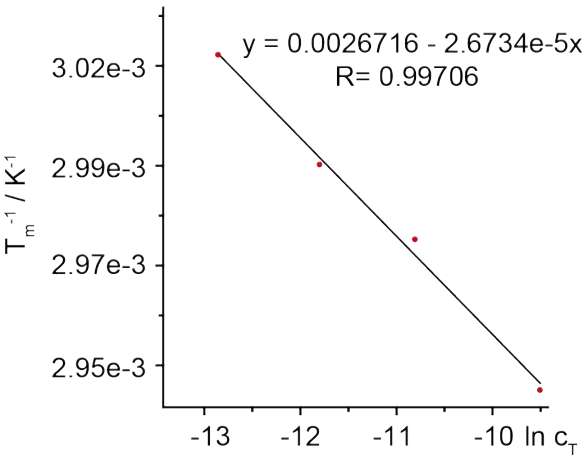

Supporting Figure 2. Thermodynamic analysis of RNA base pairing. c^3G-modified 9 bp duplex **Ia**: Sequence and secondary structure, summary of RNA concentrations, T_m values, and thermodynamic parameters (top left); graph illustrating the superposition of UV-melting profiles (top right), graph illustrating the superposition of the first derivatives of the melting curves (bottom left), and ln c versus 1/T plot (bottom right). Conditions: 10 mM Na_2HPO_4, 150 mM NaCl, pH 7.0.

Ib)

5' 3'
G C
G C
C G
A U
G C
c^3A U
G C
G C
C G
3' 5'

concentration / µM	T_m(duplex) / °C
2.2	59.2
5.2	61.8
12.5	63.5
38.2	67.7
100.9	69.8

ΔG^0 kcal mol^{-1}	ΔH^0 kcal mol^{-1}	ΔS^0 cal mol^{-1} K^{-1}
-16.0	-80.3	-215.9

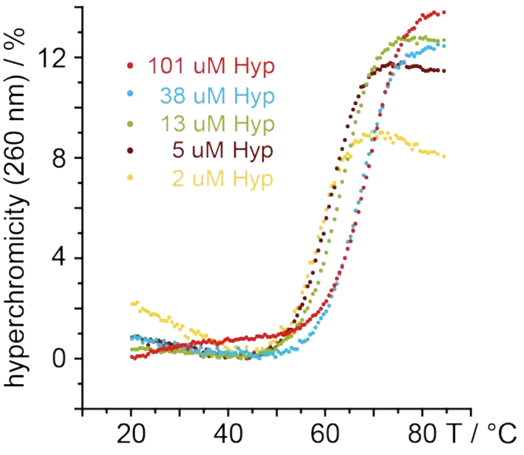

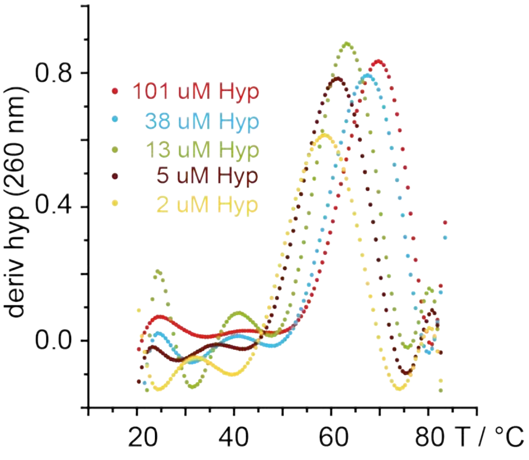

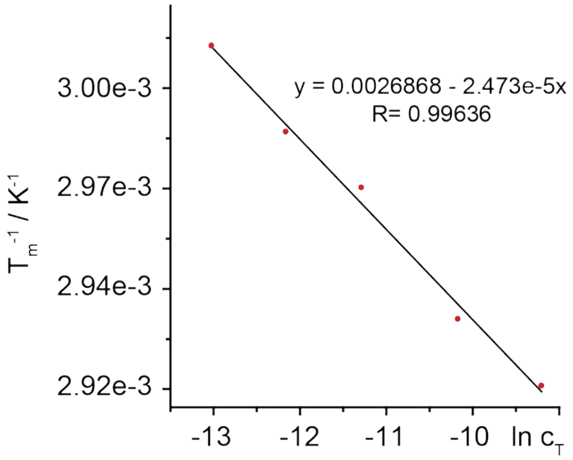

Supporting Figure 3. Thermodynamic analysis of RNA base pairing. c^3A-modified 9 bp duplex **Ib**: Sequence and secondary structure, summary of RNA concentrations, T_m values, and thermodynamic parameters (top left); graph illustrating the superposition of UV-melting profiles (top right), graph illustrating the superposition of the first derivatives of the melting curves (bottom left), and ln c versus 1/T plot (bottom right). Conditions: 10 mM Na_2HPO_4, 150 mM NaCl, pH 7.0.

Ic)

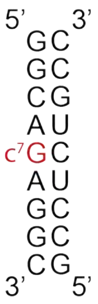

concentration / µM	T_m(duplex) / °C
2.1	62.3
5.6	64.6
13.4	67.2
42.1	71.0
104.5	73.1

ΔG^0 kcal mol^{-1}	ΔH^0 kcal mol^{-1}	ΔS^0 cal mol^{-1} K^{-1}
-16.7	-80.5	-214.1

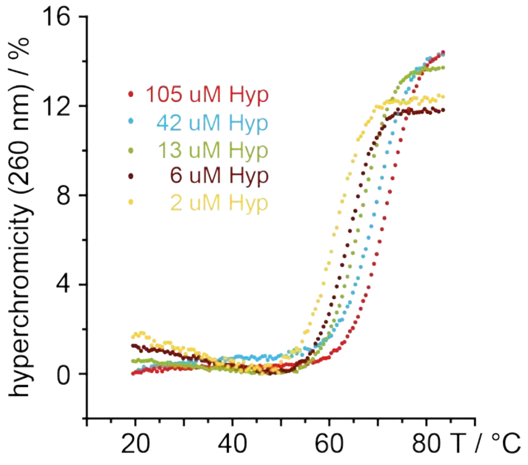

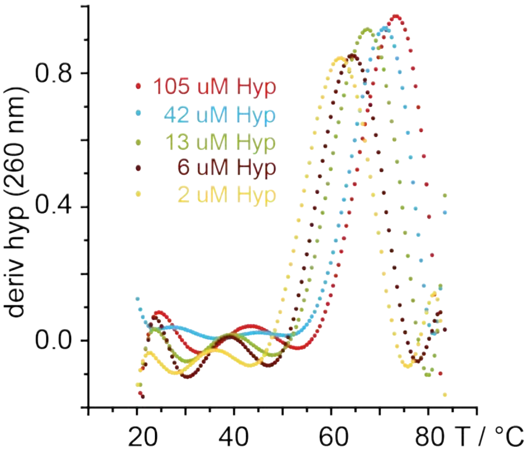

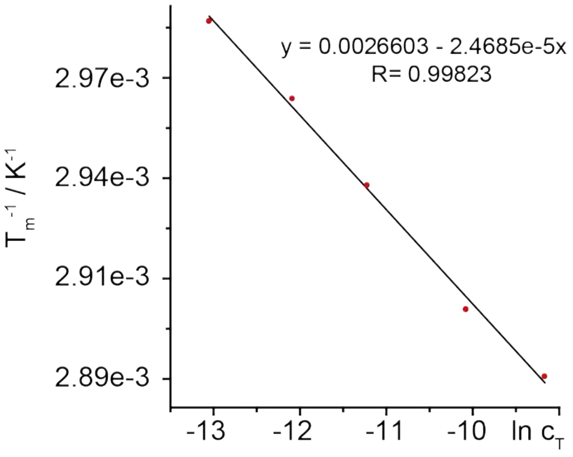

Supporting Figure 4. Thermodynamic analysis of RNA base pairing. c^7G-modified 9 bp oligoribonucleotide **Ic**: Sequence and secondary structure, summary of RNA concentrations, T_m values, and thermodynamic parameters (top left); graph illustrating the superposition of UV-melting profiles (top right), graph illustrating the superposition of the first derivatives of the melting curves (bottom left), and ln c versus 1/T plot (bottom right). Conditions: 10 mM Na_2HPO_4, 150 mM NaCl, pH 7.0.

Id)

5' G C 3'
G C
C G
A U
G C
c^7A U
G C
G C
3' C G 5'

concentration / µM	T_m(duplex) / °C
2.0	61.6
4.9	63.5
11.1	66.1
34.9	70.4
84.6	72.1

ΔG^0 kcal mol^{-1}	ΔH^0 kcal mol^{-1}	ΔS^0 cal mol^{-1} K^{-1}
-16.2	-77.4	-205.3

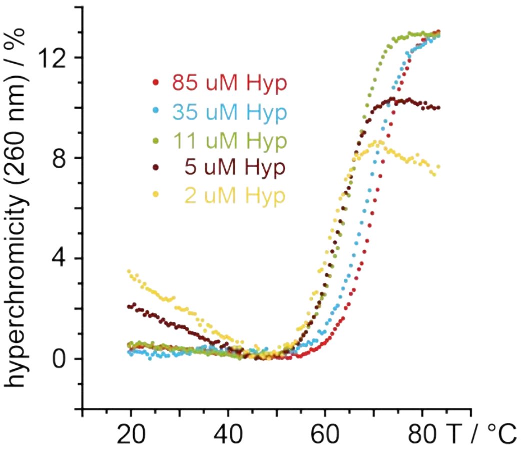

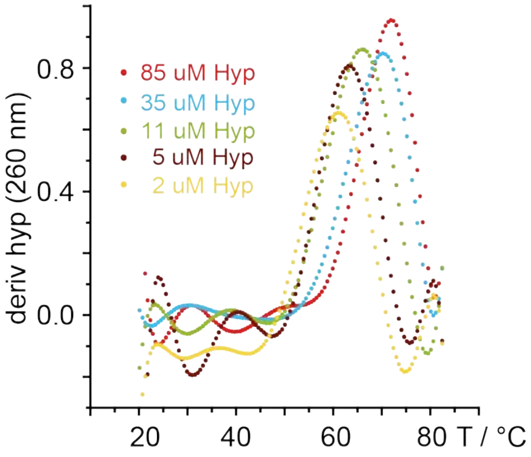

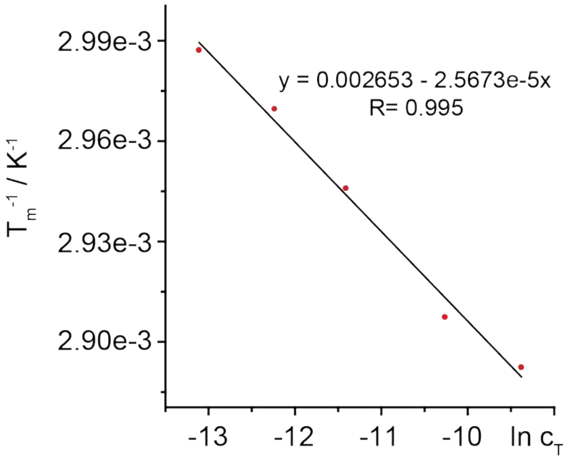

Supporting Figure 5. Thermodynamic analysis of RNA base pairing. c^7A-modified 9 bp duplex **Id**: Sequence and secondary structure, summary of RNA concentrations, T_m values, and thermodynamic parameters (top left); graph illustrating the superposition of UV-melting profiles (top right), graph illustrating the superposition of the first derivatives of the melting curves (bottom left), and ln c versus 1/T plot (bottom right). Conditions: 10 mM Na_2HPO_4, 150 mM NaCl, pH 7.0.

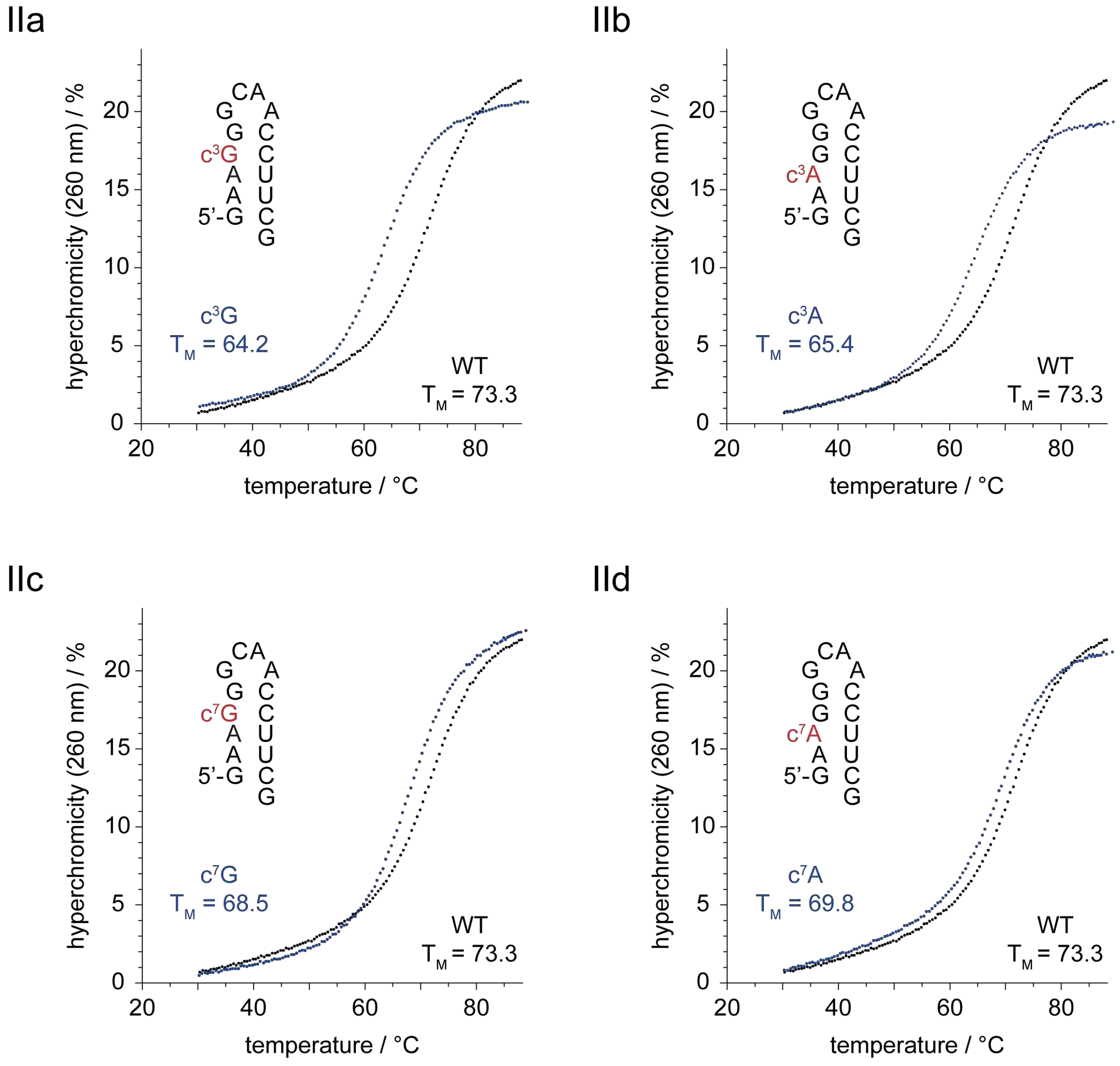

Supporting Figure 6. UV melting profile comparisons of (**IIa**) 3-deazaadenosine-, (**IIb**) 3-deazaguanosine- (**IIc**), 7-deazaadenosine- (**IId**) and 7-deazaguanosine-modifed 15 nt RNA hairpins and unmodifed reference RNA (wild-type, WT). Conditions: c_{RNA} = 8 µM; 10 mM Na_2HPO_4, 150 mM NaCl, in H_2O; pH = 7.0.

III)

5' 3'
GC
GC
UA
CG
GC
AU
CG
CG
3' 5'

concentration / µM	T_m(duplex) / °C
2.4	53.2
5.2	55.6
12.5	58.2
40.8	62.4
99.9	65.4

ΔG^0 kcal mol^{-1}	ΔH^0 kcal mol^{-1}	ΔS^0 cal mol^{-1} K^{-1}
-13.4	-66.8	-179.1

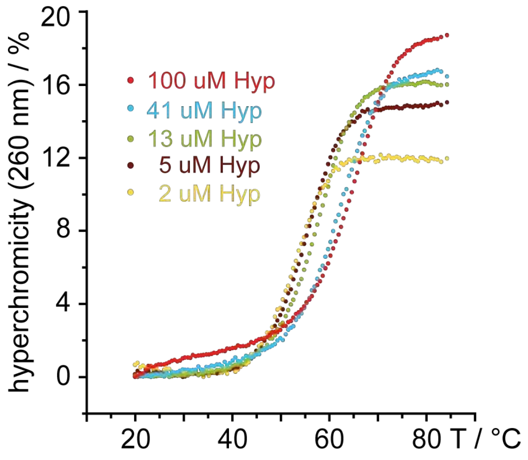

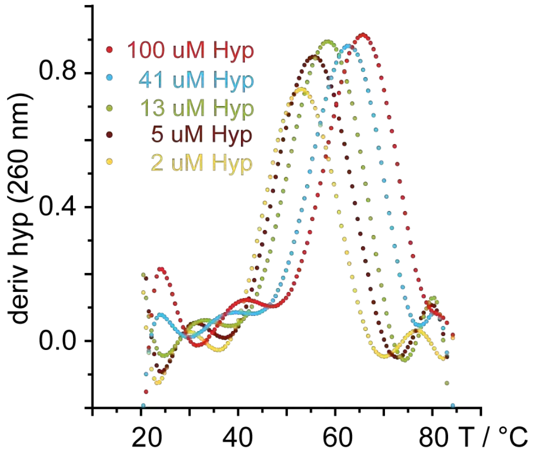

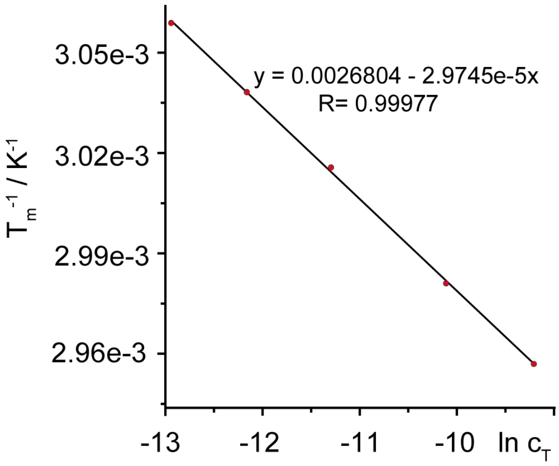

Supporting Figure 7. Thermodynamic analysis of RNA base pairing. Non-modified 8 nt oligoribonucleotide **III**: Sequence and secondary structure, summary of RNA concentrations, T_m values, and thermodynamic parameters (top left); graph illustrating the superposition of UV-melting profiles (top right), graph illustrating the superposition of the first derivatives of the melting curves (bottom left), and ln c versus 1/T plot (bottom right). Conditions: 10 mM Na_2HPO_4, 150 mM NaCl, pH 7.0.

IIIa)

5' 3'
G C
G C
U A
C c3G
c3G C
A U
C G
C G
3' 5'

concentration / µM	T_m(duplex) / °C
1.7	39.6
4.5	42.2
10.8	44.8
33.8	47.9
53.2	49.6

ΔG^0 kcal mol^{-1}	ΔH^0 kcal mol^{-1}	ΔS^0 cal mol^{-1} K^{-1}
-11.1	-69.9	-197.2

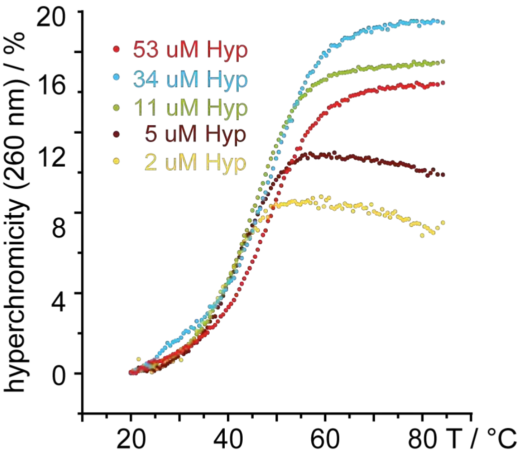

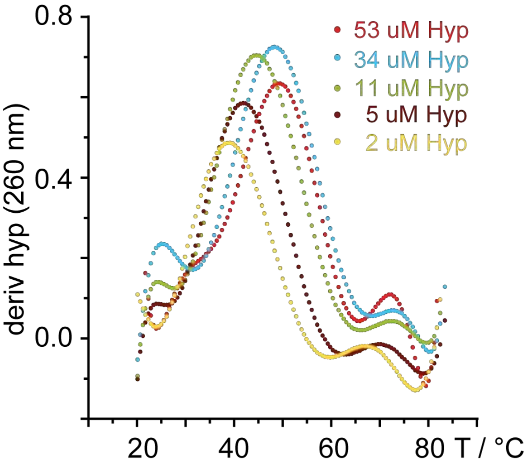

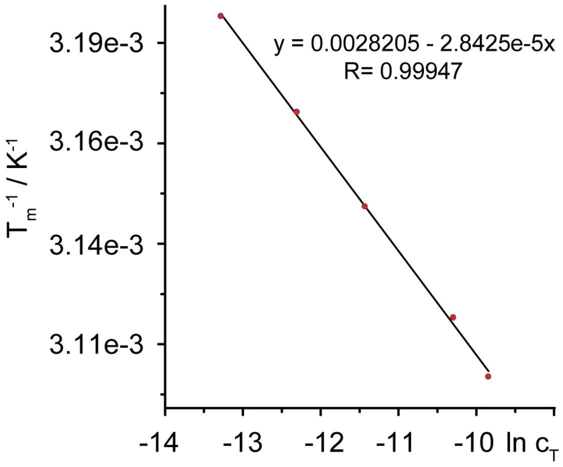

Supporting Figure 8. Thermodynamic analysis of RNA base pairing. c^{3}G-modified 8 nt oligoribonucleotide **IIIa**: Sequence and secondary structure, summary of RNA concentrations, T_m values, and thermodynamic parameters (top left); graph illustrating the superposition of UV-melting profiles (top right), graph illustrating the superposition of the first derivatives of the melting curves (bottom left), and ln c versus 1/T plot (bottom right). Conditions: 10 mM Na_2HPO_4, 150 mM NaCl, pH 7.0.

IIIb)

5' 3'
G C
G C
U c^{3}A
C G
G C
c^{3}A U
C G
C G
3' 5'

concentration / µM	T_m(duplex) / °C
2.1	43.7
5.1	47.1
12.2	49.2
35.7	52.2
92.4	54.7

ΔG^0 kcal mol^{-1}	ΔH^0 kcal mol^{-1}	ΔS^0 cal mol^{-1} K^{-1}
-12.2	-73.0	-204.1

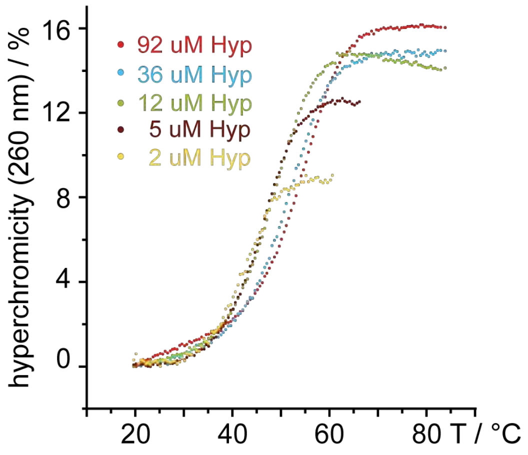

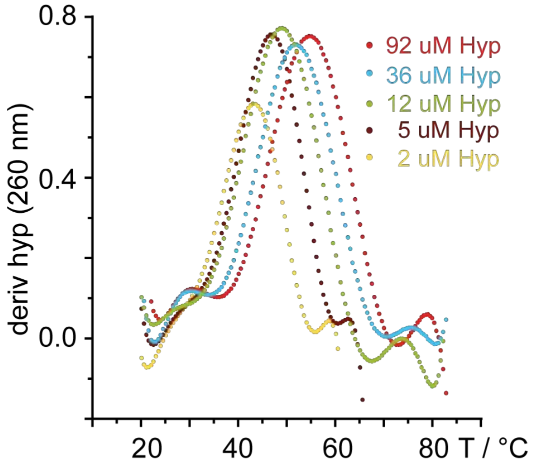

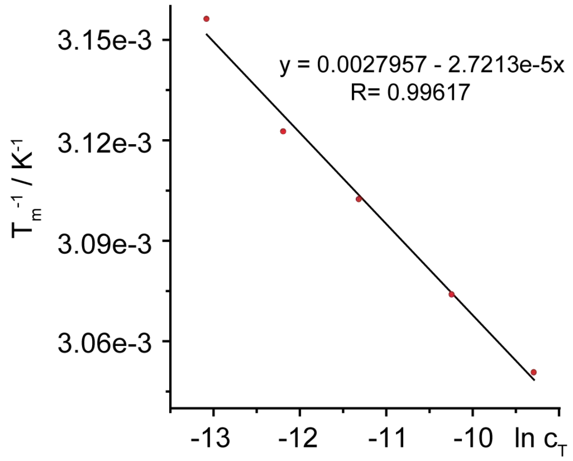

Supporting Figure 9. Thermodynamic analysis of RNA base pairing. c^{3}A-modified 8 nt oligoribonucleotide **IIIb**: Sequence and secondary structure, summary of RNA concentrations, T_m values, and thermodynamic parameters (top left); graph illustrating the superposition of UV-melting profiles (top right), graph illustrating the superposition of the first derivatives of the melting curves (bottom left), and ln c versus 1/T plot (bottom right). Conditions: 10 mM Na_2HPO_4, 150 mM NaCl, pH 7.0.

IIIc)

5' G C 3'
G C
U A
C c7G
c7G C
A U
C G
C G
3' 5'

concentration / µM	T_m(duplex) / °C
1.8	50.6
4.7	53.1
11.2	55.7
34.4	59.7
85.3	62.1

ΔG^0 kcal mol^{-1}	ΔH^0 kcal mol^{-1}	ΔS^0 cal mol^{-1} K^{-1}
-13.4	-70.9	-192.8

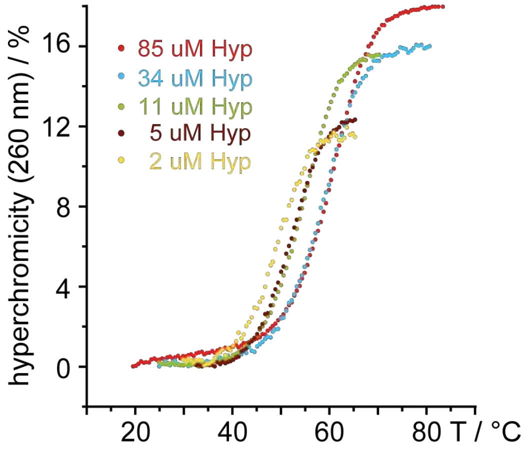

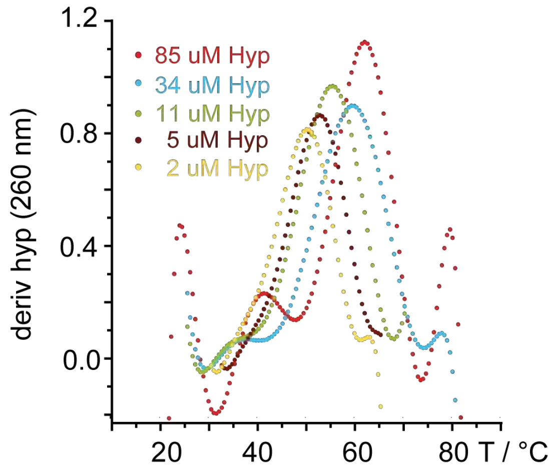

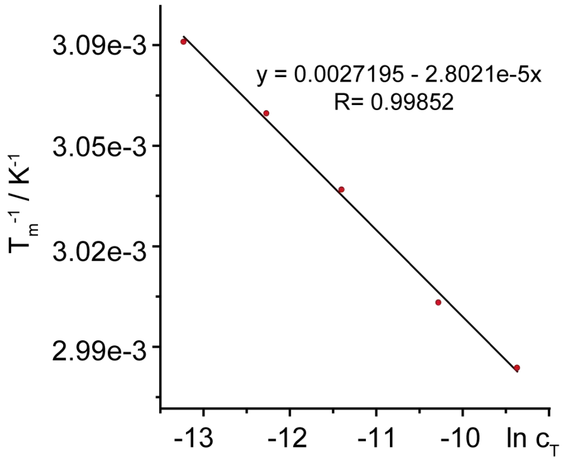

Supporting Figure 10. Thermodynamic analysis of RNA base pairing. c^7G-modified 8 nt oligoribonucleotide **IIIc**: Sequence and secondary structure, summary of RNA concentrations, T_m values, and thermodynamic parameters (top left); graph illustrating the superposition of UV-melting profiles (top right), graph illustrating the superposition of the first derivatives of the melting curves (bottom left), and ln c versus 1/T plot (bottom right). Conditions: 10 mM Na_2HPO_4, 150 mM NaCl, pH 7.0.

IIId)

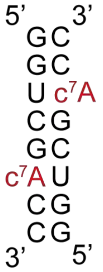

concentration / µM	T_m(duplex) / °C
1.7	49.5
4.4	52.6
10.3	54.8
31.2	58.8
78.4	61.7

ΔG^0 kcal mol^{-1}	ΔH^0 kcal mol^{-1}	ΔS^0 cal mol^{-1} K^{-1}
-13.0	-67.1	-181.6

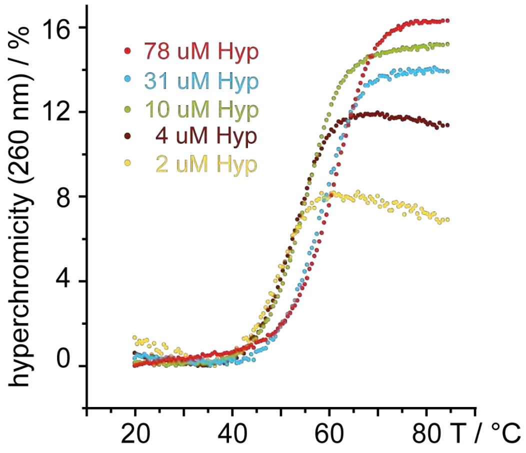

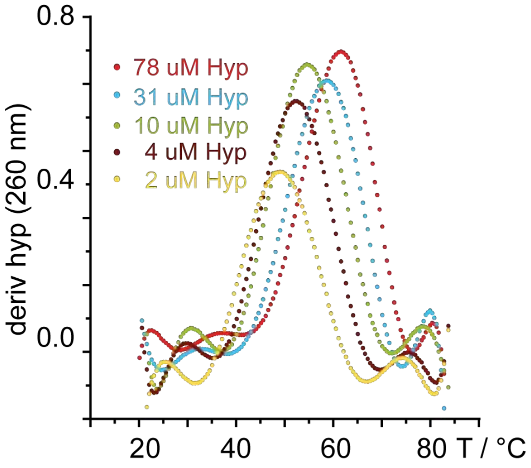

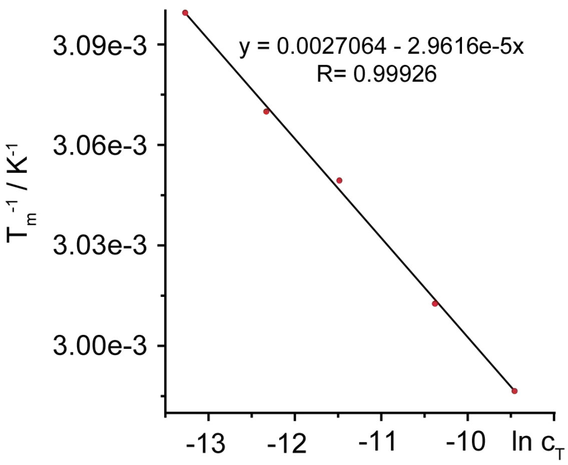

Supporting Figure 11. Thermodynamic analysis of RNA base pairing. c^7A-modified 8 nt oligoribonucleotide **IIId**: Sequence and secondary structure, summary of RNA concentrations, T_m values, and thermodynamic parameters (top left); graph illustrating the superposition of UV-melting profiles (top right), graph illustrating the superposition of the first derivatives of the melting curves (bottom left), and ln c versus 1/T plot (bottom right). Conditions: 10 mM Na_2HPO_4, 150 mM NaCl, pH 7.0.

III')

5' 3'
GC
GC
CG
UA
AU
GC
CG
CG
3' 5'

concentration / µM	T_m(duplex) / °C
2.3	55.9
4.7	58.0
10.7	60.3
33.2	63.8
65.0	65.9

ΔG^0 kcal mol^{-1}	ΔH^0 kcal mol^{-1}	ΔS^0 cal mol^{-1} K^{-1}
-14.6	-74.0	-199.0

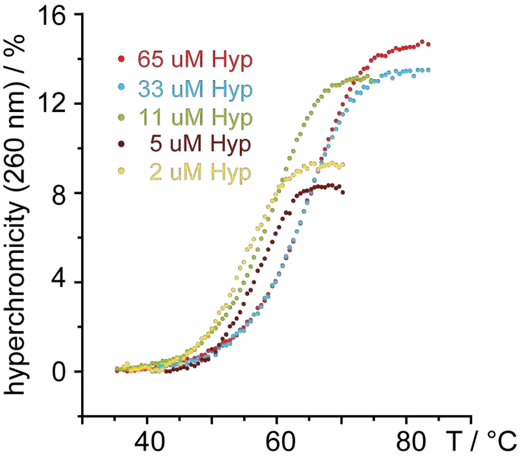

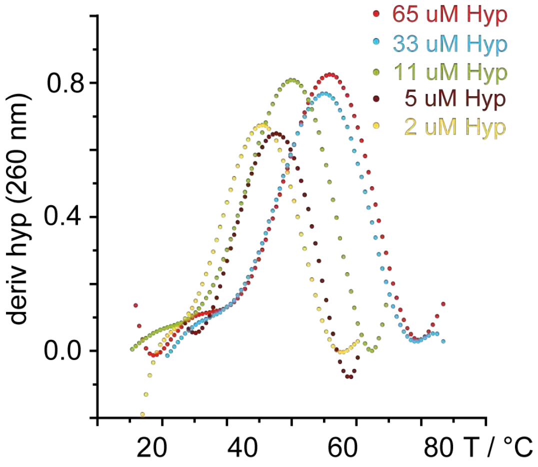

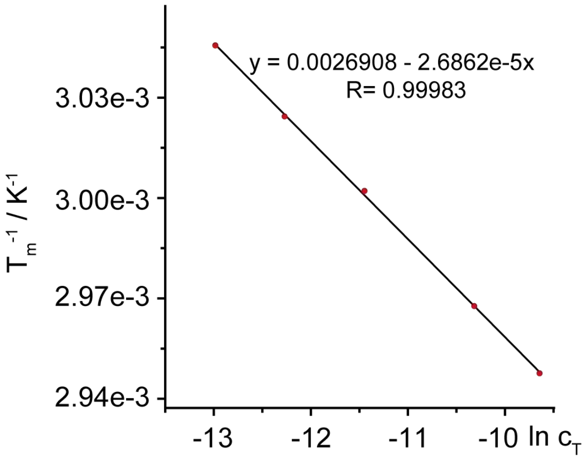

Supporting Figure 12. Thermodynamic analysis of RNA base pairing. Non-modified 8 nt oligoribonucleotide **III'**: Sequence and secondary structure, summary of RNA concentrations, T_m values, and thermodynamic parameters (top left); graph illustrating the superposition of UV-melting profiles (top right), graph illustrating the superposition of the first derivatives of the melting curves (bottom left), and ln c versus 1/T plot (bottom right). Conditions: 10 mM Na_2HPO_4, 150 mM NaCl, pH 7.0.

III'a)

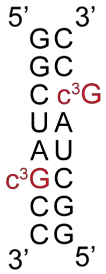

concentration / µM	T_m(duplex) / °C
1.9	43.2
4.3	45.3
7.6	47.0
32.6	50.7
83.6	53.6

ΔG^0 kcal mol^{-1}	ΔH^0 kcal mol^{-1}	ΔS^0 cal mol^{-1} K^{-1}
-12.1	-74.7	-210.0

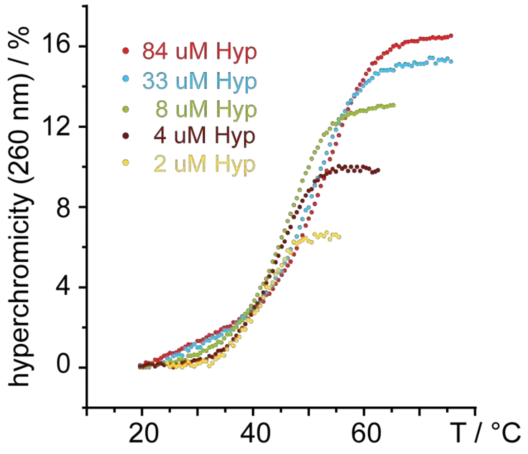

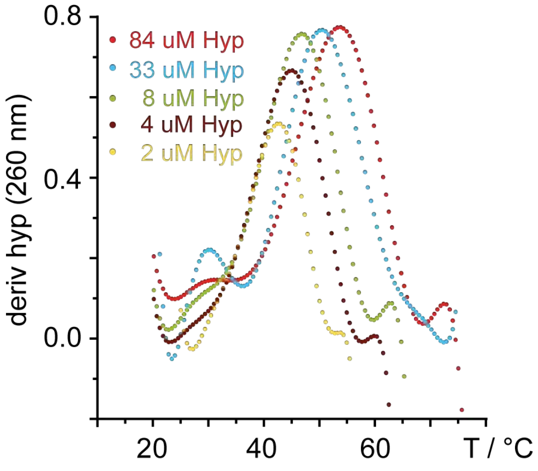

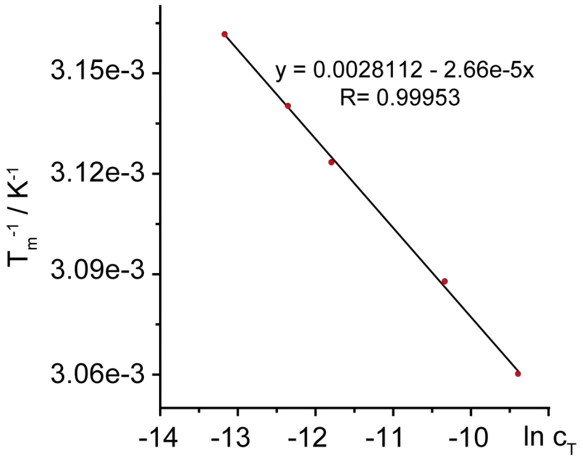

Supporting Figure 13. Thermodynamic analysis of RNA base pairing. c^3G-modified 8 nt oligoribonucleotide **III'a**: Sequence and secondary structure, summary of RNA concentrations, T_m values, and thermodynamic parameters (top left); graph illustrating the superposition of UV-melting profiles (top right), graph illustrating the superposition of the first derivatives of the melting curves (bottom left), and ln c versus 1/T plot (bottom right). Conditions: 10 mM Na_2HPO_4, 150 mM NaCl, pH 7.0.

III’b)

5’ 3’
GC
GC
CG
U c3A
c3A U
GC
CG
CG
3’ 5’

concentration / µM	T_m(duplex) / °C
2.2	41.1
5.3	44.8
12.4	48.1
39.2	52.3
88.7	54.9

ΔG^0 kcal mol^{-1}	ΔH^0 kcal mol^{-1}	ΔS^0 cal mol^{-1} K^{-1}
-10.9	-58.5	-159.6

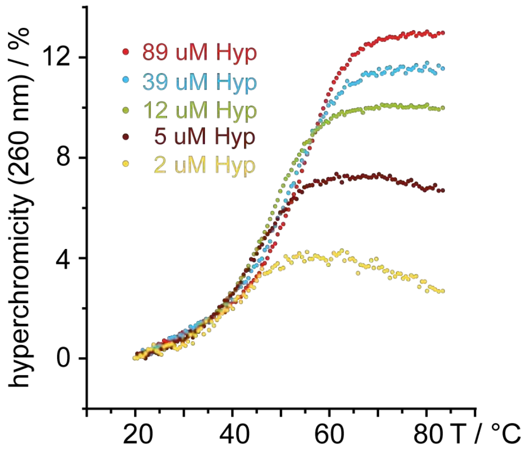

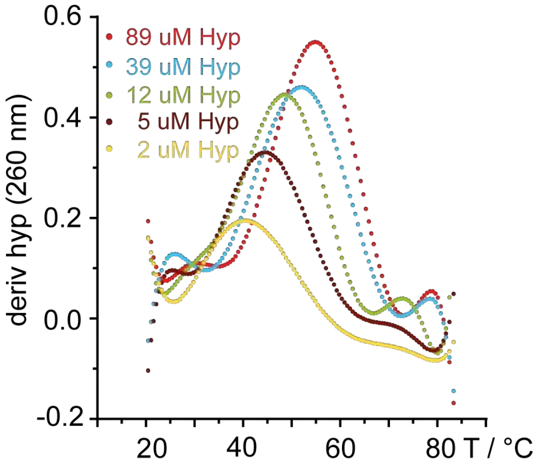

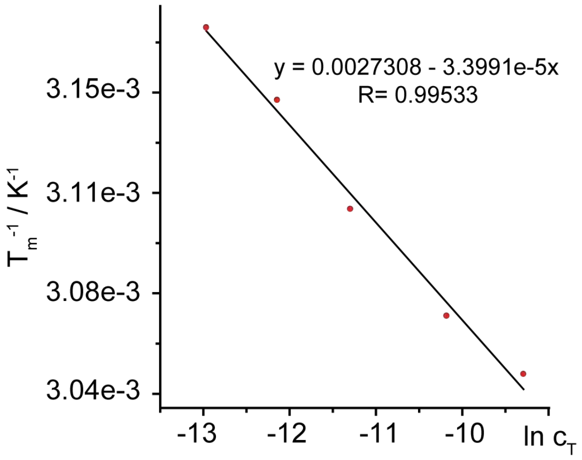

Supporting Figure 14. Thermodynamic analysis of RNA base pairing. c^{3}A-modified 8 nt oligoribonucleotide **III'b**: Sequence and secondary structure, summary of RNA concentrations, T_m values, and thermodynamic parameters (top left); graph illustrating the superposition of UV-melting profiles (top right), graph illustrating the superposition of the first derivatives of the melting curves (bottom left), and ln c versus 1/T plot (bottom right). Conditions: 10 mM Na_2HPO_4, 150 mM NaCl, pH 7.0.

III'c)

5' GC 3'
GC
C c⁷G
UA
AU
c⁷G C
CG
3' CG 5'

concentration / µM	T_m(duplex) / °C
2.0	53.6
4.5	56.4
10.7	58.5
32.7	62.0
82.9	64.6

ΔG^0 kcal mol^{-1}	ΔH^0 kcal mol^{-1}	ΔS^0 cal mol^{-1} K^{-1}
-14.4	-74.7	-202.6

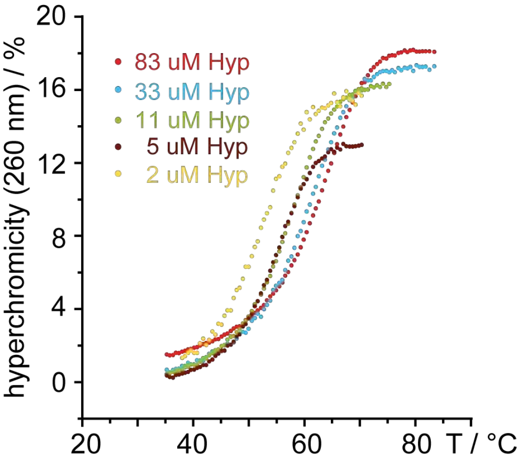

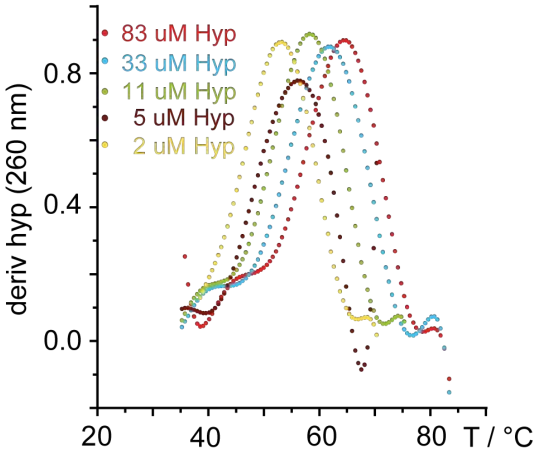

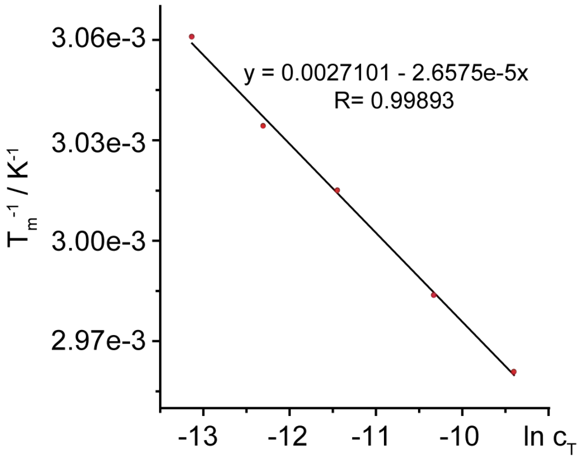

Supporting Figure 15. Thermodynamic analysis of RNA base pairing. c^7G-modified 8 nt oligoribonucleotide **III'c**: Sequence and secondary structure, summary of RNA concentrations, T_m values, and thermodynamic parameters (top left); graph illustrating the superposition of UV-melting profiles (top right), graph illustrating the superposition of the first derivatives of the melting curves (bottom left), and ln c versus 1/T plot (bottom right). Conditions: 10 mM Na_2HPO_4, 150 mM NaCl, pH 7.0.

III'd)

5' 3'
GC
GC
CG
U c7A
c7A U
GC
CG
CG
3' 5'

concentration / µM	T_m(duplex) / °C
2.1	54.6
5.0	57.0
11.6	59.6
35.9	63.3
89.2	65.8

ΔG^0 kcal mol^{-1}	ΔH^0 kcal mol^{-1}	ΔS^0 cal mol^{-1} K^{-1}
-14.3	-72.6	-195.4

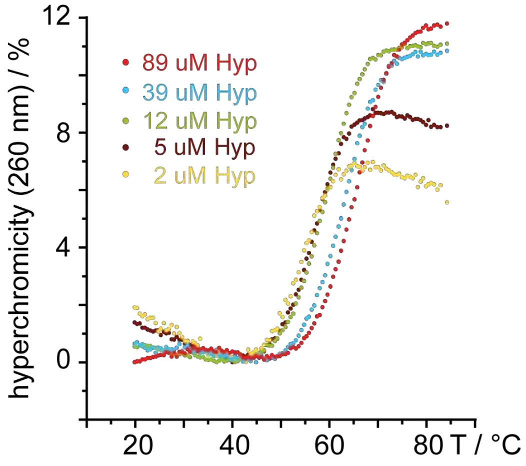

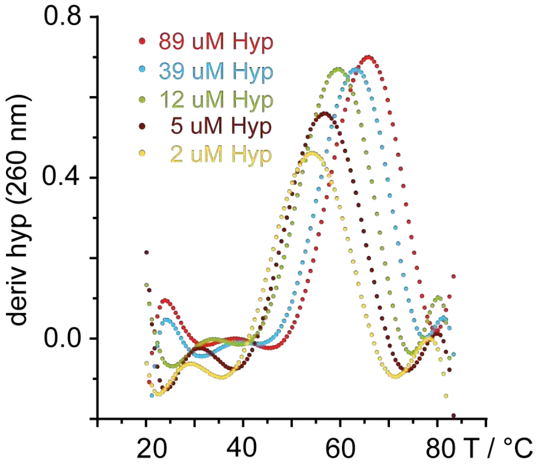

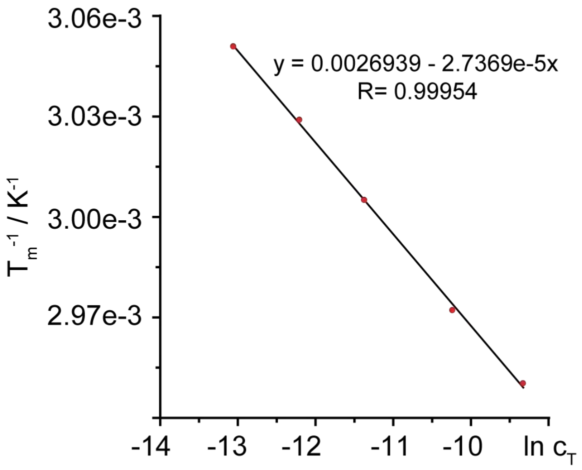

Supporting Figure 16. Thermodynamic analysis of RNA base pairing. c^7A-modified 8 nt oligoribonucleotide **III'd**: Sequence and secondary structure, summary of RNA concentrations, T_m values, and thermodynamic parameters (top left); graph illustrating the superposition of UV-melting profiles (top right), graph illustrating the superposition of the first derivatives of the melting curves (bottom left), and ln c versus 1/T plot (bottom right). Conditions: 10 mM Na_2HPO_4, 150 mM NaCl, pH 7.0.

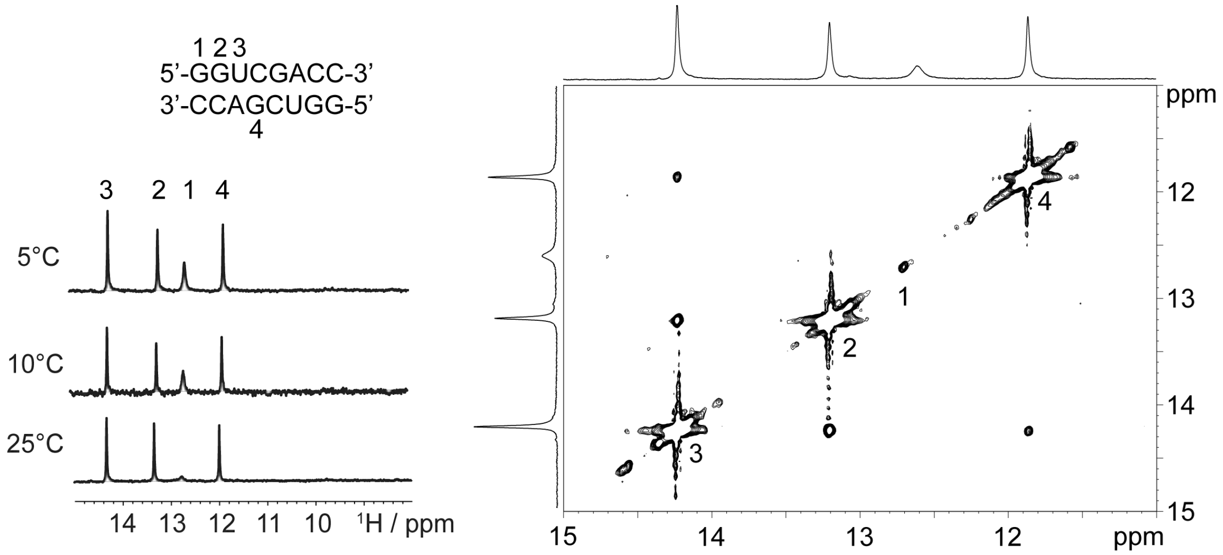

Supporting Figure 17. ^{1}H-NMR spectroscopic analysis of a palindromic 8 nt RNA. Sequence, secondary structure, and temperature-dependent spectra of the imino proton ppm region (left). ^{1}H,^{1}H-NOESY NMR spectrum used for peak assignment (right). Conditions: c(RNA) 0.5 mM; 15 mM $Na[AsO_2(CH_3)_2]\cdot 3H_2O$, 25 mM NaCl, 3 mM NaN_3, in H_2O/D_2O 9:1, pH 6.5, 298 K.

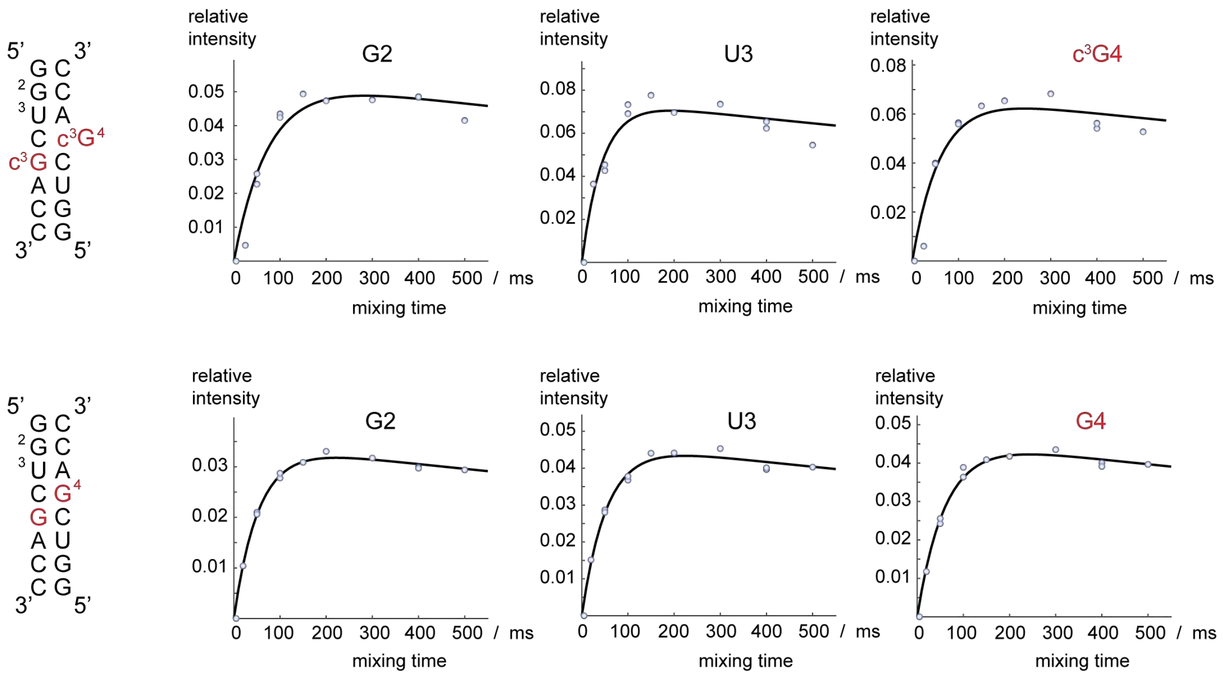

Supporting Figure 18. NMR spectroscopic determination of exchange rates of imino protons with the solvent. The plots depict the relative ^{1}H signal intensities versus mixing times from CLEANEX-PM experiments. The fits of the experimental data gave imino proton–bulk water exchange rates (see main text).

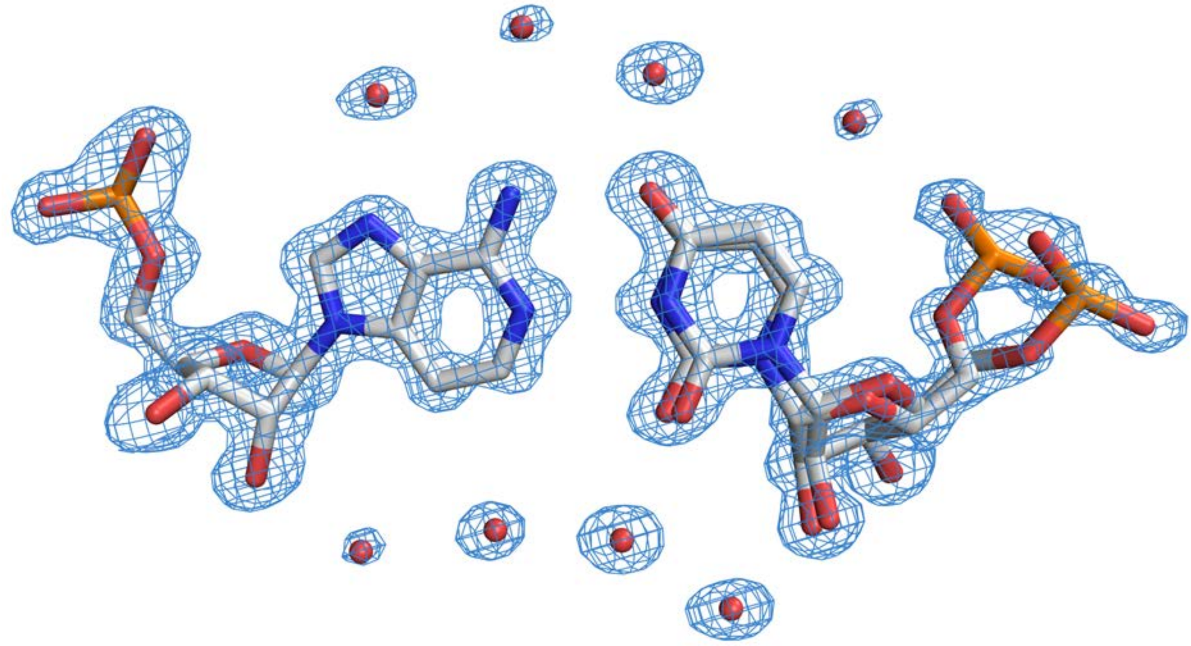

Supporting Figure 19. Unbiased composite omit map of the 2650-2670 base pair of the SRL RNA (PDB ID 7L3R).

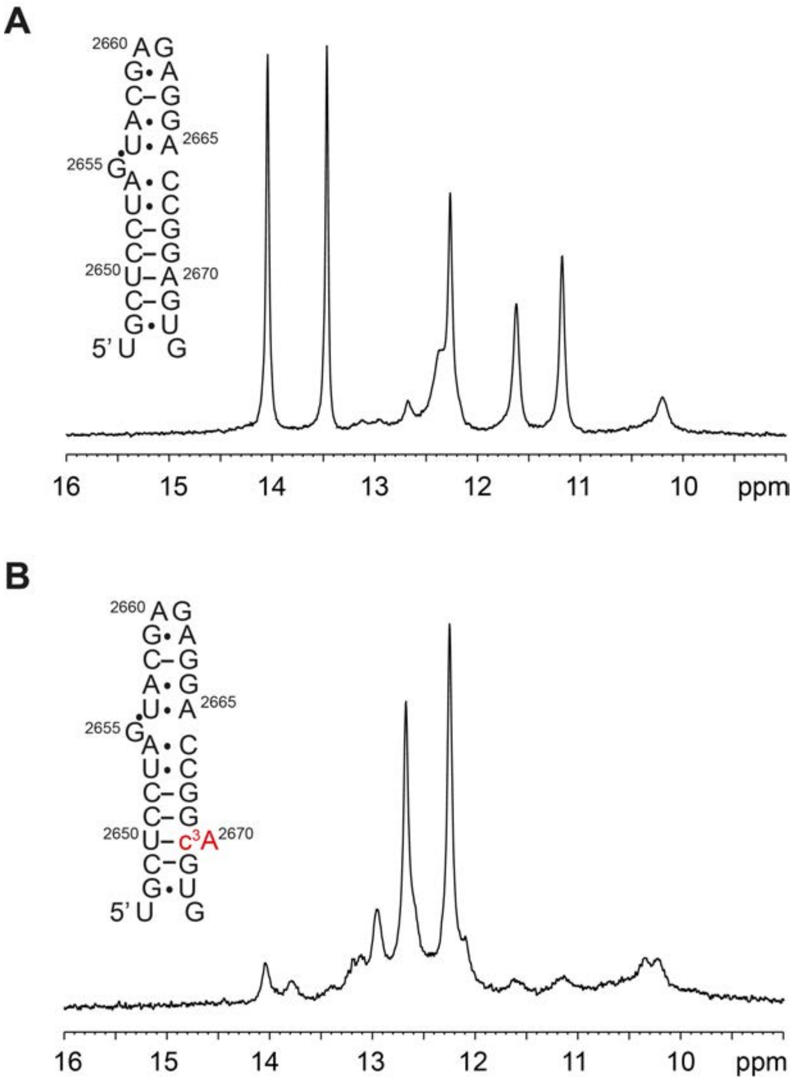

Supporting Figure 20. Comparative ^{1}H NMR imino proton spectra of (**A**) unmodified and (**B**) c^{3}A-modified SRL RNA; conditions: c_{RNA} = 0.1 mM, 15 mM Na[$AsO_2(CH_3)_2$]• $3H_2O$, 25 mM NaCl, 3 mM NaN_3, in D_2O, pH 6.5, 298 K.

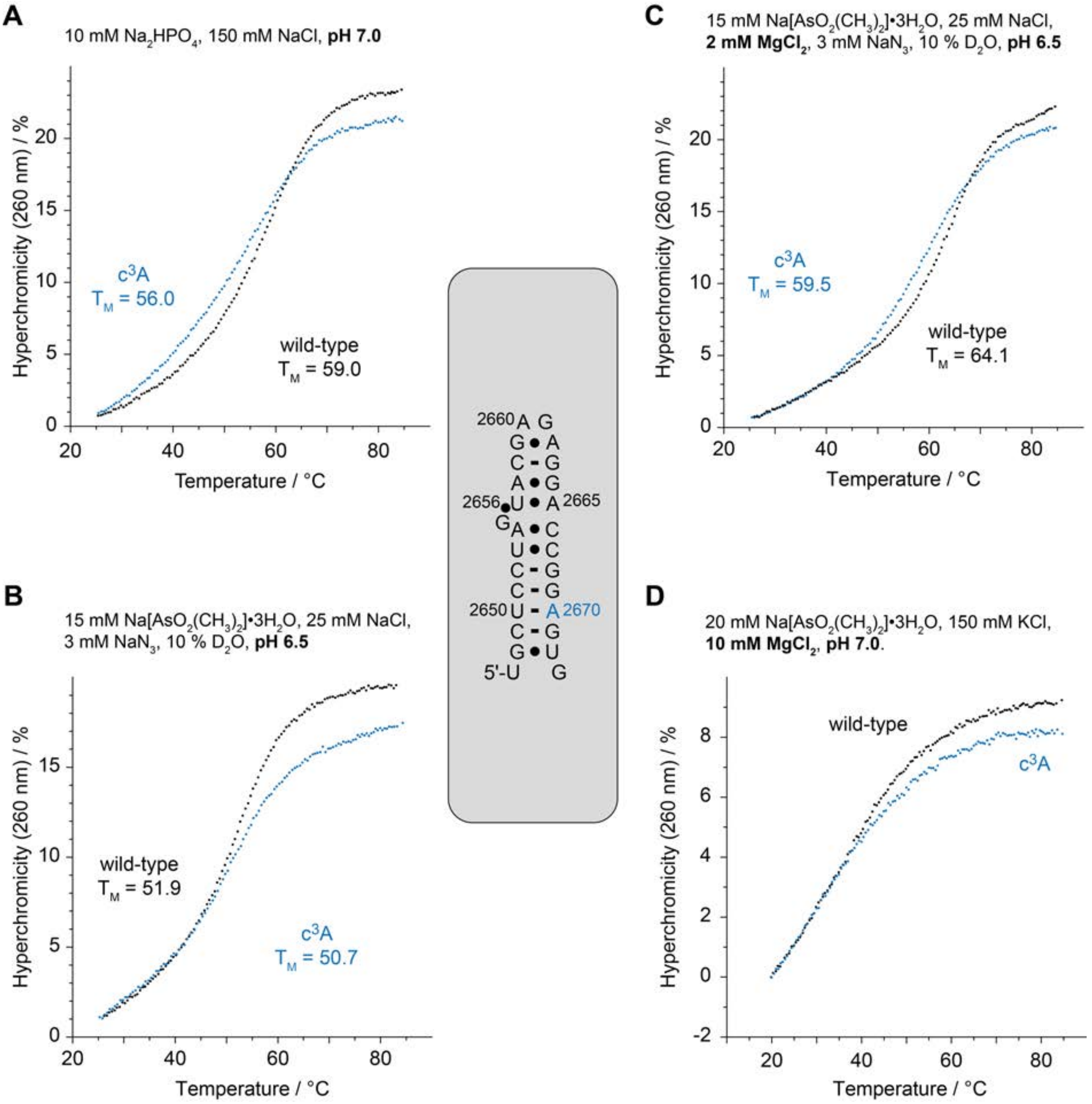

Supporting Figure 21. Comparison of UV melting curves of the Sarcin-Ricin (SRL) RNA measured under different conditions and a RNA concentration c(RNA) = 5 µM. Measurements performed in **A**) typical UV-melting buffer, **B**) typical NMR buffer, **B**) typical NMR buffer supplemented with Mg^{2+} ions ("physiological conditions"), and **D**) alternative physiological conditions according to reference 3.

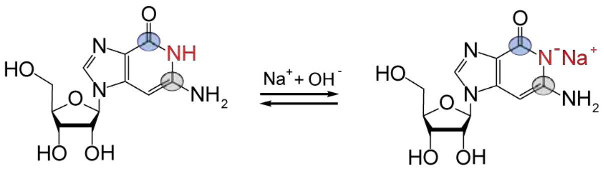

pH	chemical shift ¹³C (C6)/ ppm	chemical shift ¹³C (C2)/ ppm
6.96	157.30	147.24
7.63	157.31	147.25
8.29	157.31	147.25
8.83	157.32	147.26
9.23	157.34	147.27
9.76	157.39	147.33
10.52	157.71	147.68
11.27	159.02	149.13
11.48	159.90	150.25
11.81	161.20	151.70
12.18	162.10	152.59
12.81	163.16	153.67

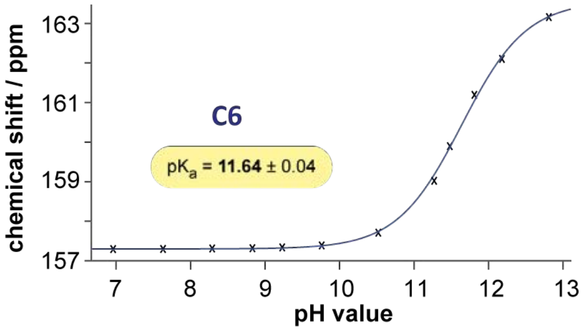

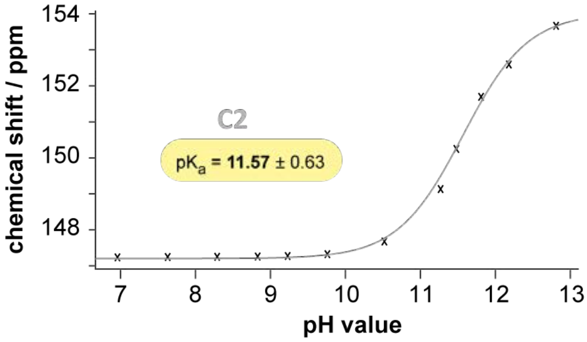

Supporting Figure 22. NMR spectroscopic determination of the pK_a value of the c^3G nucleoside. Chemical structures of the c^3G deprotonation/protonation equilibrium and list of pH-dependent ^{13}C chemical shifts of C6 and C2 (left); plots of ^{13}C chemical shift of C6 (top right) and C2 (bottum right) *versus* pH value.

References

[1] Mairhofer, E.; Flemmich, L.; Kreutz, C.; Micura, R. *Org. Lett.* **2019**, *21*, 3900–3903.

[2] Ohgi, T.; Masutomi, Y.; Ishiyama, K.; Kitagawa, H.; Shiba, Y.; Yano, J. *Org. Lett.* **2005**, *7*, 3477–3480.

[3] Kierzek, E.; Kierzek, R. *Nucleic Acids Res.* **2003**, *31*, 4472–4480.
